# Attention to quantum complexity

**DOI:** 10.1126/sciadv.adu0059

**Published:** 2025-10-10

**Authors:** Hyejin Kim, Yiqing Zhou, Yichen Xu, Kaarthik Varma, Amir H. Karamlou, Ilan T. Rosen, Jesse C. Hoke, Chao Wan, Jin Peng Zhou, William D. Oliver, Yuri D. Lensky, Kilian Q. Weinberger, Eun-Ah Kim

**Affiliations:** ^1^ Department of Physics, Cornell University, Ithaca, NY 14853, USA.; ^2^ Department of Physics, Massachusetts Institute of Technology, Cambridge, MA 02139, USA.; ^3^ Research Laboratory of Electronics, Massachusetts Institute of Technology, Cambridge, MA 02139, USA.; ^4^ Google Research, Mountain View, CA 94043, USA.; ^5^ Department of Physics, Stanford University, Stanford, CA 94305, USA.; ^6^ Department of Computer Science, Cornell University, Ithaca, NY 14853, USA.; ^7^ Department of Electrical Engineering and Computer Science, Massachusetts Institute of Technology, Cambridge, MA 02139, USA.; ^8^ ASAPP, New York City, NY 10007, USA.; ^9^ Department of Physics, Ewha Womans University, Seoul, South Korea.

## Abstract

The imminent era of error-corrected quantum computing demands robust methods to characterize quantum state complexity from limited, noisy measurements. We introduce the Quantum Attention Network (QuAN), a classical artificial intelligence (AI) framework leveraging attention mechanisms tailored for learning quantum complexity. Inspired by large language models, QuAN treats measurement snapshots as tokens while respecting permutation invariance. Combined with our parameter-efficient miniset self-attention block, this enables QuAN to access high-order moments of bit-string distributions and preferentially attend to less noisy snapshots. We test QuAN across three quantum simulation settings: driven hard-core Bose-Hubbard model, random quantum circuits, and toric code under coherent and incoherent noise. QuAN directly learns entanglement and state complexity growth from experimental computational basis measurements, including complexity growth in random circuits from noisy data. In regimes inaccessible to existing theory, QuAN unveils the complete phase diagram for noisy toric code data as a function of both noise types, highlighting AI’s transformative potential for assisting quantum hardware.

## Teaser

Quantum Attention Network leverages the power of attention mechanism to learn quantum complexity.

## INTRODUCTION

Artificial intelligence (AI) and quantum information science are among the most active areas in cutting-edge science and technology, addressing the computational complexity frontier. Although these two domains have evolved separately in the past, recent breakthroughs in both fields create a unique opportunity to use AI to learn quantum complexity. The most paradigm-shifting aspect of the latest large language models, such as ChatGPT, is their generality: Generally trained big models can reason in many different complex settings using natural languages. As quantum hardware platforms enter an era with error correction within reach (*1*–*3*), a general-purpose method for deciphering quantum states with unprecedented levels of complexity and entanglement is critically needed. We ask a compelling question: Can the core mechanism of the large language model’s success, the attention mechanism (*4*, *5*), drive a general-purpose machine for learning quantum complexity? The answer to this question will hinge upon whether an intelligent use of the attention mechanism can hit the core aspects of the quantum complexity using only a polynomial in system size number of measurements from noisy devices.

**Fig. 1.**
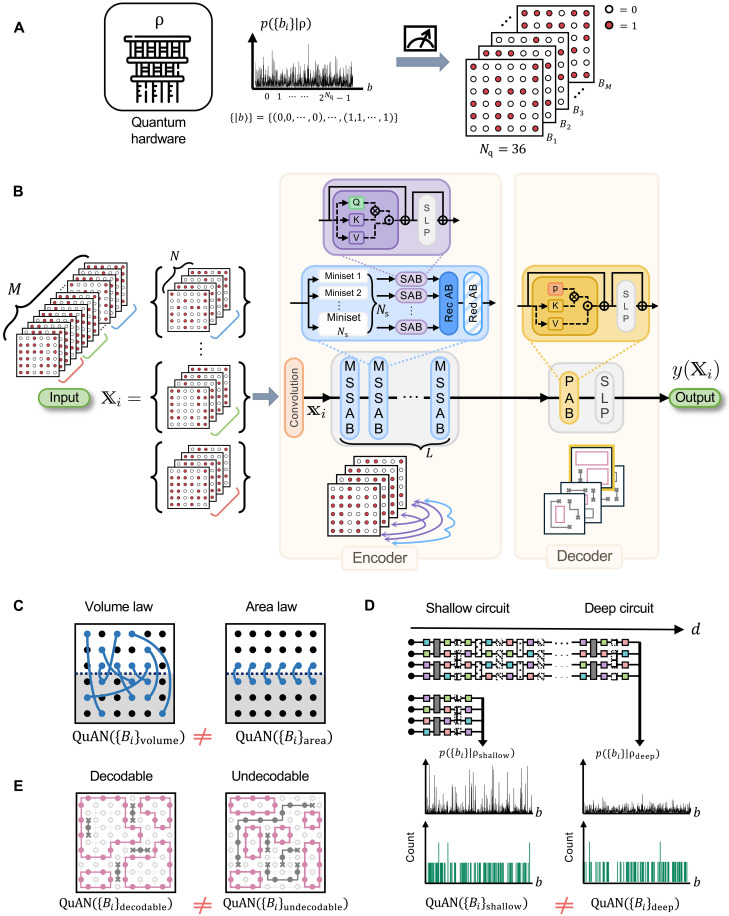
Learning relative complexity between states $\rho_{\alpha }$ and $\rho_{\beta }$ from bit-string collections. (**A**) Measurements of a quantum state ρ samples bit-strings $\left\{\;B_{i}\;\right\}$ from bit-string probability distribution $p\left(\{b_{i}\}|\rho \right)$ over the $2^{N_{q}}$-dimensional Hilbert space. (**B**) Schematic architecture of the QuAN. The Z-basis snapshot collection of size M is partitioned into sets $\left\{\;X_{i}\;\right\}$ of size N . In the encoder stage, after convolution registers positions of qubits, the set goes through L layers of the MSSAB. Inside the MSSAB, the input is further partitioned into $N_{s}$ minisets to be parallel processed through SABs, recurrent attention block (RecAB), and reducing attention block (RedAB). The decoder stage compresses the output from the encoder, allowing for attending to different components in a permutation-invariant manner, using a PAB and single-layer perception (SLP). The output label is y=1 for the state $\rho_{\alpha }$ and y=0 for the state $\rho_{\beta }$ . See Supplementary Materials section A for more details. (**C** to **E**) Examples of $\rho_{\alpha }$ and $\rho_{\beta }$ for learning relative complexity using the binary classification output of the QuAN. (C) Volume-law entangled state versus area-law entangled state. The entanglement between two subsystems (white and gray) is indicated through blue links. (D) Random circuit state at depth d versus that at some deep reference depth. (E) Decodable versus undecodable states of an error-correcting code under noise. The incoherent noise depicted in gray suppresses large loops.

Quantum complexity, once an abstract information-theoretical concept, has become a lynchpin at the intersection of multiple subfields: the study of black holes and geometric theory of wormholes (*6*–*8*), quantum information (*9*–*11*), topological states (*12*–*14*), and thermalization studies (*15*–*20*). Relative complexity between two states represented by density matrices $\rho_{\alpha }$ and $\rho_{\beta }$ aims to quantify the difficulty of producing one from the other. For pure states ∣α〉 and ∣β〉 , Brown and Susskind (*7*) defined it in terms of circuit complexity, i.e., the minimal number of gates required to implement a unitary U connecting the states ( ∣β〉=U∣α〉 ). Although helpful in exploring quantum chaos, directly applying the definition by computing an efficient circuit is often impractical. The challenge is further aggravated by the fact that experimental systems are invariably in mixed states. Theoretical extensions like purification complexity (*21*) and complexity entropy (*22*) demand additional quantum computations, complicating practical use. In practice, our knowledge of states $\rho_{\alpha }$ and $\rho_{\beta }$ is limited to the collection of their measurement bit-string outcomes, ${\left\{B_{j}\right\}}_{\alpha }$ and ${\left\{B_{j}\right\}}_{\beta }$ , and the operations done to the states just before measurements (see Fig. 1A). From a data-centric point of view, the task of learning the relative complexity between $\rho_{\alpha }$ and $\rho_{\beta }$ from available data is a binary classification task of distinguishing

$${\left\{B_{j}\right\}}_{\alpha }\;\text{vs}\;{\left\{B_{j}\right\}}_{\beta }$$ (1)

where j=1,…,M , with the sample size M.

Expectation values of observables, such as order parameters, are the most common and classic choice for comparing two states $\rho_{\alpha }$ and $\rho_{\beta }$ . Even when the observable is not known a priori, finding characteristic spatial motifs using machine learning has been powerful when the two states can be distinguished by quantities linear in density matrices (*23*–*29*). At the opposite extreme of ambition for learning to characterize a state ρ is to model the full probability distribution of bit-strings associated with the given state ρ in the space of $2^{N_{q}}$ possibilities

$$p\left(\{b_{i}\}\;|\;\rho \right),\;i=1,\ldots ,2^{N_{q}}$$ (2)

where $N_{q}$ is the number of qubits, from the measurement data $\left\{\;B_{j}\;\right\}$ . While the advantage of generative modeling has been explored (*30*–*35*), such modeling is ultimately restricted to relatively small systems. A natural middle ground would be to learn key features of the probability distribution in Eq. 2 through moments of the distribution. This approach can be motivated both from a purely statistical perspective and from the perspective of the analysis of local chaotic dynamics’ approach to purely random evolution (*15*, *36*, *37*).

Here, we introduce the Quantum Attention Network (QuAN) shown in Fig. 1B as a general-purpose AI for a classical computer to learn relative quantum complexity between two states $\rho_{\alpha }$ and $\rho_{\beta }$ through binary classification between the measurement bit-strings ${\left\{B_{j}\right\}}_{\alpha }$ and ${\left\{B_{j}\right\}}_{\beta }$ . The QuAN capitalizes on the fact that the self-attention mechanism (*4*) learns the varying significance of the correlation between words at arbitrary distances within a sentence. However, while words in a sentence form a sequence, the order of bit-strings generated by identical experiments has no meaning. Through multiple layers of self-attention blocks (SABs) attending between bit-strings in a manner that is manifestly permutation invariant, the QuAN approximately learns high moments of the distribution $p\left(\{b_{i}\}\;|\;\rho \right)$ from a finite number of samples (see Supplementary Materials section A for more details). Furthermore, by attending to snapshots less affected by noise, the QuAN extracts target features of pure state in the presence of a finite amount of noise. In the rest of the paper, we present the design principles of the QuAN guided by the learning goals and demonstrate the QuAN’s efficacy as a general-purpose AI by deploying QuAN to learn relative complexity between two states $\rho_{\alpha }$ and $\rho_{\beta }$ that differ in ways that are fundamental to three frontiers: entanglement transition, chaotic dynamics, and error correction, as sketched in Fig. 1 (C to E).

Figure 1B shows the QuAN architecture and the data flow through QuAN. QuAN takes a set of snapshots $X_{i}$ as input and outputs the machine’s confidence $y\left(X_{i}\right)\in \left[0,1\right)$ on the input belonging to class α between the binary choice of α and β . To attend between snapshots, we first partition the complete data consisting of M snapshots into sets consisting of N snapshots. Typically, we train with 70 to 75% of data and validate with the rest (see Supplementary Materials sections C1, D1, and E1 for more details). Each set $X_{i}$ then goes through three stages in sequence: convolution, encoder, and decoder stages (see Fig. 1B). The convolution stage incorporates local spatial features and maps the binary-valued $X_{i}$ to vectors $x_{i}$ with continuous entries with better algebraic properties for sampling moments. In language models, the encoding stage transforms the input into an informative, learned representation. For QuAN’s encoding to target moments of $x_{i}$ , we introduce miniset self-attention blocks (MSSABs). Unique to the QuAN architecture, the MSSAB is adapted from a permutation-invariant version of the transformer (*38*) to access high moments of the distribution in a parameter-efficient manner. It accesses up to $2N_{s}^{2}$ order correlations for $N_{S}$ minisets within one layer.

The decoder stage consists of the pooling attention block (PAB) and the single-layer perceptron that compresses all the information into the confidence output (see Supplementary Materials section A4). The PAB layer learns to attend more to the snapshots with features characteristic of the target state. When the confidence is $y\left(X_{i}\right)<0.5$ , the QuAN assesses the data to belong to state α ; otherwise, it is assessing the data to belong to state β . We train the QuAN by minimizing binary cross-entropy loss between the ground truth and QuAN’s prediction through Adam optimization. The multifaceted use of attention mechanisms empowers the QuAN to be a versatile general-purpose AI capable of learning quantum complexity in various datasets.

## RESULTS

**Fig. 2.**
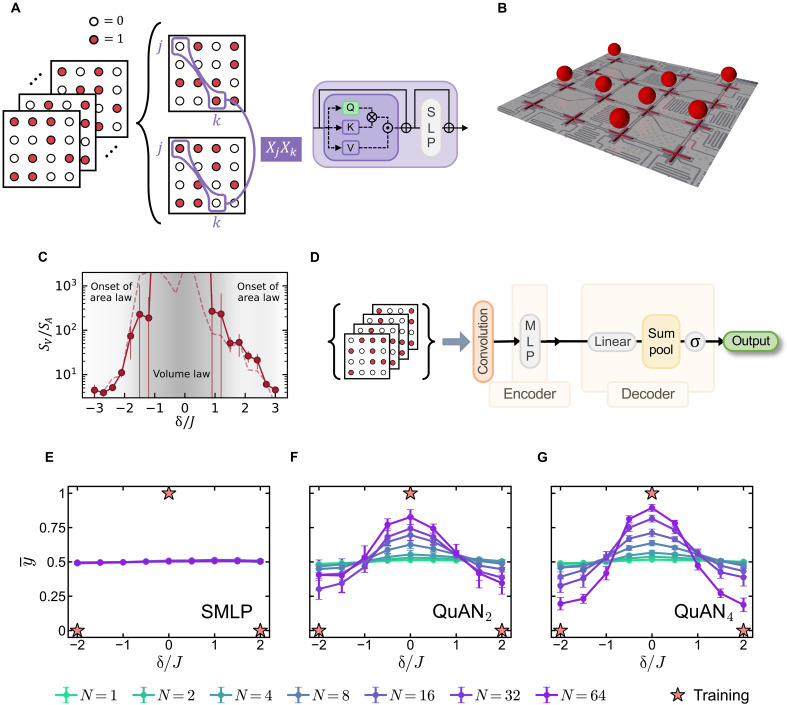
Relative complexity between volume-law and area-law scaling states. (**A**) Intersnapshot correlation reveals X-X correlation of the quantum state. The purple box shows the schematic of the SAB capturing the intersnapshot correlation. (**B**) Schematic diagram of the 16-transmon-qubit chip used for quantum emulation of the driven hard-core Boson-Hubbard model. (**C**) Entanglement transition based on the scaling of bipartite entanglement entropy $S=S_{A}A+S_{V}V$ , where A and V represent the area and volume of the subsystem, respectively. Adapted from Karamlou *et al*. (*45*) (https://creativecommons.org/licenses/by/4.0/). (**D**) Schematic of a contrast architecture: The SMLP respects the permutation symmetry. (**E** to **G**) Average confidence $\bar{y}$ as a function of detuning strength δ for different architectures using different set sizes N . The star symbol marks the training points. The average and errors are obtained from 10 independent model training. For machine learning details, see Supplementary Materials section C2. (E) The SMLP fails to train. (F) QuAN_2_ ( $N_{s}=1$ , L=1 ). (G) QuAN_4_ with two layers of self-attention ( $N_{s}=1$ , L=2).

First, we consider the relative complexity between states with different entanglement scaling: a volume-law scaling state and an area-law scaling state (see Fig. 1C). The distinction is substantial because classical computers cannot efficiently represent a general volume-law scaling state. Moreover, the change in entanglement scaling signals measurement-induced phase transitions (*39*–*44*). Nevertheless, extraction of the entanglement scaling is often challenging because it requires randomized multibasis measurements or state tomography for subsystems with varying sizes (*45*). Our key insight is that the QuAN can learn the change in the entanglement scaling by attending between snapshots within the set (see Fig. 2A). The self-attention score for the set $X_{i}$ in each SAB is given by

$$\left\langle \;{Qx}_{i}|{Kx}_{i}\right\rangle =\left({Qx}_{i}\right){\left({Kx}_{i}\right)}^{T}$$ (3)

up to normalization, where $x_{i}$ is convolved from the snapshot set $X_{i}$ as shown in Fig. 1B; *Q* and *K* are two trainable transformation matrices, often referred to as query ( Q ) and key ( K ) (see Supplementary Materials section A3a for more details). When two Z-basis snapshots are related by simultaneous bit-flips at a pair of qubits (j,k) , the attention score reflects the correlation $\langle X_{j}X_{k}\rangle$ , which is upper-bounded by their mutual information (see Fig. 2A and also Supplementary Materials section C4) (*46*). Hence, the QuAN can access Pauli X correlations from Z-basis measurement through intersnapshot attention. In an area-law state, an X correlation is concentrated between nearby qubits. On the other hand, in a volume-law state, the entanglement of each qubit shared with the entire system dilutes the X correlation. By accessing the X correlation through intersnapshot attentions, the QuAN may witness the entanglement transition from just Z-basis snapshots.

To verify QuAN’s potential for witnessing the entanglement transition from the Z−basis measurements, we turn to coherent-like states prepared to be a superposition of hard-core Bose Hubbard model energy eigenstates reached through a driven Hamiltonian

$$H/\hbar =\sum_{\langle j,k\rangle }J_{\mathrm{jk}}{\hat{\sigma }}_{j}^{+}{\hat{\sigma }}_{k}^{-}+\frac{\delta }{2}\sum_{j}{\hat{\sigma }}_{j}^{z}+\Omega \sum_{j}\left(\alpha_{j}{\hat{\sigma }}_{j}^{-}+h.c.\right)$$ (4)

implemented using a superconducting, transmon-based quantum simulator (see Fig. 2B). Here, ${\hat{\sigma }}_{j}^{+}\left({\hat{\sigma }}_{j}^{-}\right)$ represents the raising (lowering) operator on qubit at site j , and ${\hat{\sigma }}_{j}^{z}$ represents the Pauli Z operator at site j ; $J_{\mathrm{jk}}$ is the particle exchange interaction strength between sites j and k of average value J , δ is the detuning between the drive and qubit frequency, and Ω is the drive strength (see Materials and Methods). Upon tuning δ/J , the system prepares a coherent-like superposition of states at the center of the spectrum for small δ/J and that of states at the edge of the spectrum for large δ/J (*45*). Bipartite entanglement entropy calculated from subsystem tomography using subsystem measurements in an informationally complete basis set found the volume-law scaling at low values of ∣δ∣/J and the area-law scaling at large values of ∣δ∣/J (see Fig. 2C). Here, we use the experimental snapshots of the entire system in the particle number basis, which maps to Z-basis measurements in the hard-core limit over a range of δ/J.

To investigate the QuAN’s capability and the role of “attention” in witnessing the entanglement transition, we compared the performance of three different architectures with varying degrees of attention. The simplest architecture is the set multilayer perceptron (SMLP) without the SABs and PABs (see Fig. 2D). The SMLP is a generalization of the usual multilayer perception (*47*) designed to take a set of snapshots as input and learn the positional information through convolution. We set the miniset size for the other two architectures to $N_{s}=1$ , which reduces the MSSAB to a single SAB. QuAN_2_ and QuAN_4_ each access up to the second and fourth moments through SAB layers (see Supplementary Materials section A3d).

The task for the three architectures is to witness the change in the entanglement scaling upon an increase in δ/J by training the models to distinguish the δ/J=0 state from the δ/J=2 state using snapshots with the same number ( n=8 ; see Supplementary Materials section C1) of bosons. All three architectures were trained and tested using M=69,632 experimental snapshots from δ/J=0 and the same number of snapshots from δ/J=±2 with the binary label: y=1 for data from δ/J=0 with volume-law entanglement and y=0 for data from δ/J=±2 with area-law entanglement.

Figure 2 (E to G) compares the performance of the three architectures on the basis of 10 independent training for each architecture. When an architecture learns to witness the entanglement transition, average confidence $\bar{y}={\langle y\left(X_{i}\right)\rangle }_{i}$ should span between $\bar{y}=1$ at δ/J=0 and $\bar{y}=0$ at δ/J=2 (see Supplementary Materials section C2). Figure 2E shows that the SMLP fails to learn the entanglement transition, given the average confidence remaining flat at $\bar{y}=0.5$ independent of set size N (see Fig. 2E). By contrast, QuAN_2_, which only uses second moments of bit-strings, already learns the entanglement transition once a set structure is used. Moreover, its learning improves with increasing set size within the bounds of the total sample size (see Fig. 2F and see Supplementary Materials section C3). Accessing up to the fourth moment, QuAN_4_ shows sharper transition with reduced error bars across different training. The above comparison showcases the QuAN’s ability to witness changes in entanglement scaling using Z-basis measurements alone through intersnapshot attention.

**Fig. 3.**
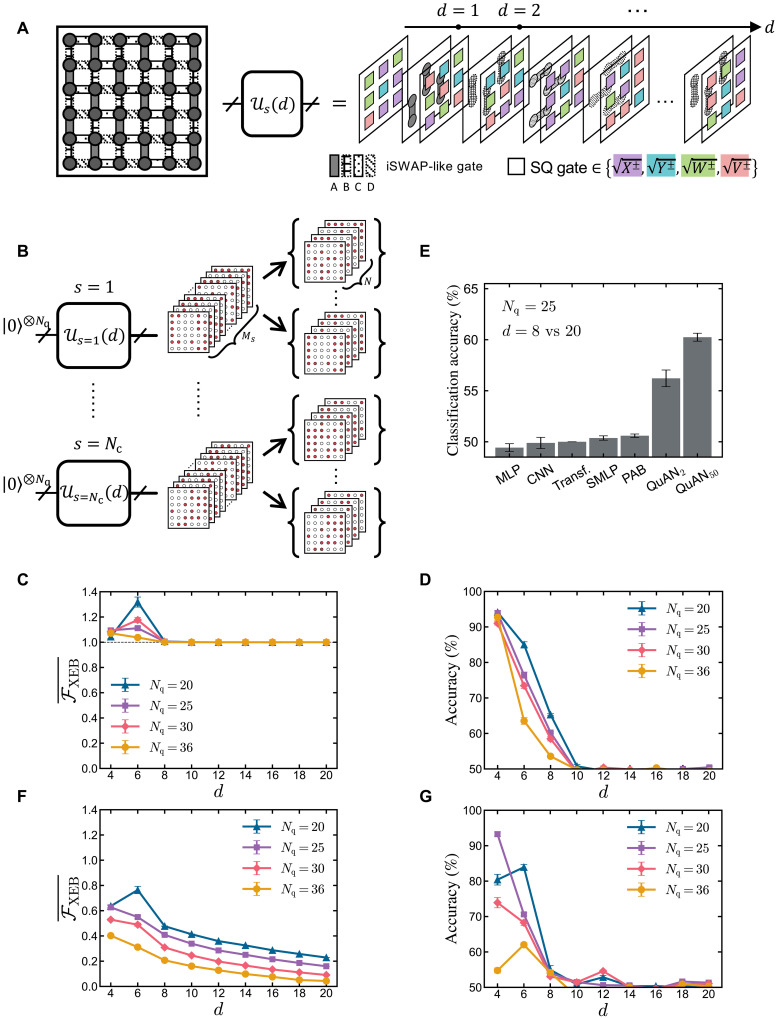
Relative complexity between the random circuit state at depth *d* and the reference state at depth *d* = 20. (**A**) Schematic illustration of the 6-by-6 subarray of qubits from Google’s “Sycamore” quantum processor. A random circuit of depth d alternates entangling iSWAP-like gates (gray) and single-qubit (SQ) gates randomly chosen from the set $\left\{\;\sqrt{X^{\pm 1}},\sqrt{Y^{\pm 1}},\sqrt{W^{\pm 1}},\sqrt{V^{\pm 1}}\right\}$ , with $W=\left(X+Y\right)/\sqrt{2}$ and $V=\left(X-Y\right)/\sqrt{2}$ . The two-qubit gates are applied in a repeating series of ABCDCDAB patterns. (**B**) Data structure. For each depth d , we sample $N_{c}=50$ circuits. For each circuit instance s , we sample $M_{s}$ bit-strings and partition them into sets of size N , resulting in a total of $N_{c}\times M_{s}/N$ sets for each circuit depth d . (**C**) XEB (Eq. 5) for bit-strings from noiseless simulations as a function of circuit depth d with varying system sizes $N_{q}$ . The markers show the averaged XEB over $N_{c}=50$ different circuit instances and the error bars for the standard errors. (**D**) Pure-state trained QuAN_50_’s classification accuracy for pure-state data. We train eight independent models at each circuit depth d and show the averaged accuracy (marker) and the standard error (error bar). QuAN_50_ successfully learns the relative complexity of d=8 . (**E**) Comparison of the performances of QuAN_2_, QuAN_50_, and other architectures in learning the relative complexity of depth d=8 on an $N_{q}=25$ qubit system. The models maintain approximately the same total number of trainable parameters to make a controlled comparison between different architectures. (**F**) Averaged XEB for experimentally collected bit-strings. The plot shows the averaged XEB over 50 circuit instances (markers) and the standard error (error bars). The XEB smoothly decays as a function of depth d . (**G**) Learning relative complexity from experimental data using QuAN_50_ trained on noiseless data.

Next, we move on to learning the relative state complexity between shallow and deep random circuit states: $\rho_{\text{shallow}}$ versus $\rho_{\text{deep}}$ . The random circuits have become a theoretical paradigm for studying diverse sets of phenomena in quantum dynamics from information processing in black holes (*48*) to quantum chaos and thermalization (*15*, *49*–*51*). Despite being composed of local gates (see Fig. 3A), the ensemble of random circuits accurately approximates the ensemble of global random unitary transformations up to the first k-moments, where k grows with the depth of the circuit (*36*). Furthermore, the circuit complexity, which refers to the minimal number of gates required to evolve a fiduciary reference state to a state implemented by a given random circuit, is known to grow linearly with k (*15*). However, how to find such k for a particular random circuit from a finite amount of data is not known. Instead, we adopt our data-centric approach, which involves learning the relative complexity between the state reached at depth d and a reference deep circuit at d=20 from the QuAN’s accuracy at distinguishing data from the two states. We aim to learn the evolution of relative state complexity of states reached by random circuits as a function of the circuit depth by using the QuAN for multiple pairwise classification tasks that contrast data from variable depth d with data from d=20 . Training the QuAN to perform this classification probes its ability to learn higher-order features of the bit-string data, like aspects of the third and higher moments of the bit-string distribution (*15*), which reflect the relative complexity of the state at depth d compared to the state at depth d=20 . We anticipate that such relative complexity will shrink with increasing depth d . To coalesce the QuAN’s learning across different training runs for each depth, we study the classification accuracy defined as the percentage of correctly classified inputs (by correct classification, meaning the machine’s confidence in the correct labels exceeding 0.5).

While previous experiments primarily focused on deep circuits pushing against the limits of classical simulation, we systematically explore the increase in complexity as the depth increases. We consider both experimental data from quantum hardware (see Materials and Methods for experimental details) and data from the corresponding pure state implemented on classical hardware. At each circuit depth d , we sample $N_{c}=50$ random circuit instances. For each circuit instance, we collect $M_{s}=M/N_{c}=500,000$ bit-strings ( $M_{s}=2,000,000$ for $N_{q}=36$ ). To prevent the QuAN from overfitting a specific circuit instance, we train the QuAN using data from 70% of all the circuit instances and reserve the remaining 30% circuits for testing (see Supplementary Materials section D1). We batch bit-strings from the same circuit instance into sets of N bit-strings, shown in Fig. 3B. See Supplementary Materials section D2 for more details on training and testing.

We benchmark the QuAN’s learning of relative complexity at each circuit depth d against the depth dependence of the linear cross-entropy benchmark (XEB). The linear XEB, $F_{\text{XEB}}$ , for a collection of bit-strings $\left\{\;B_{j}\;\right\}$ is defined by

$$F_{\text{XEB}}\left[\{B_{j}\}\right]=2^{N_{q}}{\langle p_{U}\left(B_{j}\right)\rangle }_{j}-1$$ (5)

where $p_{U}\left(B_{j}\right)$ is the probability for the given bit-string $B_{j}$ to be sampled from an ideal pure state $U|0\rangle^{⊗N_{q}}$ for the random unitary circuit U of depth d . We first study the depth-dependent relative complexity of the ideal pure-state data. When $\left\{\;B_{j}\;\right\}$ is sampled from the same ideal pure state, $F_{\text{XEB}}\left[\{B_{j}\}\right]$ measures the second moment of the bit-string distribution associated with the pure state. As shown in Fig. 3C, the XEB for such bit-string saturates the infinite depth asymptotic value of $F_{\text{XEB}}=1$ to polynomial precision near depth d=8 for all system sizes considered, becoming blind to circuit dynamics past d=8 . The relative complexity learned by the QuAN quantified through classification accuracy shrinks upon increasing depth as expected, approaching the vanishing relative complexity at 50% accuracy of the classification task. Nevertheless, QuAN_50_ that accesses up to the 50th moment (see Supplementary Materials section A3) learns the relative complexity of d=8 for all system sizes (see Fig. 3D). Furthermore, the classification accuracy at d=8 shows promising subexponential scaling with the increase in the system size (see Supplementary Materials section D4 for more details). Given that the QuAN learns the relative complexity, we present a visualization of the relative XEBs considering the differences between XEBs from two circuit depths in Supplementary Materials section D3 as a benchmarking baseline.

Comparing various architectures’ learning of relative complexity at depth d=8 shown in Fig. 3E reveals how difficult this task is for most architectures. Despite all having the same number of hyperparameters, architectures other than the QuAN failed to learn the relative complexity of d=8 and 20. Notably, the failure of three architectures that took individual bit-strings without forming a set structure [the MLP, convolutional neural network (CNN), and standard transformer (Transf.); see Supplementary Materials section B] establishes the importance of the set structure for learning relative complexity. The failure of the SMLP and PAB shows that using a set structure is not enough without self-attention. With just a single layer of MSSAB, the QuAN learns the relative complexity of depth d=8 (QuAN_2_ in Fig. 3E). Allowed to learn up to the 50th moment, QuAN_50_’s accuracy shoots up to demonstrate the benefit of MSSAB accessing higher moments. We refer curious readers to Supplementary Materials section D6 for a detailed analysis addressing how the maximal accessible order of moments affects the QuAN’s learning. In the rest of the paper, we focus on the performance of QuAN_50_ with a single MSSAB block ( L=1 ) containing $N_{s}=5$ minisets.

The advantage of the QuAN’s relative complexity learning is further pronounced when the bit-strings, $\left\{\;B_{j}\;\right\}$ , are experimentally sampled. For such data, the XEB $F_{\text{XEB}}\left[\{B_{j}\}\right]$ estimates the correspondence between experimental snapshots and the snapshot distribution of the ideal circuit up to the second order. As shown in Fig. 3F, $F_{\text{XEB}}\left[\{B_{j}\}\right]$ for the experimental data smoothly evolves to 0, reflecting the increase in noise with increasing depth, which pushes the system closer to the maximally mixed state rather than the intended pure state. Dominated by the noise, $F_{\text{XEB}}$ on experimental data does not reveal the increase in complexity driven by the intended unitary evolution. The QuAN also confirms the notable influence of increasing noise when the depth dependence of relative complexity is probed by training and testing the QuAN entirely on experimental data (see Supplementary Materials section D4). The key question is whether learning the remnants of the pure state dynamics from noisy experimental data is possible.

One way to learn the aspect of relative complexity that corresponds to pure state dynamics from the experimental data is to quantify the relative complexity of experimental bit-strings using the QuAN trained on noiseless pure state data. Unexpectedly, the depth dependence of the pure-state trained QuAN’s classification accuracy of experimental data in Fig. 3G shows a trend that closely follows corresponding depth dependence in its inspection of the noiseless simulated data shown in Fig. 3D. Specifically, they both exhibit a transition at depth d=10 . Hence, the QuAN was able to reveal traces of pure-state complexity evolution from noisy experimental data.

Overcoming noise in relative complexity learning is particularly important for noisy states from error-correcting codes. As the quantum hardware approaches the breakeven point, the community is increasingly focused on learning the topological order, a phase of matter that supports quantum error correction, in a mixed state (*12*, *52*–*56*). The earlier literature on the complexity of topological states focused on the relative complexity of topological states compared to a simple product state $|\;0\rangle^{⊗N_{q}}$ . It is known that topological states’ relative complexity with respect to $|\;0\rangle^{⊗N_{q}}$ grows linearly with the system size. However, much less is known about the relative complexity between a topological state and an undecodably noisy state.

One leading candidate for fault-tolerant quantum memory is the toric code or ${\mathbb{Z}}_{2}$ topological order. In its ideal ground state ∣TC〉 , a closed Z-loop operator around a loop γ , $Z_{\text{closed}}\left(\gamma \right)\equiv \prod_{i\in \gamma }Z_{i}$ , has a length-independent expectation value

$$\langle \text{TC}|Z_{\text{closed}}\left(\gamma \right)|\text{TC}\rangle =1$$ (6)

Infinitesimal noise introduces tension to the above loop operator expectation value, resulting in its exponential decay with the loop perimeter (*57*), as illustrated in Fig. 1E for incoherent noise. Hence, any amount of noise destroys the topological order from the loop tension perspective. Nevertheless, mapping the limiting cases to statistical mechanics models reveals hidden phase transitions. Specifically, with just the coherent noise $g_{X}$ modeled through

∣Ψ(gX)〉=1Nexp(gX∑iXi)TC (7)

where N is a normalization factor; the error threshold of $g_{X}\approx 0.22$ was established in (*58*) via mapping the problem to a classical two-dimensional (2D) Ising model. Alternatively, with just an incoherent noise $p_{\text{flip}}$ modeled through the bit-flip error channel

$$E_{i}\left(\rho \right)=\left(1-p_{\text{flip}}\right)\rho +p_{\text{flip}}X_{i}\rho X_{i}$$ (8)

for each qubit *i*; the error threshold of $p_{\text{flip}}\approx 0.11$ was established in (*52*) via mapping the error model to the random bond Ising model (*59*). However, the phase diagram interpolating between the two axes has yet to be achieved. Motivated by the QuAN’s successes, we use the QuAN to solve this open problem.

**Fig. 4.**
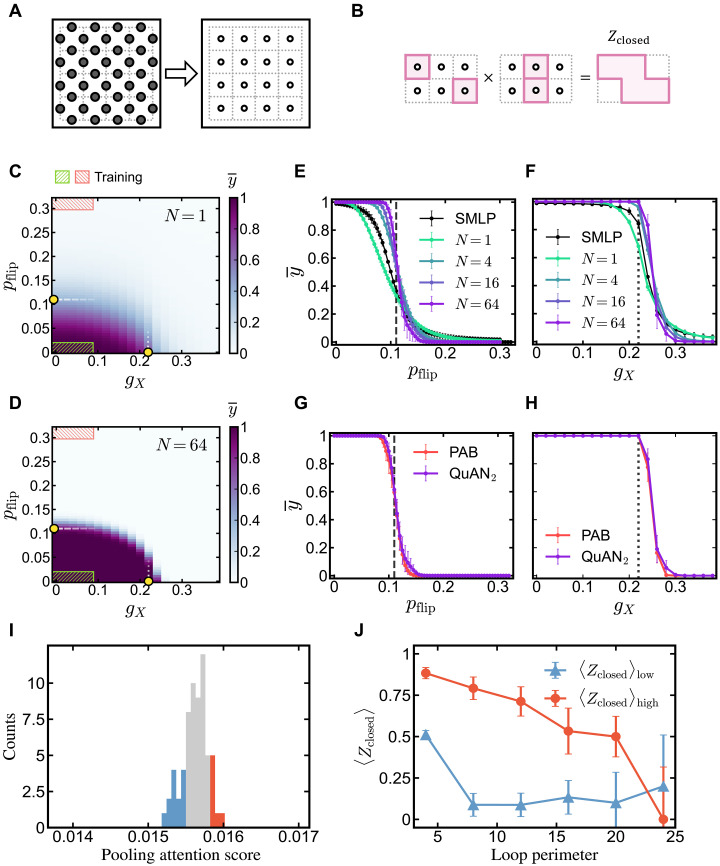
Learning the relative complexity of decodable and undecodable states of the toric code. (**A**) Transformation from the Z-basis measurements to the smallest-loop, plaquette variables. (**B**) The QuAN can build larger closed loops through multiplication. (**C** and **D**) Decodability phase diagram of the toric code state under coherent and incoherent noise for two different set sizes: N=1 in (C) and N=64 in (D). The regions in the phase space that support the training data are marked with hatch marks. The average confidence $\bar{y}$ averages over 10 independent model training. The known thresholds are marked along the $g_{X}=0$ axis at $p_{c}\approx 0.11$ and along the $p_{\text{flip}}=0$ at $g_{c}\approx 0.22$ . (**E**) Average confidence $\bar{y}$ by QuAN_2_ for different set sizes N and by the SMLP with N=64 along the axis $g_{X}=0$ . The error bar shows the standard error for $\bar{y}$ over 10 independent model training. (**F**) Average confidence $\bar{y}$ by QuAN_2_ with varying set sizes N and by the SMLP with N=64 along the axis $p_{\text{flip}}=0$ . (**G**) Average confidence $\bar{y}$ by QuAN_2_ and the PAB with N=64 along the axis $g_{X}=0$ , where the PAB is defined as the model without self-attention and has only pooling attention. (**H**) Average confidence $\bar{y}$ by QuAN_2_ and the PAB with N=64 along the axis $p_{\text{flip}}=0$ . (**I**) Pooling attention score histogram from the topological state with $\left(g_{X},p_{\text{flip}}\right)=\left(0,0.05\right)$ . (**J**) Loop expectation value $\langle Z_{\text{closed}}\rangle$ as a function of the loop perimeter for high- and low-attention-score snapshots in the topological state with $\left(g_{X},p_{\text{flip}}\right)=\left(0,0.05\right)$ . The error bars represent the standard error of $\langle Z_{\text{closed}}\rangle$ over different loop configurations in corresponding snapshots.

To study the effect of coherent and incoherent noise in a controlled way, we use classically simulated toric code ground state modified by coherent noise strength $g_{X}$ , available in (*60*) as a part of Cong *et al*. (*61*). To the Z-basis bit-string data sampled from this state, we implement the error channel (Eq. 8) through random bit-flips (see Materials and Methods). We then transform the resulting bit-strings into measurements of the smallest loops, building on the insight in (*28*). Now, the collection of these plaquette values goes into the QuAN as input. To arrive at the QuAN that interpolates between the two axes of the noise phase space $\left(g_{X},p_{\text{flip}}\right)$ in its learning of the relative complexity between decodable and undecodable states, we train the QuAN with nearly coherent data over the range of $g_{X}$ value and deeply incoherent data over the same range of $g_{X}$ value (see Fig. 4, C and D, and Supplementary Materials section E1). We then classify the data from the rest of the phase space.

Figure 4 (C and D) shows that with a sufficiently large set size, QuAN confidence marks a sharp distinction between decodable and undecodable states, saturating the error threshold for incoherent noise $p_{\text{flip}}\approx 0.11$ . The cut along the $g_{X}=0$ axis (Fig. 4E) shows that the set structure and the attention mechanisms in the QuAN are essential. Upon increasing the set size, the transition is sharpening toward $p_{\text{flip}}\approx 0.11$ along the $g_{X}=0$ axis. With the set size of N=64 , the QuAN observes a sharp transition close to the theoretically predicted coherent noise threshold of $g_{X}\approx 0.22$ along the $p_{\text{flip}}=0$ axis (see Fig. 4F) (*62*). Furthermore, predictions of both phase boundaries are robust against shifts in the training parameter region (see Supplementary Materials section E3a). This is unexpected given that we did not explicitly train the QuAN to contrast $g_{X}=0$ versus large $g_{X}\neq 0$ and warrants further theoretical investigation into the potential existence of a unifying statistical mechanics model.

The model ablation studies (see Fig. 4, G and H, and Supplementary Materials section E3c) revealed the critical role of the pooling attention decoder as an automatic importance sampler with excellent sample complexity. Specifically, we tried removing the self-attention stage entirely, leaving only the pooling attention stage; we refer to the resulting architecture as the PAB. When comparing the average confidence of the PAB architecture shown in Fig. 4 (G and H) with that of QuAN_2_, we observe that the PAB effectively saturates the known thresholds along both axes for the set size N=64 . This allows in-depth interpretation of the machine’s learning because the pooling attention score of the PAB can be traced to individual snapshots $X_{i}$ (see Supplementary Materials section E4).

By comparing the snapshots with high and low pooling attention scores, we can gain insight into the features of the data recognized as that of a topological phase. For this, we inspect the distribution of the pooling attention score across all the snapshots with $p_{\text{flip}}=0.05$ and $g_{X}=0$ shown in Fig. 4I. Selecting the snapshots with top 15% and bottom 15% of the distribution, we analyze the subset average value of the Z-loop operator $Z_{\text{closed}}$ for a closed loop γ as a function of the length of the perimeter (see Fig. 4J). This expectation value can be readily calculated from each subset of Z-basis snapshots as an average. The contrast in the length dependence of the loop expectation value $\langle Z_{\text{closed}}\rangle$ between the high and low attention groups is notable. The snapshots in the high-attention-score group show large $\langle Z_{\text{closed}}\rangle$ with weak perimeter length dependence until the length hits the system size. On the other hand, for the snapshots in the low-attention-score group, $\langle Z_{\text{closed}}\rangle$ decays immediately after the smallest loop perimeter. Hence, it appears that the QuAN learned to selectively attend to snapshots with vanishing loop tension. The QuAN’s importance sampling is a data-efficient alternative to seeking a dressed loop operator (*57*, *61*) with a length-independent expectation value or information-theoretical measures (*12*, *54*, *58*).

## DISCUSSION

To summarize, we introduced the QuAN, a versatile general-purpose architecture for a classical computer that adopts the attention mechanism for learning relative quantum complexity between two states. We choose to restrict the QuAN’s access to Z-basis measurements as such measurements are most broadly accessible to quantum hardware. While this means that off-diagonal elements of the density matrix cannot be learned, our analysis shows that much can be learned from such data when the salient aspects of the bit-string distributions are effectively learned using the QuAN. Specifically, the bit-string distribution for a relatively more complex state compared to a product state will be spread out rather than concentrated. The QuAN can learn such a change in the distribution shape through the moments of the distribution. Also, a volume-law entangled state is more anticoncentrated. Hence, although the QuAN cannot directly learn entanglement from Z-basis measurements, it still witnesses the consequences of the entanglement transition. The QuAN is built on three principles: (i) treat the snapshots as a set with permutation invariance, (ii) attend between snapshots to access higher moments of bit-string distribution, and (iii) attend over snapshots to the importance sample. The QuAN treats each snapshot as a “token” and leverages the capability of stacked L-layers of $N_{s}$ miniset self-attention to sample ${\left(2N_{s}^{2}\right)}^{L}$-th moments of snapshots.

We put the QuAN to work on three challenging sets of Z-basis data to showcase the power of the QuAN and gain insights. With the driven hard-core Bose-Hubbard (HCBH) model data, we found that the entanglement transition between the volume-law and area-law scaling regimes can be witnessed entirely with the Z-basis measurements. With random circuit sampling data, we revealed the evolution of complexity with increasing depth from noisy experimental data that reflect noise-free evolution. Last, with the mixed state data of the toric code with coherent and incoherent noise, we obtained the decodability phase diagram that saturates known error thresholds. The QuAN’s findings set challenges for the theoretical understanding. Simultaneously, the QuAN’s ability to learn relative quantum complexity through the adaptive use of attention mechanisms holds promise for quantum error correction, the key data-centric problem for the application of quantum hardware. While an AI’s learning is typically hard to interpret, we revealed the critical role of self-attention and pulling attention through ablation studies. Promising future directions for the application of QuAN include the development of a decoder for quantum error correction codes and learning measurement-induced phase transitions.

## MATERIALS AND METHODS

### Data acquisition

The data used for learning the entanglement transition in the driven HCBH model is acquired from a 4-by-4 array of superconducting transmon qubits as described in (*45*). In this system, the on-site interaction, determined by the anharmonicity of the transmon qubits, is much stronger than the exchange interaction. Therefore, the system can be described by an HCBH Hamiltonian

$${\hat{H}}_{\text{HCBH}}/\hbar =\sum_{\langle j,k\rangle }J_{\mathrm{jk}}{\hat{\sigma }}_{j}^{+}{\hat{\sigma }}_{k}^{-}-\;\sum_{j}\frac{\epsilon_{j}}{2}{\hat{\sigma }}_{j}^{z}\;$$ (9)

where ${\hat{\sigma }}_{j}^{+}$ ( ${\hat{\sigma }}_{j}^{-}$ ) is the raising (lowering) operator for a two-level system at site j , and ${\hat{\sigma }}_{j}^{z}$ is the Pauli-Z operator. The first term describes the particle exchange interaction between neighboring lattice sites with strength $J_{\mathrm{jk}}$ , with an average strength of J/2π=5.9±0.4MHz . The second term represents the site energies, tuned by the transmon transition frequencies with an accuracy of 300kHz ( $\approx \;5\times 10^{-2}J$ ) in the device (*63*). This system features site-resolved, multiplexed single-shot dispersive qubit readout and enables simultaneous tomographic measurements of the qubit states when combined with single-qubit gates. To prepare superposition states across the energy spectrum of the lattice, the interacting qubits are simultaneously driven via a common control line. The Hamiltonian of the driven lattice is thus the one in Eq. 4. The drive strength Ω can be tuned by varying the amplitude of the applied drive pulse. The common drive couples to each qubit with a complex coefficient $\alpha_{k}$ [see Karamlou *et al.* (*45*) for details]. By changing the drive detuning δ , the distribution of the superposition states across the HCBH energy spectrum can be controlled: With detuning = 0, the superposition state will be concentrated near the center of the energy band, whereas as the magnitude of δ increases, the superposition state approaches the edge of the energy band. At each detuning strength δ , a snapshot is a collection of 4-by-4 projective measurement outcomes of ${\hat{\sigma }}_{j}^{z}$ for each site j . For more details on the data preprocessing of the driven HCBH model, see Supplementary Materials section C1.

The random quantum circuit experiment was conducted on a Google Sycamore processor composed of 70 frequency-tunable transmon qubits with tunable couplers. The quantum processor used has a similar design to Arute *et al*. (*64*) and was carried out on the same processor used in previous works, where typical coherence times, readout errors, and single- and two-qubit gate errors on this particular chip can be found in (*65*, *66*). The two-qubit gates used for this experiment are iSWAP-like gates with an iSWAP angle θ≈0.5π and conditional phase angle ϕ≈0.1π (*66*). We collected data on rectangular subarrays of $N_{q}=20,25,30$ , and 36 qubits (see fig. S11) with variable circuit depths d=4,6,8,10,12,14,16,18 , and 20. For every $N_{q}$ and d , we collected data on 50 different random circuit instances. Each instance contains a different sequence of single-qubit gates randomly chosen from gate set $\left\{\;\sqrt{X^{\pm 1}},\sqrt{Y^{\pm 1}},\sqrt{W^{\pm 1}},\sqrt{V^{\pm 1}}\;\right\}$ , with $W=\left(X+Y\right)/\sqrt{2}$ and $V=\left(X-Y\right)/\sqrt{2}$ . For each of the $N_{c}=50$ random circuit instances, we performed $M_{s}=500,000$ ( $M_{s}=2,000,000$ ) Z-basis measurements for $N_{q}=20,25,30$ ( $N_{q}=36$ ). Thus, $N_{c}\times M_{s}$ bit-strings were collected for each $\left(N_{q},d\right)$ pair.

In Figs. 3 (C and F), we show the linear XEB $F_{\text{XEB}}\left(N_{q},d\right)$ as a function of circuit depth d for different system sizes $N_{q}$ . The XEB is calculated as follows. Parallelized over eight NVIDIA A100 graphics processing units (GPUs), we can exactly simulate random quantum circuits up to $N_{q}=36$ qubits. We use Cirq (*67*) to simulate the same random quantum circuit instances used in the experiment. For each circuit instance, we evolve an all-zero product state $|\;0\rangle^{⊗N_{q}}$ with the circuit to get a state vector $|\psi_{s}\left(N_{q},d\right)\rangle$ , where d∈[4,6,…,20] represents the depth of the circuit, and $s\in \left[1,2,\ldots ,N_{c}\right]$ represents $N_{c}=50$ different circuit instances. To simulate measuring a state in the Z-basis, we sample from the distribution given by ${|\;\psi_{s}\left(N_{q},d\right)\;|}^{2}$ . Similar to the sample size we have in experiments, we draw $M_{s}=500,000$ samples for $N_{q}=20,25,\text{and}\;30$ and $M_{s}=2,000,000$ for $N_{q}=36$ . The linear XEB is defined as

$$\begin{array}{c} F_{\text{XEB}}\left(N_{q},d,s\right)=2^{N_{q}}{\langle p\left(B_{j}\right)\rangle }_{j}-1=2^{N_{q}}\sum_{b\in {\left(0,1\right)}^{⊗N_{q}}}p{\left(b\right)}^{2}-1 \end{array}$$ (10)

where $p\left(B_{j}\right)=|\;\langle \psi_{s}\left(N_{q},d\right){|B_{j}\rangle \;|}^{2}$ . From a finite set of samples, we can get an estimate of $F_{\text{XEB}}$

$$F_{\text{XEB}}\left(N_{q},d,s\right)\approx 2^{N_{q}}\left[\frac{1}{M}\sum_{j=1}^{M}p\left(B_{j}\right)\right]-1$$ (11)

We exactly calculate simulated $F_{\text{XEB}}\left(N_{q},d,s\right)$ using Eq. 10 for each state $|\;\psi_{s}\left(N_{q},d\right)\;\rangle$ and then average over $N_{c}=50$ circuit instances with the same circuit depth to get $\bar{F_{\text{XEB}}}\left(N_{q},d\right)$ in Fig. 3C. The error bar in the plot is the standard error over multiple circuit instances. For experimental XEB (see the main text and Fig. 3E), we estimate $F_{\text{XEB}}\left(N_{q},d,s\right)$ using Eq. 11 for each state $|\;\psi_{s}(N_{q},d)\;\rangle$ and then average over $N_{c}=50$ circuit instances to get estimated $\bar{F_{\text{XEB}}}\left(N_{q},d\right)$ . For more details on the data preprocessing of the random quantum circuit, see Supplementary Materials section D1.

The data of toric code state under coherent and incoherent errors are generated as follows. We start with the Z-basis bit-string measurement from the deformed toric code state with coherent noise, now available in an open-source database (*60*) as a part of Cong *et al*. (*61*). The database includes bit-strings obtained by simulating measuring the toric code state ∣TC〉 deformed by coherent X,Z noise with varying strengths

$$\begin{array}{c} |\psi \left(g_{X},g_{Z}\right)\rangle =\frac{1}{N}e^{-g_{X}\sum_{i}X_{i}-g_{Z}\sum_{i}Z_{i}}|\text{TC}\rangle \end{array}$$ (12)

The bit-strings available in the database are simulated and sampled using the projected entangled pair state on a 300-by-1000 vertex square lattice (*61*). We then introduce incoherent noise through bit-flip with probability $p_{\text{flip}}$ (see the main text and Eq. 6). Thus, the resulting bit-strings are effectively sampled from mixed states $\rho \left(g_{X},g_{Z},p_{\text{flip}}\right)$ . For more details on the data preprocessing of the noisy toric code state, see Supplementary Materials section E1.

### QuAN architecture

The QuAN is a machine learning model designed to learn high-order statistical correlations between quantum measurement snapshots while respecting permutation invariance. It consists of three main components: a convolutional preprocessing module, an encoder based on the MSSAB, and a decoder using a PAB. The architecture is outlined in Fig. 1E, and see Supplementary Materials section A for more details.

#### Input and convolution

The input to the QuAN is a set $X_{i}={\left\{B_{i,\alpha }\right\}}_{\alpha =1}^{N}$ consisting of N binary-valued arrays $B_{i,\alpha }$ , each representing a 2D bit-string of $N_{q}$ qubits. Each element $B_{i,\alpha ,\mu }\in \left\{0,1\right\}$ is indexed by spatial coordinate μ . For clarity, we denote $B_{i,\alpha ,\mu }$ as $B_{i,\mu }^{\alpha }$ . The goal of the model is to predict a label $y\left(X_{i}\right)$ , trained via binary cross-entropy loss

$$L=-\sum_{i}{\hat{y}}_{i}\text{log}\;y\left(X_{i}\right)$$ (13)

where ${\hat{y}}_{i}$ is the ground truth.

To prepare the inputs, we apply a 2D convolutional layer with kernel size 2 and stride 1 to each bit-string, followed by batch normalization and flattening. If the input bit-string is of shape $\left(N_{r},N_{c}\right)$ and $n_{c}$ convolution filters are used, the output dimension per element becomes $d_{x}=n_{c}\left(N_{r}-1\right)\left(N_{c}-1\right)$ . The output of this stage is $x_{\mu }^{\alpha }$ , a matrix of shape $\left(N,d_{x}\right)$.

#### MSSAB encoder

The encoder processes the preprocessed input using the MSSAB, which accesses high-order intersnapshot correlations efficiently.

##### Parallel SAB

The input set is randomly shuffled and partitioned into $N_{s}$ minisets, each of size $N/N_{s}$ . Each miniset is processed independently by a SAB with shared parameters. The input to the encoder is denoted as $x_{\mu }^{\alpha }$ , where α indexes the set element and μ represents the dimension of the feature space after the convolution layer. The SAB transforms the input set to a set of hidden state vectors

$$h_{\mu }^{\alpha }=\sum_{\nu }Q_{\mathrm{\mu v}}x_{\mu }^{\alpha }+\sum_{\beta }A^{\alpha \beta }\sum_{\nu }V_{\mu \nu }x_{\nu }^{\beta }$$ (14)

where Q,K,andV are learnable weights of shape $\left(d_{h},d_{x}\right)$ . The attention score matrix $A^{\alpha \beta }$ is given by

$$A^{\alpha \beta }=\text{Softmax}\left[\sum_{\rho \lambda \eta }\frac{1}{\sqrt{d_{h}}}Q_{\rho \lambda }x_{\lambda }^{\alpha }K_{\rho \eta }x_{\eta }^{\beta }\right]$$ (15)

The output undergoes residual connection, layer normalization, and feed-forward transformation

$$\begin{array}{c} h_{\mu }^{\prime \alpha }=\text{LayerNorm}\left(h_{\mu }^{\alpha }\right)\\ y_{\mu }^{\alpha }=\text{Sigmoid}\left(\text{LayerNorm}\left(h_{\mu }^{\prime }+{\text{FF}}_{\mu \nu }\left(h_{\nu }^{\prime }\right)\right)\right) \end{array}$$ (16)

where FF(x)=ReLU(Ox) or Sigmoid(Ox) for learned weight matrix x.

##### Recurrent AB (RecAB)

Each miniset output $y_{\left(m\right)}$ undergoes attention with other $N_{s}-1$ minisets using multihead attention blocks (MABs)

$$h_{\left(t+1\right),\mu }^{\alpha }=\sum_{\nu }Q_{\mu \nu }^{\prime }y_{\left[\left(m+t+1\right)\text{mod}N_{s}\right],\nu }^{\alpha }$$ (17)

$$\begin{array}{c} +\sum_{\beta }\text{Softmax}\left\{\;[\sum_{\rho \lambda \eta }\frac{1}{\sqrt{d_{h}}}Q_{\rho \lambda }^{\prime }y_{\left[\left(m+t+1\right)\text{mod}N_{s}\right]}^{\alpha }K_{\rho \eta }^{\prime }h_{\left(t\right),\eta }^{\beta }]\;\right\} \end{array}$$ (18)

$$\left.\times \sum_{\nu }V_{\mu \nu }^{\prime }h_{\left(t\right),\nu }^{\beta }\right\}$$ (19)

where $h_{\left(0\right)}\equiv y_{\left(m\right)}$ , and $h_{\left(N_{S}-1\right)}\equiv y_{\left(m\right)}^{\prime }$ . This enables capturing up to $2N_{s}$-th order moments in the input.

##### Reducing AB (RedAB)

To merge $N_{s}$ minisets into one while preserving permutation invariance, RedAB performs $N_{s}-1$ recurrent MAB operations in a randomized order

$$h_{\left(t+1\right)}^{\prime }=\text{MAB}\left\{h_{\left(t\right)}^{\prime },y_{\left[\sigma \left(t+1\right)\right]}^{\prime }\right\}$$ (20)

resulting in a final output $z_{\mu }^{\alpha }$ of size $\left(N/N_{s},d_{h}\right)$ . The MSSAB with one layer accesses up to $2N_{s}^{2}$-th moments. For further details on moment order and computational complexity, see Supplementary Materials section A3d.

#### PAB decoder

The decoder compresses the encoder output $z_{\mu }^{\alpha }$ into a scalar prediction y(X) . The PAB uses a seed vector S and computes attention scores ${s^{\prime }}^{\beta }$ over set elements

$$p_{\mu }=S_{\mu }+\sum_{\beta }s^{\prime \beta }\sum_{\nu }V_{\mathrm{\mu v}}^{\prime \prime }z_{\nu }^{\beta }$$ (21)

where the pooling attention score is given by

$$s^{\prime \beta }=\text{Softmax}\left[\sum_{\rho \lambda }\frac{1}{\sqrt{d_{h}}}S_{\rho }K_{\rho \lambda }^{\prime \prime }z_{\lambda }^{\beta }\right]$$ (22)

After layer normalization, a final linear transformation produces the output

$$p_{\mu }^{\prime }=\text{LayerNorm}\left(p_{\mu }\right)$$ (23)

$$y\left(X\right)=\text{Sigmoid}\left(\sum_{\mu }W_{\mu }\text{LayerNorm}\left(p_{\mu }^{\prime }+{\mathrm{rFF}}_{\mu \lambda }\left(p_{\nu }^{\prime }\right)\right)+b\right)$$ (24)

where W , b , $K^{\prime \prime }$ , $V^{\prime \prime }$ , and S are learnable parameters. This design enables the QuAN to learn from measurement data while ensuring permutation invariance and leveraging high-order moment correlations effectively and efficiently.

### Training and testing procedure

All models presented in this work are implemented using PyTorch and trained using the Adam optimizer to minimize binary cross-entropy loss. A key feature across all tasks is the use of set-structured input data, where each data point comprises multiple quantum measurement snapshots. For the task of learning the entanglement transition in the driven HCBH model, we train binary classifiers to distinguish between states with different entanglement scaling laws. Each model is trained independently for a given set size, and the one with the highest validation accuracy is used for testing. The testing region corresponds to unknown intermediate labels; thus, we analyze the phase transition by averaging the machine confidence $y\left(X_{i}\right)$ over multiple testing sets, and 10 independent models are trained to see good convergence in model performance. We train each model using one A100 GPU for 500 epochs, with a total wall-clock time of ~150 s (see Supplementary Materials section C2 for detailed hyperparameter settings). For learning the complexity between random circuit states, we classify measurement outcomes by circuit depth using depth *d* = 20 as a reference and by varying the shallow depth. We use a fixed set size of *N* = 10,000 and enhance training with a learning rate scheduler and Xavier initialization. Eight independent models are trained for each depth pair (*d*, 20) on simulated data, and performance is evaluated on both simulated and experimental data collected from Google’s Sycamore processor. We train each model using one A100 GPU for 400 epochs, with a total wall-clock time of ~24 hours (see Supplementary Materials section D2 for detailed hyperparameter settings). For learning the decodable and undecodable states of the toric code, we train binary classifiers to distinguish between two states (which also correspond to the topological and trivial phases). Unlike other tasks, convolution layers are omitted to prioritize closed-loop feature learning using MLPs. The resulting phase diagram is derived from the averaged model confidence over testing sets at each parameter point $\left(g_{X},p_{\text{flip}}\right)$ . We train each model using one A100 GPU for 200 epochs, with a total wall-clock time of ~500 s (see Supplementary Materials section E2 for detailed hyperparameter settings).

To ensure robust generalization and avoid overfitting, we adopt a “dataset shuffling period,” where input sets are regenerated or shuffled every 10 epochs. For each model and configuration, we perform multiple independent training runs and report either the average highest validation accuracy or the average machine confidence and its standard error over the trained models. We maintain a consistent classification threshold of y=0.5 for label prediction.

## Supplementary Materials

### This PDF file includes:

Supplementary Text Figs. S1 to S24 Tables S1 to S17 References
