# Supplementary material for: Attention to quantum complexity
Source: Sci Adv. 2025 Oct 10;11(41):eadu0059. doi: 10.1126/sciadv.adu0059 (PMC12513418; doi:10.1126/sciadv.adu0059)
Supplement: Supplementary file 1 — Supplementary Text Figs. S1 to S24 Tables S1 to S17 References [file sciadv.adu0059_sm.pdf]

Science Advances
AAAS

Supplementary Materials for

Attention to quantum complexity

Hyejin Kim *et al.*

Corresponding author: Eun-Ah Kim, eun-ah.kim@cornell.edu

Sci. Adv. **11**, eadu0059 (2025)
DOI: 10.1126/sciadv.adu0059

This PDF file includes:

Supplementary Text
Figs. S1 to S24
Tables S1 to S17
References

Appendix A: QuAN Architecture

Capturing quantum fluctuation and respecting samples' permutation invariance is essential to learn information about quantum states from measurement snapshots. Here, we introduce QuAN, a machine learning model which uses attention mechanism (4) to learn inter-snapshot correlations while respecting the permutation invariance of the snapshots (38,68). Learning high-order moments can be a practical way of learning characteristics of the distribution of bit-strings. However, direct application of existing self-attention blocks (SABs), as in Ref. (38), is inefficient and allows us to access only low-order moments. To study complex quantum systems where high-order moments become crucial, we propose (in Section A 3) a new encoding scheme, named mini-set self-attention block (MSSAB), that can efficiently sample high-order correlations with low computational cost.

In this section, we present details of the inner structure of QuAN. A detailed structure of QuAN architecture is presented in the main text Fig. 1E. The rest of this section is organized as follows: First, in Section A 1, we discuss the input data structure taken by QuAN. Second, we discuss in Section A 2 the usage of a two-dimensional convolution layer and any preprocessing of the raw measurement snapshots before inputting into the encoder. Third, we discuss in Section A 3 how the workhorse in QuAN - the mini-set self-attention block (MSSAB) is used in the encoder to capture inter-snapshot correlations. Lastly, we discuss in Section A 4 the pooling attention block (PAB) in the decoder.

1. Input

QuAN takes a *set* of two-dimensional bit-strings of N_q qubits as input data, where each bit-string consists of N_q binary values (0 or 1). We denote the inputs to QuAN, which are i-th set (datapoint) $\mathbb{X}_i = \{B_{i,\alpha}\}_{\alpha=1}^{N}$ consisting of N two-dimensional binary-valued arrays $B_{i,\alpha}$, where α indexes the elements within the set and N is the set size. (Each binary-valued array has N_q entries indexed by μ, i.e. $B_{i,\alpha,\mu} = 0$ or 1. To avoid confusion between the set element index $\alpha \in \{1, \cdots, N\}$ and the spatial dimension index $\mu \in \{1, \cdots, N_q\}$, we relocate the index α from superscript to subscript, s.t. $B^{\alpha}_{i,\mu} \equiv B_{i,\alpha,\mu}$.) Once $\mathbb{X}_i$ is inputted into QuAN, the output is given by $y(\mathbb{X}_i)$, and QuAN is optimized through binary cross entropy loss $\mathcal{L} = -\sum_i \hat{y}_i \log y(\mathbb{X}_i)$ between true label $\hat{y}_i$ and output.

2. Convolution

The original input data are sets of binary-valued arrays. We first pass the input sets through a convolution layer (see Figure S1). The convolution step has two purposes: First, the original binary-valued arrays are mapped to vectors with continuous entries with better algebraic properties, illustrated in Figure S1. Second, the convolution enables the model to capture possible local spatial features. In the convolution layer, we apply convolution filter $\{F^c\}_{c=1}^{n_c}$ of kernel size $\texttt{kernel} = 2$ and stride 1, on each two-dimensional bit-string array of N_r rows and N_c columns. The channel number n_c is a hyperparameter of the ML model that controls the number of 2×2 filters. After the convolution layer, `BatchNorm2d` over set elements follows. The resulting output is then flattened into a 1D vector. The dimension of 1D vector d_x depends on the convolution layer hyperparameters; $d_x = n_c(N_r - \texttt{kernel} + 1)(N_c - \texttt{kernel} + 1)$. We adopt a stride of 1 without padding throughout this paper. The output of the convolution layer $\mathbf{x}^{\alpha}_{\mu} \equiv \mathbf{x}^{\alpha}_{i,\mu}$ is a matrix of size (N, d_x), where $\alpha = 1, \cdots, N$ and $\mu = 1, \cdots, d_x$.

3. Mini-set Self-attention block (MSSAB) Encoder

The encoder aims to transform its input into a more informative representation, which will be further used in the following decoder block to accomplish the desired task — in our case, the binary classification task. When designing the encoder architecture, we consider two key insights into the data of interest: First, we expect the high-order moments of the bit-string distribution to contain important information. Second, we expect the ordering of the snapshots to be irrelevant since each measurement is drawn independently.

The first insight, the desire to capture high-order moments, motivates us to utilize the attention mechanism. The attention mechanism first introduced in Ref. (4) drives the success of transformers as the core of large language models. The attention mechanism lets the models learn correlations between words (or tokens) in sentences. However, direct usage of the vanilla attention mechanism brings us limited power since the model will view the input bit-strings as a sequence and will try to learn from their ordering.

To overcome this limitation, we must consider the second insight, the permutation invariance of the input bit-strings. Building on the vanilla attention mechanism (4), Ref. (38) introduces a permutation invariant version of transformer, named set transformer, since it treats the input as a set, instead of a sequence. We follow the convention of the set

transformer (38) and call the self-attention module respecting permutation invariance the SAB. Although, in theory, SAB can capture correlations while respecting permutation invariance, a long sequence of stacked SABs is needed for the model to access high-order moments, which soon becomes impractical.

We introduce MSSAB as a parameter-efficient and practical version of SAB when accessing high-order inter-snapshot correlation between bit-strings. While the SAB (38) considers *all-to-all* second-order inter-snapshot correlations between input set elements, our MSSAB *samples* higher-order inter-snapshot correlations and thus greatly reduces the computational cost. A single layer of MSSAB is structured into three key components: a parallel SAB, a recurrent attention block (RecAB), and a reducing attention block (RedAB) (see main text Fig. 1E encoder and Figure S2). Each of these components plays a crucial role in the functionality of MSSAB. We discuss each part in more detail in Sections A 3 a, A 3 b, A 3 c. Finally, we compare the computational complexity of MSSAB with SAB in Section A 3 d.

a. Parallel SAB

First, the input set is shuffled and partitioned into N_s mini-sets, where N_s is the number of mini-sets and is the core hyperparameter of MSSAB. The parallel SAB transforms each mini-set independently. Note that the input set to the encoder is the output of the previous convolution layer.

The parallel SAB block is shown as the purple block in the main text Fig. 1E, and the inner structure in Figure S2(a). We first perform a preprocessing strategy called 'mini-set partitioning,' which is a partition of the input set (set size N) into N_s subsets of size N/N_s, called mini-sets. These mini-sets allow for parallel processing. The main objective of parallel SAB is to capture pairwise second-order correlation only within each mini-set.

The essential unit in parallel SAB is the SAB that allows access to second-order moments between bit-strings, which we will discuss at the end of this paragraph. In the main text, we introduce the simplified version of the self-attention score for a set of snapshots $\mathbb{X}_i$ (see main text Eq. (1)). For actual implementation, we use the output of the convolution layer $\mathbb{x}_i = F^c(\mathbb{X}_i)$ instead of bare snapshots,

$$\langle Q\mathbb{x}_i|K\mathbb{x}_i\rangle = (Q\mathbb{x}_i)(K\mathbb{x}_i)^T \tag{S1}$$

up to normalization. We will omit the index i for convenience. The input to the encoder is denoted as $\mathbb{x}_\mu^\alpha$ where α indexes the set element and μ represents the dimension of the feature space after the previous convolution layer. We will use the Greek letters α, β to represent a set index running from 1 to N and $\mu, \nu, \rho, \lambda, \eta$ for the feature space index running from 1 to d_h (or d_x). SAB transforms the input set to a set of hidden state vectors:

$$\mathbb{h}_\mu^\alpha = \sum_\nu^{d_x} Q_{\mu\nu}\mathbb{x}_\nu^\alpha + \sum_{\beta=1}^{N} \texttt{Softmax}\left[\sum_\rho^{d_h}\sum_{\lambda\eta}^{d_x}\frac{1}{\sqrt{d_h}}\left(Q_{\rho\lambda}\mathbb{x}_\lambda^\alpha K_{\rho\eta}\mathbb{x}_\eta^\beta\right)\right]\sum_\nu^{d_x} V_{\mu\nu}\mathbb{x}_\nu^\beta, \tag{S2}$$

with the hidden dimension size d_h. Q, K and V are query, key, and value matrices of dimensions (d_h, d_x). To gain insight into how the model learns important, relevant features, we rewrite the above expression in the following form:

$$A^{\alpha\beta} = \texttt{Softmax}\left[\sum_{\rho\lambda\eta}\frac{1}{\sqrt{d_h}}\left(Q_{\rho\lambda}\mathbb{x}_\lambda^\alpha K_{\rho\eta}\mathbb{x}_\eta^\beta\right)\right] \sim \texttt{Softmax}\left[\mathbb{x}^\alpha\cdot\mathbb{x}^\beta\right], \tag{S3}$$

$$\mathbb{h}_\mu^\alpha = \sum_\beta A^{\alpha\beta}\left(\sum_\nu Q_{\mu\nu}\mathbb{x}_\nu^\alpha + V_{\mu\nu}\mathbb{x}_j^\beta\right), \tag{S4}$$

where $\sum_\beta A^{\alpha\beta} = 1$. The self-attention score matrix $A^{\alpha\beta}$ is of dimensions (N, N). Calculating the self-attention score involves two set elements ($\mathbb{x}^\alpha$ and $\mathbb{x}^\beta$), which can capture all-to-all second-order moments in $\mathbb{x}$. This is followed by layer normalization on the spatial dimension and a linear layer:

$$\begin{aligned}\mathbb{h}'^\alpha_\mu &= \texttt{LayerNorm}(\mathbb{h}_\mu^\alpha),\\ \mathbb{y}_\mu^\alpha &= \texttt{Sigmoid}\left(\texttt{LayerNorm}(\mathbb{h}'_\mu + \texttt{FF}_{\mu\nu}(\mathbb{h}'_\nu))\right),\end{aligned} \tag{S5}$$

where FF is a feed-forward function for residual connection that acts on each set element equally, which in our implementation is to multiply by a matrix O of dimensions (d_h, d_h) followed by activation function (either Sigmoid or ReLu)), i.e. $\texttt{FF}(\mathbb{x}) = \texttt{ReLu}(O\mathbb{x})$ or $\texttt{Sigmoid}(O\mathbb{x})$ for each set element $\mathbb{x}$. Q, K, V, O are learned weight matrices in SAB. The output of SAB, $\mathbb{y}_i^\alpha$, is a matrix of dimensions (N, d_h).

There are N_s parallel SABs acting on N_s mini-sets, where parameters are shared across all the SAB blocks. We denote the output of parallel SAB as $\{\mathbf{y}_{(0)}, \cdots, \mathbf{y}_{(N_s-1)}\}$, where the subscript with parentheses is the mini-set label. Each output $\mathbf{y}_{(m)}$ is a matrix of size $(N/N_s, d_h)$. Parallel SAB can access second-order moments of a bit-string distribution within each mini-set, unlike ordinary SAB, which accesses all-to-all second-order moments.

b. Recurrent AB (RecAB)

The RecAB takes the outputs of parallel SAB and attends to them recurrently with multiple randomized orderings. A schematic of RecAB is shown as the blue block in Fig. 1E, and the inner structure is plotted in Figure S2(c). RecAB was devised to capture correlations between different mini-sets that were not captured through parallel SAB; by attending mini-sets $\{\mathbf{y}_{(0)}, \cdots, \mathbf{y}_{(N_s-1)}\}$ recurrently, RecAB can capture up to $2N_s$-th order correlation in $\mathbf{x}$.

We utilize a multi-head attention block (MAB) instead of SAB since we compute the attention score between two mini-sets. Each (parallel SAB output) mini-set $\mathbf{y}_{(m)}$ goes into $N_s - 1$ MABs. At each time t attended by the next mini-set $\mathbf{y}_{((m+t+1)\texttt{mod}N_s)}$, where MAB is given by

$$\mathbb{h}^{\alpha}_{(t+1),\mu} = \sum_{\nu}^{d_h} Q'_{\mu\nu}\mathbf{y}^{\alpha}_{((m+t+1)\texttt{mod}N_s),\nu} + \sum_{\beta=1}^{N/N_s} \texttt{Softmax}\left[\sum_{\rho\lambda\eta}^{d_h} \frac{1}{\sqrt{d_h}}\left(Q'_{\rho\lambda}\mathbf{y}^{\alpha}_{((m+t+1)\texttt{mod}N_s),\lambda} K'_{\rho\eta}\mathbb{h}^{\beta}_{(t),\eta}\right)\right] \sum_{\nu}^{d_h} V'_{\mu\nu}\mathbb{h}^{\beta}_{(t),\nu}, \quad \text{(S6)}$$

where $\mathbb{h}_{(0)} \equiv \mathbf{y}_{(m)}$, $\mathbb{h}_{(N_s-1)} \equiv \mathbf{y}'_{(m)}$. Attention score between two different mini-sets involves two set elements $\mathbf{y}^{\alpha}_{(m)}$ and $\mathbf{y}^{\beta}_{(m')}$ each from m and m'-th mini-set, hence capturing the second-order correlation between two mini-sets. Each MAB operation is independent and identical, followed by the same layers as in Eq. (S5). We denote the output of RecAB as $\{\mathbf{y}'_{(0)}, \cdots, \mathbf{y}'_{(N_s-1)}\}$. Each output $\mathbf{y}'_{(m)}$ is a matrix of size $(N/N_s, d_h)$. While each mini-set passes through $N_s - 1$ numbers of MAB recurrently, it involves N_s set elements each from different mini-set $(\mathbf{y}^{\alpha_0}_{(m)}, \mathbf{y}^{\alpha_1}_{(m+1)}, \cdots, \mathbf{y}^{\alpha_{N_s-1}}_{((m+N_s-1)\texttt{mod}N_s)})$, which can capture N_s-th order moment in $\mathbf{y}$. In other words, RecAB can access $2N_s$-th order moments in $\mathbf{x}$, considering $\mathbf{y}$ contains second-order moment information of $\mathbf{x}$.

c. Reducing AB (RedAB)

Finally, the RedAB attends mini-sets in a randomized sequence and shrinks N_s mini-sets into one.

The RedAB is shown as the blue dashed block in Fig. 1E, and the inner structure is plotted in Figure S2(d). RedAB is designed to reduce all (RecAB output) mini-sets into a single mini-set while preserving the mini-set permutation invariance. We attend mini-sets $\{\mathbf{y}'_{(0)}, \cdots, \mathbf{y}'_{(N_s-1)}\}$ in a randomized sequence using $N_s - 1$ MABs (each MAB operation is independent and identical, followed by the same layers as in Eq. (S5); we use the same MAB from RecAB.) Similar to RecAB, RedAB with $N_s - 1$ numbers of MAB involves N_s set elements from different mini-set $(\mathbf{y}'^{\beta_0}_{(\sigma(0))}, \mathbf{y}'^{\beta_1}_{(\sigma(1))}, \cdots, \mathbf{y}'^{\beta_{N_s-1}}_{(\sigma(N_s-1))}$ where σ is a randomized permutation function), which captures N_s-th order moment in $\mathbf{y}'$. In other words, RedAB can access $2N_s^2$-th order moments in $\mathbf{x}$. The final output of RedAB is $\mathbf{z} \equiv \mathbf{z}^{\alpha}_{\mu}$, a matrix of dimensions $(N/N_s, d_h)$ with $\alpha = 1, \cdots, N/N_s$ and $\mu = 1, \cdots, d_h$. Therefore, one MSSAB layer reduces the input set size N to N/N_s.

d. Computational complexity of MSSAB

MSSAB is more parameter-efficient when we need to target high-order moments. In this subsection, we discuss the computational complexity of MSSAB and compare it to the SAB (38). Note that MSSAB with no mini-set partitioning ($N_s = 1$) reduces to ordinary SAB.

First, we walk through how the MSSAB collects increasing order of moments through the parallel-SAB, RecSAB, and RedSAB sequence and finally reaches up to $(2N_s^2)$-th moments in its output.

Parallel-SAB - In parallel-SAB block, each mini-set passes through one layer of SAB (see Figure S2(a)). The transformation performed in SAB is shown in Eq. (S2), where two set elements are involved in the calculation of attention score (see Eq. (S3)), so the outputs $\{\mathbf{y}_{(m)}\}_{m=0}^{N_s-1}$ samples up to 2-nd order moments of the input set $\mathbf{x}$. For simplicity, we define $\texttt{Order}(\cdot)$ to be the order of moments $(\cdot)$ can access. Thus, $\texttt{Order}(\mathbf{y}_{(m)}) = 2$ for $\mathbf{y}_{(m)} \in \{\mathbf{y}_{(m)}\}_{m=0}^{N_s-1}$.

RecAB - The RecAB attends each of $\{\mathbf{y}_{(m)}\}_{m=0}^{N_s-1}$ with others, resulting in $\{\mathbf{y}'_{(m)}\}_{m=0}^{N_s-1}$. Calculating the order of moments is more convenient if we consider the recurrent representation, as shown in Figure S2(b). Recursively, we

have

$$\begin{aligned}\texttt{Order}(\mathbb{h}_{(t+1)}) &= \texttt{Order}(\mathbb{h}_{(t)}) + \texttt{Order}(\mathbb{y}_{(t+1)\mathrm{mod}N_s}) &\text{(S7)}\\ &= \texttt{Order}(\mathbb{h}_{(t)}) + 2, &\text{(S8)}\\ \texttt{Order}(\mathbb{h}_{(0)}) &= \texttt{Order}(\mathbb{y}_{(m)}) = 2. &\text{(S9)}\end{aligned}$$

Via recursion unrolling, we have $\texttt{Order}(\mathbb{h}_{N_s-1}) = 2N_s$. As shown in the unrolled flowchart of RecAB (Figure S2(c)), each row represents a different ordering of $\{\mathbb{y}_{(m)}\}_{m=0}^{N_s-1}$ in which the attention mechanism attends. That is, we initialize $\mathbb{h}_{(0)}$ with N_s different mini-sets, each gives an output among $\{\mathbb{y}'_{(m)}\}_{m=0}^{N_s-1}$. Thus,

$$\texttt{Order}(\mathbb{y}'_{(m)}) = \texttt{Order}(\mathbb{h}_{N_s-1}) = 2N_s. \tag{S10}$$

***RedAB* -** RedAB is similar to the RecAB since both use a recurrent module. However, RedAB only samples one order in which the outputs of RecAB $\{\mathbb{y}'_{(m)}\}_{m=0}^{N_s-1}$ get attended. Again, using the recurrent representation, we now have

$$\begin{aligned}\texttt{Order}(\mathbb{h}'_{(t+1)}) &= \texttt{Order}(\mathbb{h}'_{(t)}) + \texttt{Order}(\mathbb{y}'_{(\sigma(t+1))}) &\text{(S11)}\\ &= \texttt{Order}(\mathbb{h}'_{(t)}) + 2N_s, &\text{(S12)}\\ \texttt{Order}(\mathbb{h}'_{(0)}) &= \texttt{Order}(\mathbb{y}_{\sigma(0)}) = 2N_s. &\text{(S13)}\end{aligned}$$

Unrolling the recursive relation, we obtain

$$\texttt{Order}(\mathbb{z}) = \texttt{Order}(\mathbb{h}'_{N_s-1}) = 2N_s^2. \tag{S14}$$

We have shown that a single layer of MSSAB can access moments up to $2N_s^2$-th order. By stacking L layers of MSSAB, we can reach $(2N_s^2)^L$-th moments.

Second, we consider the runtime complexity of the MSSAB versus the SAB. In a SAB, attention scores are computed between each pair of set elements, accessing up to second-order moments. Consequently, the computational complexity scales quadratically with the input set size N, resulting in an $O(N^2)$ complexity. Our MSSAB remains a similar quadratic scaling of complexity $O(N^2)$. Therefore, accessing higher moments with fewer layers makes MSSAB more efficient than SAB.

To reach moments of order Θ, one need $L = \lceil \log_{2N_s^2} \Theta \rceil$ layers of MSSAB. In contrast, reaching the same order needs $\lceil \log_2 \Theta \rceil$. We show in Figure S3 a scaling of the number of layers required for SAB or MSSAB to reach moments of desired order Θ. Clearly, to reach a larger Θ, the number of layers needed for SAB grows much faster than MSSAB. With $L = 1$ layer of MSSAB with $N_s = 5$, we can access $\Theta = 50$, marked by a red star in Figure S3. In contrast, we need $L = 6$ SAB layers to reach the same order $\Theta = 50$ with SAB.

4. Pooling Attention Block (PAB) Decoder

While the MSSAB encoder handles various orders of moments between bit-strings, the principal operation of the decoder is to pool out useful information from the encoder output while respecting the permutation invariance of the bit-strings. Unlike ordinary pooling operations (such as averaging/summing over snapshots), the pooling attention block (PAB) enables importance sampling by assigning weights through the pooling attention score.

The decoder of QuAN consists of one layer of a pooling attention block (PAB) and a final single-layer perceptron to output a single scalar value, which is the label prediction confidence. The output of the encoder $\mathbb{z}_\mu^\alpha$ is fed into the decoder as its input. Note that, so far, the set dimension, indexed by α, has the dimensionality as the original input $\mathbb{x}$ divided by N_s. In the decoder, the PAB performs the essential operation, which weighs different set elements differently so that elements with important features contribute more to the final result. Writing explicitly, the PAB first transforms the encoder output as

$$\begin{aligned}\mathbb{p}_\mu &= S_\mu + \sum_{\beta=1}^{N/N_s} \texttt{Softmax}\left[\sum_{\rho\lambda}^{d_h} \frac{1}{\sqrt{d_h}}\left(S_\rho K''_{\rho\lambda}\mathbb{z}_\lambda^\beta\right)\right]\sum_\nu^{d_h} V''_{\mu\nu}\mathbb{z}_\nu^\beta\\ &= \sum_\beta s'^\beta\left(S_\mu + \sum_\nu V''_{\mu\nu}\mathbb{z}_\nu^\beta\right),\end{aligned} \tag{S15}$$

where a seed vector S is used as query vector of size $(1, d_h)$ for a weighted average $\mathbb{z}^\beta$ over the set dimension, and K'', V'' are key and value matrices of dimensions (d_h, d_h). This operation plays an important role in making the output $y(\mathbb{X})$ set permutation invariant, which means the output is the same even when shuffling the set elements. Moreover, the pooling attention score s'^β can be considered as a weight of $\mathbb{z}^\beta$; therefore, $\mathbb{p}$ can be considered as a weighted sum of the encoder output. (See SM section E 4 for a detailed discussion on pooling attention score in identifying topologically ordered quantum states.) Here, the pooling attention score s'^β is a vector of size N:

$$s'^\beta = \texttt{Softmax}\left[\sum_{\rho\lambda} \frac{1}{\sqrt{d_h}} \left(S_\rho K''_{\rho\lambda} \mathbb{z}^\beta_\lambda\right)\right]. \tag{S16}$$

Similar to SAB in the encoder, we perform a layer normalization on the spatial dimension and a linear layer:

$$\mathbb{p}'_\mu = \texttt{LayerNorm}(\mathbb{p}_\mu) \tag{S17}$$

$$y(\mathbb{X}) = \texttt{Sigmoid}\left(\sum_\mu W_\mu \, \texttt{LayerNorm}\left[\mathbb{p}'_\mu + \texttt{rFF}_{\mu\nu}(\mathbb{p}'_\nu)\right] + b\right) \tag{S18}$$

where W is a matrix of dimensions $(1, d_h)$ that converts the vector $\mathbb{p}'_\mu$ into scalar output y, which is the confidence of predicting given set $\mathbb{X}$ into one of the classes (e.g. volume-law phase, deep circuit outcome or topological phase for the cases in the main text). S, K'', V'', W, b are learnable parameters in the decoder.

Appendix B: Comparison of QuAN and other ML architectures

This section provides details of all the ML architectures compared with QuAN in Fig. 1E. In particular, we highlight the main differences between these architectures and the QuAN architecture we propose in this work. The models maintain approximately the same total number of trainable parameters to make a controlled comparison between different architectures.

a. ***Multi-layer perceptron (MLP) -*** The multi-layer perceptron (MLP) model contains 4 layers of perceptrons in the encoder, followed by the decoder block composed of one linear layer and another perception (see Figure S4). The first linear layer in the decoder transforms each set element independently, while the second linear layer combines information from different set elements. Note that the MLP architecture does not use the attention mechanism and also does not respect set element permutation invariance.

b. ***Convolutional neural network (CNN) -*** The convolutional neural network (CNN) architecture (see Figure S5) treats the input data as a 3-dimensional object. Hence, it does not respect the permutation invariance of set elements either. The input 3D array goes over 3 layers of normalized 3D convolution that extracts relevant features into n_c channels, and the subsequent linear layer and sigmoid function outputs a binary classification probability.

c. ***Transformer (Transf.) -*** The transformer attends to spatial correlation within one measurement outcome. In contrast to all the other models, the transformer takes one bit-string as an input at a time, considering each bit (0 or 1) as a token. The bit-string goes through a 2D positional encoding followed by ordinary SAB, a linear layer, and an activation function, as shown in Figure S6. Compared to ordinary transformers, our *Transf.* lacks a decoder part, i.e., this model is analogous to an ordinary transformer encoder.

d. ***Set multi-layer perceptron (SMLP) -*** The set multi-layer perceptron (SMLP) is similar to the abovementioned MLP architecture. The main difference between MLP and SMLP lies in the decoder, shown in Figure S7. For SMLP, we modify the decoder such that the model respects the set element permutation invariance. SMLP only contains linear layers, summation pooling, and final non-linear activation functions. The attention mechanism is absent in this model.

e. ***Pooling attention block (PAB) -*** In PAB architecture shown in Figure S8., we replace the encoder of QuAN with two MLP layers. The decoder still contains the PAB block, which utilizes the attention mechanism.

Appendix C: Driven Hard-core Bose-Hubbard model

1. Data preprocessing

In this section, we provide further details on learning the entanglement transition in the driven hard-core Bose-Hubbard model.

The states $\rho = \rho(\delta, t)$ accessible in the emulation are parameterized by the driving detuning parameter δ and the driving time t. For each given state $\rho(\delta, t)$, we acquire a corresponding probability distribution $p(b) = \mathrm{Tr}(\rho|b\rangle\langle b|)$ by experimentally measuring the state 10^4 times in the Z-basis. We then sample snapshots from each state's probability $p(b)$ to generate training and testing datasets. Given that the increase in detuning strength δ correlates directly with the total particle number, we selectively remove snapshots featuring a total particle number other than $n = 8$. Such an operation prevents the ML model from learning the trivial feature – the particle density. The resulting number of snapshots we keep for each state is $M_s = 4096$, each having $n = 8$ total particle number. We emulate 9 different values of δ/J, namely $\delta/J \in \{-2, -1.5, \cdots, 0, \cdots, 1.5, 2\}$. For each fixed value of δ/J, we take $N_c = 17$ grid points along $t \in \{1.4 \times 10^{-7}, 1.5 \times 10^{-7}, \cdots, 2.9 \times 10^{-7}, 3 \times 10^{-7}\}$ seconds, all of which are in the steady state region. Since we are interested in steady states at different δ, we batch along t. Hence, for each δ we have $M = N_c \times M_s = 17 \times 4096 = 69632$ snapshots. Our device is a 4×4 array of superconducting transmon qubits, so each snapshot also has a 4×4 rectangular geometry with $N_q = 16$ binary values.

We train our model on snapshots taken from the volume-law states at $\delta/J = 0$ and from the area-law states at $\delta/J = \pm2$. For each δ/J, we randomly choose 12 out of 17 different values of t for *training* and keep the remaining 5 for *validation*. We then randomly partition the $M_s = 4096$ snapshots from each state $\rho(\delta, t)$ into sets of size N. Half of the sets from $\delta = \pm2$ are combined and used in training so that the total training data size remains the same for the two classes. To train QuAN as a binary classifier between the two phases, we perform supervised learning by labeling sets from $\rho(\delta/J = 0, t)$ as $\hat{y} = 0$, and $\rho(\delta/J = \pm2, t)$ as $\hat{y} = 1$. The details of the training procedure are discussed in the next section.

After training the model, we test the model on *testing* dataset taken from the intermediate phase region $\delta/J \in \{-1.5, \cdots, 0, \cdots, 1.5\}$. The *testing* sets are generated by the same procedure as the *training* sets, but at different detuning strength δ/J. We obtain average confidence $\bar{y}$ over testing sets in predicting the volume-law phase. For $\delta/J = 0$ and $\delta/J = \pm2$, we present the average confidence for the reserved *validation* sets.

2. Training and testing procedure

We use PyTorch to train and test the model to discriminate volume-law and area-law entanglement scalings. Using the Adam optimization algorithm, we minimize the binary cross-entropy loss function (`torch.nn.BCEloss`) of the true (actual) output and machine-predicted output of the given input set. To prevent the model from overfitting, we employ the "dataset shuffling period." Since the model input is set-structured with multiple measurement outcomes, we shuffle or regenerate the input set-structured dataset every 10 epochs to ensure the model can explore various combinations of measurement outcomes. This scheme is used throughout the paper. Training hyperparameters are listed in Table S1, and parameters of architectures are listed in Table S2. We train different models and set sizes (ranging from $N = 2^0 = 1$ to $N = 2^8 = 256$) independently while keeping the remaining model parameters unchanged. For each architecture, we perform 10 independent training to ensure training stability. We store the model with the highest accuracy on *validation* data. When calculating accuracies, we set the classification criteria (threshold) to be $y = 0.5$; that is, $\mathbb{X}_i$ is classified as volume-law if the machine output is $y(\mathbb{X}_i) > 0.5$ and is classified as area-law otherwise. Comparing the machine-predicted labels for *validation* sets with the expected labels yields the validation accuracy.

We don't know the labels in the intermediate region, where *testing* sets are generated. However, we see the phase transition from the machine confidence $y(\mathbb{X}_i)$. We get averaged confidence by averaging the machine output of confidence $y(\mathbb{X}_i)$ over testing sets. We test 10 independent models and obtain the mean and the standard error of the mean of the machine confidences.

3. Machine learning details

After training, we verify the model's performance and stability using validation accuracy as a function of epoch (see Figure S9). Validation accuracy is determined by the percentage of correctly classified validation data points that are in either volume-law ($\delta/J = 0$) or area-law ($\delta/J = \pm2$) state (see section C 1). For instance, an accuracy of 90%

indicates that the model correctly classifies 90% of the total data. If the accuracy hovers around 50%, it suggests that the model fails to train, as random guessing would yield the same accuracy of 50% for any binary classification task.

From Figure S9(a), we observe the differences in accuracy curves with different set sizes N we use to train QuAN$_4$. When the set size is small (e.g., $N < 8$), test accuracy saturates at a much lower value compared to the larger set size ($N \geq 64$). The saturated accuracy increases as we increase the set size to $N = 64$, suggesting that the model can better classify the data using larger set sizes. From the trend with increasing set size, it is clear that having a set structure for input is essential for QuAN to learn the entanglement transition. However, we observe that accuracy is upper-bounded by increasing the set size beyond $N > 64$. For the two largest set sizes ($N = 128$ and 256), the accuracy is not higher than the saturated accuracy for $N = 64$. Moreover, the variance of learning curves across 10 independent training gets larger with increasing set size N due to decreased total training points. Even the set structure plays an important role; using a large set size is limited by the total number of snapshots.

Figure S9(b) shows the accuracy curves for different architectures. All 4 models require input with a set structure, and we maintain the same set size $N = 64$ for fair comparison. Models with self-attention (QuAN$_2$, QuAN$_4$) exhibit higher accuracy saturation than models without self-attention (PAB, SMLP), which hovers around 60%. Especially, QuAN$_4$ accuracy saturates faster and higher compared to QuAN$_2$, demonstrating that having more SAB layers is advantageous. Another thing to notice is that the range or variance of accuracy across 10 independent training runs (shaded region) varies among architectures. Notably, QuAN$_4$ demonstrates good stability with smaller variance at later epoch$\approx$ 500, compared to other architectures.

Following model validation, we now test the model performance in Figure S10 by examining the averaged confidence $\bar{y}$ averaged over the testing dataset that includes intermediate region $\delta/J \in \{-1.5, -1.0, -0.5, 0.5, 1.0, 1.5\}$ (see also main text Fig. 2E-G in the main text). If the averaged confidence $\bar{y}$ is near 0.5, we consider the model fails to distinguish volume-law and area-law features. In Figure S10(a), we present the average confidence for predicting volume-law, using different set sizes N for our model QuAN$_4$. Notably, as we increase the set size to $N = 64$, the transition prediction becomes sharper, emphasizing the importance of utilizing a set structure. However, for larger set sizes beyond $N > 64$ (i.e., $N = 128$ and 256), the average confidence trend over drive detuning δ/J remains the same with the $N = 64$ training. Moreover, the variance (error bar) of average confidence $\bar{y}$ across 10 independent training increases with set size $N > 64$, which aligns well with the accuracy curve in Figure S9(a). Despite the benefits of large set sizes, there is a trade-off due to a fixed number of snapshots $M = 69632$; by increasing the set size, we sacrifice the total number of sets available. Specifically, we have a total $M/N = 1088$ sets for $N = 64$, $M/N = 544$ sets for $N = 128$ and $M/N = 272$ sets for $N = 256$. Beyond a set size of $N = 64$, we encounter a risk of model overfitting to the training dataset if we don't use sufficient training data points. We conclude that a set size beyond $N = 64$ does not have an advantage in observing the entanglement transition.

In Figure S10(b), we present the entanglement transition witnessed with different architectures while maintaining the same set size $N = 64$. Models with self-attention (QuAN$_2$ and QuAN$_4$) exhibit a sharper distinction between area-law and volume-law phases compared to models without self-attention (PAB, SMLP). Moreover, we observe QuAN$_4$ achieves the smallest error bar (variance across 10 independent training) except for SMLP, which already demonstrates poor performance in observing the entanglement transition. This result again highlights the advantage of QuAN$_4$ that can access high moments between sampled bit-strings.

4. Correlation as a witness of entanglement

A useful witness of the transition between the area-law and volume-law entangled states for the HCBH model is the correlation in the X direction. In fact, it is shown in Ref. (46) that the connected correlation function of a physical observable M between two disjoint regions A and B is upper bounded by the mutual information between these two regions:

$$(\langle M_A M_B \rangle - \langle M_A \rangle \langle M_B \rangle)^2 \leq 2||M_A||^2||M_B||^2 I(A:B). \tag{S19}$$

Here $||M||$ is the norm of the operator M, and the mutual information is defined as

$$I(A:B) \equiv S_A + S_B - S_{AB}, \tag{S20}$$

where S_A is the von Neumann entropy of the reduced density matrix via tracing out the region B from the density matrix ρ_{AB} of the two regions: $\rho_A = \mathrm{Tr}_B(\rho_{AB})$, and similarly for S_B. Therefore, in an area-law entangled state where the mutual information decays exponentially, the correlation function will decay no slower than exponentially. In contrast, for the volume-law entangled state, the entanglement entropy scales with the size of each (sub)system. Therefore, the mutual information between A and B almost vanishes no matter the size or distance of A and B. Hence, the correlation function will also remain small across the distances.

In practice, the choice of the observable M depends on the physical nature of each specific system. A suitable option for the coherent-like state obtained from the HCBH Hamiltonian would be $M = \hat{\sigma}^x$. This is mainly due to the fact that the particle exchange interaction in the Hamiltonian in Eq. (9) in the main text happens between the spin components $\hat{\sigma}^{\pm}$. Indeed, as shown in Fig. 2F,G in Ref. (45), the connected correlation function $\langle \hat{\sigma}_i^x \hat{\sigma}_j^x \rangle - \langle \hat{\sigma}_i^x \rangle \langle \hat{\sigma}_j^x \rangle$ decays exponentially in the area-law entangled phase with a finite correlation length, while deep in the volume-law entangled phase the correlation function regardless of distance.

Appendix D: Random quantum circuit

1. Data preprocessing

Out of $N_c = 50$ circuit instances for each depth, we randomly choose $0.7N_c = 35$ circuit instances as *training* circuits and the remaining 15 as *testing* circuits. For each $|\psi_s(N_q, d)\rangle$, we partition the M_s measurement snapshots sampled from the state into sets of set size N. For $N_q < 36$ and the set size of $N = 10,000$, we have $35 \times M_s/N = 1750$ training sets per depth d, $15 \times M_s/N = 750$ sets for testing each. For $N_q = 36$, we have 7000 training sets and 3000 testing sets. (Note that we have $M = N_c \times M_s = 50 \times 500,000 = 25,000,000$ bit-strings for $N_q < 36$ and $M = N_c \times M_s = 50 \times 2,000,000 = 100,000,000$ bit-strings for $N_q = 36$.) We combine all sets from the 35 (15) different circuit instances with the same circuit depth d and label them as the same class when generating the *training* (*testing*) dataset. We train our models on data from two depths d and 20, labeled by $\hat{y} = 0$ and $\hat{y} = 1$ correspondingly.

As a baseline, we also train the model with shallow and deep depth both at $d = 20$. In this case, we expect the model to fail the classification task and have accuracy at 50%. We randomly choose 35 circuit instances as a training circuit from $d = 20$, partition them into sets, and assign half of the sets to shallow depth (label $\hat{y} = 0$) and the other half to deep depth (label $\hat{y} = 1$). Testing datasets are constructed from the remaining 15 circuit instances in the same way.

2. Training and testing procedure

We use PyTorch to train the models as binary classifiers to distinguish shallow-depth circuit measurement outcomes from deep-depth circuit measurement outcomes. We set the reference deep to be $d = 20$ and vary the shallow depth. We use Adam optimization with binary cross-entropy loss and also utilize the dataset shuffling method, described in section C 2. Detailed model and training hyperparameters are presented in Table S5 and S4. We employ a step learning rate scheduler and Xavier normal initialization to enhance learning further. We use the MSSAB module with $N_s = 5$ to deal with a large set size $N = 10,000$. We keep the model with the highest test accuracy during each training run. The training curves are shown in Figure S13.

For the results presented in the main text, we perform 8 independent training runs on noiseless simulated data for each depth pair $(d, 20)$, where $d \in \{4, 6, \cdots, 20\}$. Therefore, for each $(d, 20)$, we have 8 models. We then test the trained models with simulated testing data from the same depth pair $(d, 20)$. Note that this is different from the hard-core Bose-Hubbard model (section C 2) and toric code (section E 2) studies, where we validate models trained on specific training parameter points across the entire phase space. We then calculate the mean and the standard error of the mean of the accuracy from the 8 independent models for each $(d, 20)$, as presented in the main text Fig. 3D. We then tested the experimental data generated with Google's Sycamore processor. For each $(d, 20)$, we apply the 8 models trained on simulated data to classify experimental data at the same depth pair (see main text Fig. 3G). Note that we use experimental data from the same circuit instances used in simulation.

3. Relative cross-entropy benchmarking (XEB) as a depth-inference baseline

In this subsection, we benchmark QuAN's depth inference accuracy directly with an information-theoretic metric based on XEB. Recall that the linear XEB for a collection of bit-strings $\{B_j\}$ sampled form a random circuit instance indexed s with system size N_q and depth d is defined as

$$\mathcal{F}_{\text{XEB}}(N_q, d, s) = 2^{N_q} \langle p(B_j) \rangle_j - 1 \tag{S21}$$

where $p(B_j) = |\langle \psi_s(N_q, d)|B_j\rangle|^2$ is the probability of sampling bit-string B_j from the ideal pure state $|\psi_s(N_q, d)\rangle$ produced by the random unitary circuit U_s of depth d and system size N_q. To enable a direct comparison between QuAN and XEB as depth-inference metrics, we define the relative XEB as

$$\Delta\overline{\mathcal{F}_{\text{XEB}}}(d|N_q) \equiv \frac{1}{2}\left(\frac{\overline{\mathcal{F}_{\text{XEB}}}(N_q, d) - \overline{\mathcal{F}_{\text{XEB}}}(N_q, 20)}{\max_d \overline{\mathcal{F}_{\text{XEB}}}(N_q, d) - \min_d \overline{\mathcal{F}_{\text{XEB}}}(N_q, d)}\right) + \frac{1}{2}, \tag{S22}$$

where $\overline{\mathcal{F}_{\text{XEB}}}(N_q, d) = \frac{1}{N_c}\sum_{s=1}^{N_c} \mathcal{F}_{\text{XEB}}(N_q, d, s)$. Note that the relative XEB ranges from 0.5 to 1, similar to QuAN's accuracy.

As shown in Figure S12(b), QuAN maintains high depth distinguishability up to $d \approx 10$, with accuracy converging to the random baseline (50%). In contrast, the relative XEB saturates near $d \approx 8$ (Figure S12(a)), becoming insensitive

to further increases in depth. This demonstrates that QuAN can extract depth-dependent features beyond the regime where XEB ceases to provide useful discrimination. For experimental bit-strings, the relative XEB (see Figure S12(c)) smoothly decays toward 0 with increasing depth, reflecting the cumulative effect of noise which dries the state toward a maximally mixed state (see also main text Fig. 3F). In this regime, relative XEB fails to capture the growth of complexity that originates from the unitary circuit evolution, instead reflecting noise in the state. In contrast, QuAN trained on pure-state data retains transition near $d \approx 10$, which is consistent with the behavior observed in pure-state data (see Figure S12(d)). This indicates that QuAN can extract the signature of pure-state complexity growth from experimental data even when the standard information-theoretic metric fails.

4. Machine learning details

In Figure S13, we show the learning curves to check the model's performance and stability using test accuracy and loss curve as a function of epoch. We use simulated data from depth pair $(8, 20)$ for $N_q = 25$. The test accuracy is determined by the percentage of correctly classified test data from depths 8 and 20. Test loss is calculated through binary cross-entropy between input true label $\hat{y} = 0, 1$ and model confidence $y(\mathbb{X})$ (see section A 1). The loss function quantifies how much the machine's confidence deviates from the true label. The increasing test loss over epochs signals overfitting to the training dataset and poor generalization to the testing dataset. As previously discussed in the main text Fig. 3E, only QuAN_2 and QuAN_{50} distinguish depth 8 and 20, while other architectures (MLP, CNN, Transf., SMLP, PAB) have accuracies fluctuating near 50%. Testing loss curves for these models diverge or stay constant, implying the model fails to distinguish between depth 8 and 20 data. Now, if we look at the accuracy curves for QuAN_2 and QuAN_{50}, we observe both accuracy curves stay higher than 50% up to epoch= 400 (see Figure S13(a)). However, the testing loss for QuAN_2 is increasing, signaling overfitting of the training dataset, while QuAN_{50} shows a decreasing trend in the loss function (see Figure S13(b)). Moreover, QuAN_{50} accuracy saturates to a higher value at an earlier epoch compared to QuAN_2 and also shows smaller variance across 5 independent training runs (shaded region). This demonstrates QuAN_{50} has good stability with less dependence on randomness (model initialization, selection of training and testing circuit index, random seeds). We conclude QuAN_{50} exhibits stable and high performance. In the remainder of this section, we exclusively employ QuAN_{50} for random quantum circuit depth classification tasks.

We show in Figure S14 the effect of varying set size N to demonstrate the importance of set structure again. We trained QuAN_{50} with the simulated data from depth pair $(8, 20)$ for $N_q = 25$ with varying set size $N = 80, 400, 2000$ and 10000. Validating the trained QuAN_{50} models with test sets of the same set size as training sets, we show the test accuracy as a function of set size N (Figure S14). Test classification accuracy increases with increasing set size, demonstrating the importance of set structure in observing the relative complexity difference between shallow and deep circuit data.

In Figure S15, we conduct a hyperparameter study for QuAN_{50} using the same data (depth 8 and 20 with $N_q = 25$ system with $N = 10000$, see Table S5). The hyperparameters include a number of mini-sets N_s, the number of convolution channels n_c, the hidden dimension size d_h, and the number of heads n_h inside each attention block. As a baseline, we use $(N_s, n_c, d_h, n_h) = (5, 16, 16, 4)$ and tune one type of hyperparameter at a time. We record the highest testing accuracy for each training. The plots show slight changes in average accuracy with varying hyperparameters. Therefore, we conclude that our hyperparameter choice of $(N_s, n_c, d_h, n_h) = (5, 16, 16, 4)$ yields optimal performance given current computational resource. (For extended model ablation study regarding accessible moment order with varying mini-set size N_s, see SM section D5.)

In Figure S16, we provide a more detailed analysis of experimental data by comparing the classification accuracy curve for simulation and experimental data. Using the model QuAN_{50} with optimal hyperparameter setting, we train the model using the data from different (N_q, d) to learn the evolution of state complexity in random circuits as a function of depth d. Our approach involves a multiple pairwise classification task, comparing data from depth d to that from depth 20. The first classification task uses simulated data without noise, whose result is shown in main text Fig. 3D and Figure S16(a). Next, we move on to classification using experimental data with noise, shown in main text Fig. 3G and Figure S16(b). To inspect experimental data with QuAN, we utilize a model trained using depth d and 20 simulated data and then calculate test classification accuracy between depth d and 20 experimental data. In the main text, we highlighted that the classification accuracy for experimental data shows a similar trend to the simulated data, exhibiting a sharp transition at depth 10. However, an exception occurs for system size $N_q = 36$, where the accuracy trend differs in distinguishing depth 4 and 20. To see whether a qualitative difference exists for $(N_q, d) = (36, 4)$, we employ a new scheme of training and testing both on the experimental data while keeping all other hyperparameter settings the same (see Figure S16(c)). We observe two key features from these curves. Firstly, QuAN readily distinguishes experimental data from depth d and depth 20. This likely reflects the increasing degree of noise that comes with circuit depth. Secondly, higher classification accuracy is seen on $N_q = 36$ data, taken on a different day from the rest $(N_q = 20, 25, 30.)$

To understand how QuAN's performance varies with larger system sizes, we study the scaling of its classification accuracy with respect to system sizes (Figure S17). We focus on distinguishing $d = 8$ and 20, which are the limits by which QuAN can differentiate between the two circuit depths. Additionally, we test on noiseless simulated bit-strings to ensure that the scaling is not influenced by noise or potential noise level dependencies with system sizes. Figure S17 presents the scaling of QuAN$_{50}$'s testing accuracy with different system sizes. We examine two different training setups:

1. We train QuAN using a simulated dataset that matches the size of the experimental data. Specifically, $M_s = 5 \times 10^5$ for $N_q = 20, 25, 30$ and $M_s = 2 \times 10^6$ for $N_q = 36$. The results are shown in the black curve, which re-plots the accuracy values from the main text, Fig. 3D, with fixed $d = 8$ at different system sizes N_q.

2. We generate additional simulation data for $N_q = 20, 25, 30$ so that all four system sizes have the same data volume, $M_s = 2 \times 10^6$. We then train QuAN$_{50}$ with 70% of the enlarged simulated datasets and show the testing accuracy in red. We train five independent models for each system size to obtain the average and standard error.

The two curves obtained from the slightly different training setups are consistent. Furthermore, in both setups, the accuracies exhibit sub-exponential scaling with respect to the system size.

5. Circuit depth and moments of the bit-string distribution

Although we leave it to future work to understand precisely which features of the bit-string distribution are used by QuAN to classify circuit depth, we can motivate the structure of QuAN by considering some general features of random local circuits. Specifically, permutation invariance and access to higher moments of the bit-string distribution are natural requirements of the depth classification problem.

A standard definition of the exact circuit complexity for a unitary $U \in U(2^n)$ acting on n qubits is the minimal depth (perhaps from a fixed gate set) needed to implement U exactly. In practice, we might be more interested in the approximate circuit complexity, defined analogously: given some operator norm $\|\cdot\|$, we look for the minimal depth circuit $U_{\mathrm{circ}}(U)$ such that $\|U_{\mathrm{circ}}(U) - U\| < \epsilon$. For random local circuits, it has been shown that the exact circuit complexity grows linearly in depth up to depths exponential in the system size (16). Rigorous results for approximate circuit complexity are weaker (37), although it is also conjectured to grow linearly with depth (7,36). A fundamental question is how much can be learned about the complexity of a given unitary evolution from the experiment.

More specifically, we apply U to the initial state $|0\rangle$. The outcomes of terminal measurements in the z-basis are bit-strings $\{b_j\}$, $j \in \{1, \ldots, n\}$, with probability

$$p_U(\{b_j\}) = \mathrm{Tr}\left(|0\rangle\langle 0| U^\dagger \prod_j \frac{1 + (-1)^{b_j} z_j}{2} U \right). \tag{S23}$$

We have written (S23) in a way that emphasizes that this quantity is linear in the operator $U \otimes U^\dagger$, and kth-order products are linear in $U^{\otimes^k} \otimes (U^\dagger)^{\otimes^k}$. For our random circuits, the probabilities for a finite subset (not scaling with system size) of the bits equilibrate to those for infinite depth in constant depth. At the same time, there are typically an exponential number of typical bit-strings at moderate depth. This suggests that any classifier of depth would require access to additional information.

In our case, QuAN can access higher moments of the probability distribution of the bit-strings. Intuitively, as the circuit depth grows, the distribution of generated unitaries approaches the uniform (Haar) distribution over $U(2^n)$. This approach is quantified by the convergence of increasingly higher moments of the matrix entries. Explicitly, for any measure μ we define the operator $M_\mu^{(k)} = \int \mathrm{d}\mu(U) U^{\otimes^k} \otimes (U^\dagger)^{\otimes^k}$. When the distance $\|M_\mu^{(k)} - M_{\mu_{\mathrm{Haar}}}^{(k)}\| < \epsilon$, we say that μ is an ϵ-approximate k-design. The moments $M_\mu^{(k)}$ can equilibrate (at fixed precision) to the corresponding Haar moments at different depths (the relationship between k and depth is conjectured to be linear by (36), bounded by (37)), and conversely the property of being an approximate k-design implies lower bounds on the complexity (15). The average kth order moment of the distribution of some subsets of bit-strings is just a particular matrix element of $M_\mu^{(k)}$ (see Eq. (S23)), showing that higher moments can in principle give access to depth information not available from lower ones. The XEB is an example for $k = 2$. We leave it to future work to understand what aspects of the higher moments are learned by QuAN.

6. Effect of maximal accessible moment on QuAN's performance

In this subsection, we explore practical limits on the order of moments accessed by QuAN of fixed hidden dimension applied to a fixed sample size. We vary the number of mini-sets N_s in our mini-set self-attention block (MSSAB), which in principle allows QuAN to access moments up to $2N_s^2$. In this study, the total number of trainable parameters was kept fixed in order to study the practical limits. The number of mini-sets is chosen to be an integer divisor of the total set size N (e.g., $N_s = 1, 2, 4, 5, 10, 20$ for $N = 10,000$. We train QuAN to classify quantum state measurement data generated at depths $d = 8$ and $d = 20$ with system size $N_q = 25$. For each training, we take the highest test classification accuracy. We conducted 8 independent training and report the mean value and standard error of the accuracies of the trained models.

As shown in Figure S18, QuAN's performance first increases with increasing N_s until it peaks and then eventually drops. The initial increase implies effectively accessible order of moments increase with increasing N_s and improves QuAN's learning of relative complexity (see the discussion around main text Fig. 3E). However, increasing N_s with fixed set size N invariably limits statistical resolution for estimating moments because the number of mini-sets shrinks. Moreover, increasing N_s deepens the network within the MSSAB unit, thereby increasing training difficulty even for a fixed number of parameters. The results shown in Figure S18 confirm this expectation.

Appendix E: Toric code simulation

1. Data preprocessing

We construct training and testing datasets by transforming the Z-basis measurements of the simulated mixed-state toric code into dual lattice sites with dimensions of 300×1000 Z-plaquette terms. We extract $M_s = 8134$ snapshots for each state by slicing 300×1000 dual lattice sites into 6×6 arrays, each containing 84 qubits. These snapshots represent different quantum states, denoted as $\{\rho_s(g_X, g_Z, p_{\text{flip}})\}$, where s is index for different state. The number of distinct states corresponding to a given parameter set $(g_X, g_Z, p_{\text{flip}})$ ranges from 1 to 13. In our analysis, we fix $g_Z = 0.14$.

We create *training* and *validation* datasets at various bit-flip probability p_{flip}, in both the topological phase ($p_{flip} \in \{0, 0.005, 0.01, 0.015, 0.02\}$) and trivial phase ($p_{\text{flip}} \in \{0.3, 0.305, 0.31, 0.315, 0.32\}$). To ensure an adequate number of data points for *training*, we use different coherent noise points ($g_Z = 0.14$ and $g_X \in \{0, 0.02, 0.04, 0.06, 0.08\}$) as topological or trivial phase points (see hatched boxes in main text Fig. 4C,D). Importantly, we strictly sample from the region where $g_X < g_c \approx 0.22$. We have a total of $N_c = 200$ distinct states, resulting in $M = N_c \times M_s = 200 \times 8134$ snapshots per phase. Out of the 200 states, we randomly allocate $0.75N_c = 150$ states (75%) for *training* and the remaining 50 for *validation*. For a given state $\rho_s(g_X, p_{\text{flip}})$ with $M_s = 8134$ snapshots, we create a set $\mathbb{X}_i$ of set size N by partitioning snapshots into $\lfloor M_s/N \rfloor = 127$ sets. Each snapshot within this set is composed of the same state $\{\rho_s(g_X, p_{\text{flip}})\}$. For instance, we have $150 \times \lfloor M_s/N \rfloor = 19050$ training sets, and $50 \times \lfloor M_s/N \rfloor = 6350$ *validation* sets per phase for a set size of $N = 64$.

After training the model, we test the model using *testing* dataset taken from the entire phase space of coherent noise $0 \le g_X \le 0.38$ and incoherent noise $0 \le p_{\text{flip}} \le 0.32$, which includes an intermediate region (see Figure S20). For the points inside the training region, we randomly choose one state from the *validation* states. For the points outside of the training region, we randomly choose one state from $\{\rho_s(g_X, p_{\text{flip}})\}$. Consequently, for the set size of $N = 64$, we use $\lfloor M_s/N \rfloor = 127$ *testing* sets per each point (g_X, p_{flip}).

2. Training and testing procedure

We use PyTorch to train and test the model to distinguish the topological and trivial phases. We again minimize the binary cross entropy loss function using Adam optimization, step learning rate scheduler, and dataset shuffling method (see SM section C 2) to prevent the model from overfitting. Training hyperparameters are listed in Table S3, and parameters of architectures are listed in Table S6. We train three different architectures (ranging from SMLP to QuAN$_2$) and different set sizes (ranging from $N = 1$ to $N = 64$) independently while keeping the remaining model parameters unchanged. For each architecture, We also perform 10 independent training to ensure stability. As listed in Table S6, we no longer use the convolution layer that inspects spatial correlation inside each snapshot. Instead, we make use of MLP as a function to deal with nonlinear correlations between closed loops within the snapshot.

During the training process, we store the model with the highest validation accuracy. This model is later used to test the dataset. We obtain a phase diagram from the machine confidence $y(\mathbb{X}_i)$; we average the machine output of confidence $y(\mathbb{X}_i)$ over 127 testing sets for each point (g_X, p_{flip}). We calculate the average confidence for each of the 10 independently trained models and obtain the mean and the standard error for the average confidence.

3. Benchmarking machine results

a. *Robustness of phase boundary prediction*

To verify the robustness of the phase boundary prediction against a specific choice of training parameter regions, we vary the training points within the trivial phase while keeping the training points in the topological phase fixed. Specifically, we train QuAN$_2$ using nearly coherent data at $p_{\text{flip}} = 0, 0.005, 0.01, 0.015, 0.2$ across a range of $g_X = 0, 0.02, 0.04, 0.06, 0.08$ (marked as green box in Figure S19(a,b)), and deeply incoherent data over the same range of g_X, while varying p_{flip} (pink box in Figure S19(a,b)). We compare QuAN's output confidence over the phase space for different incoherent training points: $p_{\text{flip}} = 0.3, 0.305, 0.31, 0.315, 0.32$ and $p_{\text{flip}} = 0.45, 0.455, 0.46, 0.465, 0.47$. As shown in Figure S19, the predicted phase boundary for the two trainings are ($p_c = 0.115 \pm 0.004, g_c = 0.251 \pm 0.004$) and ($p_c = 0.111 \pm 0.004, g_c = 0.249 \pm 0.004$) along incoherent and coherent noise level axis correspondingly. The QuAN-predicted phase boundaries remain consistent despite the severe shifts in the incoherent training region, which demonstrates the robustness of QuAN's learning regardless of the choice of training regions.

b. Benchmarking to locally error-corrected decoration (LED)

Here, we present the extended results from QuAN trained with toric code data through phase diagrams of the topologically non-trivial and trivial state, which can be constructed from average confidence $\bar{y}(g_X, p_{\rm flip})$ as a function of coherent noise g_X and incoherent bitflip noise $p_{\rm flip}$ (see Figure S20(c-d)). For comparison, we present the phase diagram obtained using "locally error-corrected decoration" (LED) (see Figure S20(a-b), reproduced from Ref. (61)), which classifies the topological phase based on the vanishing Z-loop tension α after layers of operation. Here, the loop tension α is defined as $\langle Z_{\rm loop}(\gamma)\rangle = e^{-\alpha|\gamma|}$ for a loop γ. As discussed in the main text, we observe that QuAN$_2$ sharpens the transition and even saturates the known threshold $p_c \approx 0.11$ with increasing set size N.

We quantify sample complexity as another method to benchmark model performance against LED, shown in Figure S21. In our context, sample complexity is defined as the number of samples (snapshots) required to confirm that the state is in the topological phase with 95% confidence. We aim to evaluate sample complexity as a function of $p_{\rm flip}$ along $g_X = 0$ and see how the required number of samples grows as we increase the incoherent noise level. We utilize 13 different states for each $(g_X, p_{\rm flip}) = (0, p_{\rm flip})$ where $0.025 \leq p_{\rm flip} \leq 0.3$, having a total $13 \times 127 = 1651$ testing sets per point. To ensure a fair comparison, we exclude points that overlap with the training points.

To determine the sample complexity at a given point, we employ a t-test between the model outputs from each set $y(\mathbb{X}_i)$ and the classification threshold at $y = 0.5$. For each model, we first randomly sample D sets out of 1651 sets from each $p_{\rm flip}$, then feed in to obtain $y(\mathbb{X}_i)$, where i runs from 1 to D. (We randomly feed each set into one stored model out of 10 independently trained models with equal probability.) We conduct the t-test with the null hypothesis that "those D sets are in trivial phase with an average confidence $\bar{y} \leq 0.5$". Suppose the resulting p-value (probability of observing those outputs assuming a trivial phase) is less than 5%. We reject the null hypothesis in that case, indicating that the state is in the topological phase with over 95% confidence. We decrease the number of sets D, and D^* is identified as the point where the prediction fails to meet the 95% confidence level. We define $D^* \times N$ as the sample complexity, where N is the size of each set. This process is repeated 10 times to ensure the stability of our calculation, and the mean and standard error of $D^* \times N \times N_{uc}$ is plotted.

Upon obtaining sample complexities using different set sizes N and architectures (PAB, QuAN$_2$), we compare them with the sample complexity using bare Wilson loop $\langle Z_{\rm closed}\rangle$ without LED (61) as a *baseline*. Here, sample complexity refers to the number of samples required to confirm $\langle Z_{\rm closed}\rangle$ is non-zero with 95% confidence, calculated by $(2\sigma/\langle Z_{\rm closed}\rangle)^2$ assuming a Gaussian distribution of loop expectation values. In Figure S21, we present the sample complexity at various $p_{\rm flip}$ using models with different hyperparameters. For QuAN$_2$ with varying set sizes, we observe that the sample complexity increases exponentially starting at low $p_{\rm flip}$, for small set sizes (e.g., $N = 1$). However, with larger set sizes (e.g., $N = 64$), sample complexity remains relatively unchanged from its minimum value ($D^* \times N \times N_{uc} = 1 \times 64 \times 36 = 2304$) until $p_{\rm flip}$ approaches phase transition. Comparing this to the *baseline* (marked in grey in Figure S21), the advantage of using QuAN$_2$ is clear, especially with a large set size ($N = 64$). Although the *baseline* sample complexity is lower for $p_{\rm flip} < 0.035$, the sample complexity for QuAN$_2$ with $N = 64$ remains constant even until $p_{\rm flip} < 0.09$. We hence conclude that QuAN$_2$ is a scalable method for a broader range of incoherent noise. The sample complexity performance of PAB trends is similar to QuAN$_2$ (see Figure S21(b)), where both QuAN$_2$ and PAB show constant sample complexities on a broader range of incoherent noise. This indicates that PAB operation plays a central role in maintaining a low sample complexity level.

c. Benchmarking to SMLP

Now, we benchmark QuAN and PAB training to the simplest set-structured model, SMLP (see Figure S22). To see how the characterization of the topological phase changes as we tune the architecture complexity and set size, we train three different architectures (SMLP, PAB, QuAN$_2$, see Table S6) with varying set sizes. In the main text Fig. 4E-H, we made a comparison between QuAN$_2$ of various set sizes with SMLP($N = 64$) and PAB($N = 64$). Here, we would also like to show the effects of set sizes on SMLP and PAB and make a comprehensive comparison with QuAN. We draw the following two important conclusions from Figure S22. First, for both $g_X = 0$ and $p_{\rm flip}$ axis, we find that SMLP prediction remains unchanged with increasing set size and even introduces a larger error bar due to a decrease in total training data points. In contrast, PAB/QuAN sharpens the phase boundary with increasing set size (see also main text Fig. 4E-H for QuAN results). This implies that even with a set structure, treating every snapshot equally by averaging over snapshots does not help to predict the topological phase. Comparison of the performances between PAB and QuAN$_2$ leads us to a crucial conclusion: the PAB module plays a pivotal role in characterizing topological order. In the next section E 4, we will make a comprehensive analysis of the PAB module, providing a deep understanding of its role in mixed-state toric code.

4. Machine analysis: PAB as an importance-sampler

Here, we analyze the PAB module in more detail (see Fig. 4I,J in the main text) and discuss the mechanism of the PAB in predicting the topological phase. According to Eq. ((S15)-(S18)), the pooling attention output is given by

$$\mathbb{p}_\mu = \sum_\beta s'^\beta \left(S_\mu + \sum_\nu V'_{\mu\nu} \mathbb{z}^\beta_\nu \right) = \sum_\beta s'^\beta \mathbb{z}'^\beta_\mu \quad \text{with} \;\; s'^\beta = \texttt{Softmax}\left[\sum_{\rho\lambda} \frac{1}{\sqrt{d_h}} \left(S_\rho K'_{\rho\lambda} \mathbb{z}^\beta_\lambda \right) \right], \tag{S24}$$

where s'^β is the pooling attention score calculated from the encoder output $\mathbb{z}^\beta$ with set index β.

First, we demonstrate the attention score's relation to Z-loop tension, which serves as the order parameter of the topological phase. To this end, we plot the histogram of pooling attention scores. Table S7 shows the detailed hyperparameters used to obtain Figure S23(a,c). After obtaining the pooling attention score distribution for each set, we sample the highest 10 ($\sim 15\%$) and lowest 10 attention scores s'^β and corresponding snapshots $\{B_\beta | s'^\beta \geq s_{\text{high}}\}$ and $\{B_\beta | s'^\beta \leq s_{\text{low}}\}$. For each snapshot B_β with 36 Z-plaquette values, we calculate the loop expectation value $\langle Z_{\text{closed}} \rangle$ at different loop perimeters $4L = 4, 8, 12, 16, 20, 24$ by multiplying Z-plaquettes inside the loop. Figure S23(b,d) shows the mean and the standard error of the mean of loop expectation value $\langle Z_{\text{closed}} \rangle$. The mean and standard error are over 10 high (low) attention score snapshots. For snapshots in the topological phase, PAB assigns a high attention score to the snapshots with vanishing loop tensions. The lower the loop tension, the higher the attention score assigned to the snapshot. Since the module conducts weighted sum over set index β where weight is the attention score, PAB acts as an automated importance sampler within a given set $\mathbb{X}_i$.

We then analyze how PAB makes a decision in testing. After Eq. (S24), the output is obtained through layer normalization, residual connection, and single-layer perception:

$$\mathbb{p}'_\mu = \texttt{LayerNorm}(\sum_\beta s'^\beta \mathbb{z}'^\beta_\mu) \tag{S25}$$

$$y(\mathbb{X}) = \texttt{Sigmoid}\left(\sum_\mu W_\mu \; \texttt{LayerNorm}\left[\mathbb{p}'_\mu + \texttt{rFF}_{\mu\nu}(\mathbb{p}'_\nu) \right] + b \right). \tag{S26}$$

where $\mathbb{z} = \texttt{Encoder}(\mathbb{x})$, and $\texttt{Encoder}$ is a MLP for PAB model. To simplify the process, we will compare between $\mathbb{z}'^\beta_\mu$-vector and W_μ-vector, ignoring latter $\texttt{LayerNorm}$ and residual layer which gives a minor shift in $\mathbb{z}'^\beta_\mu$. Figure S24 compares normalized encoder output from high (low) attention score snapshots (from Figure S23) with the final layer weight matrix.

We notice that $\mathbb{z}'^\beta_\mu$-vector for high and attention score snapshots are parallel to the W_μ-vector. Meanwhile, $\mathbb{z}'^\beta_\mu$-vector from low attention score in the trivial phase is anti-parallel to the W_μ-vector. This final $\texttt{Sigmoid}$ activation function then determines the output $y(\mathbb{X}_i)$: if the inner product between the importance-sampled encoder output $\mathbb{z}'^\beta_\mu$ and weight vector W_μ is positive (negative), it gives the final output of topological phase $y > 0.5$ (trivial phase $y < 0.5$). When the snapshots with high loop expectation values and high attention scores are no longer dominant, the weighted average of encoder output becomes anti-parallel with the final weight vector, and the machine prediction is no longer 'topological.'

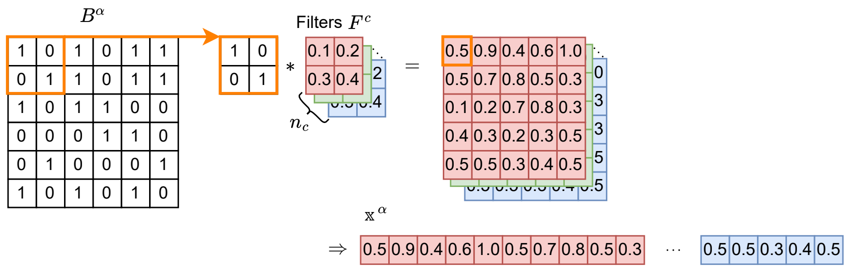

Figure S1. **Schematic example of convolution layer.** Here, we applied convolution layer to $N_q = 36$ bit-string with $N_r = 6$ rows and $N_c = 6$ columns. Convolution operation denoted as '$*$' involves summing over element-wise multiplication of part of a bit-string and a convolution filter. n_c represents the number of 2×2 convolution filter, and $\mathbf{x}^\alpha$ is the output after flattening convolution output into vector. We use a stride of 1 and no padding.

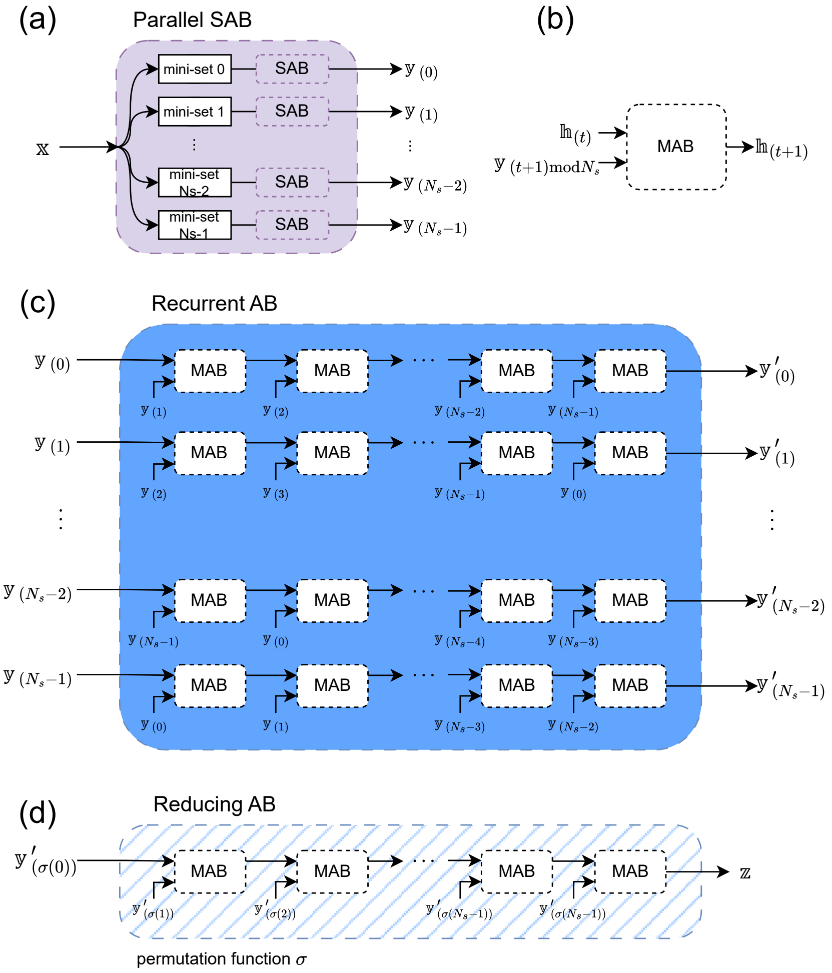

Figure S2. **Details of various blocks in the QuAN architecture.** (a) The inner structure of the parallel SAB. (b) Multihead-attention block (MAB) inside recurrent and reducing attention block (RecAB, RedAB). (c) The inner structure of the recurrent attention block (RecAB). (d) The inner structure of the reducing attention block (RedAB). $\sigma : S \to S$ is the random permutation function that permutes mini-set index $S = \{0, 1, \cdots, N_s - 1\}$.

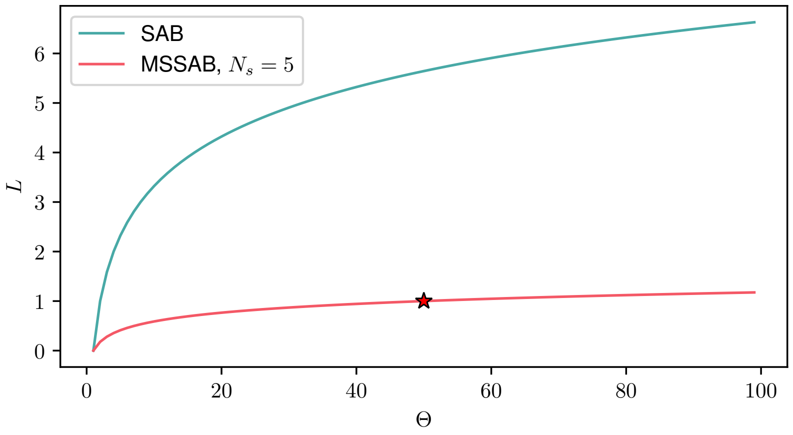

Figure S3. **Number of layers L required for SAB and MSSAB to reach moments of order Θ.** We omit the ceiling ($\lceil \cdot \rceil$) operation in the plot for simplicity. For SAB, the green curve plots $L = \log_2 \Theta$. For MSSAB, we plot $L = \log_{2N_s^2} \Theta$ for $N_s = 5$ (red curve). The red star marks 1 layer of MSSAB with N_s, the setup used in our RQC training in SM section D.

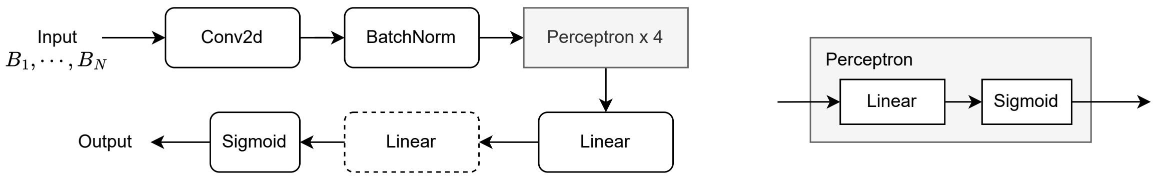

Figure S4. **Schematic MLP architecture.** The inner structure of the perceptron block is shown in the right panel. The dashed blocks indicate inter-set-element operations. Blocks with solid lines act on each set element independently.

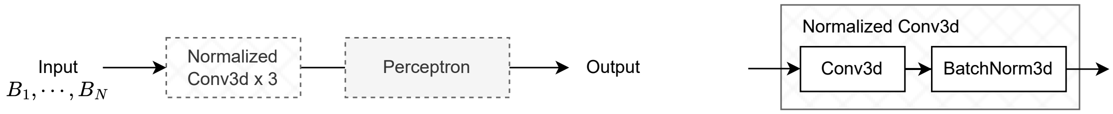

Figure S5. **Schematic CNN architecture.** The dashed blocks indicate inter-set-element operations. Blocks with solid lines act on each set element independently.

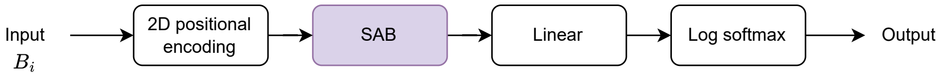

Figure S6. **Schematic transformer architecture.**

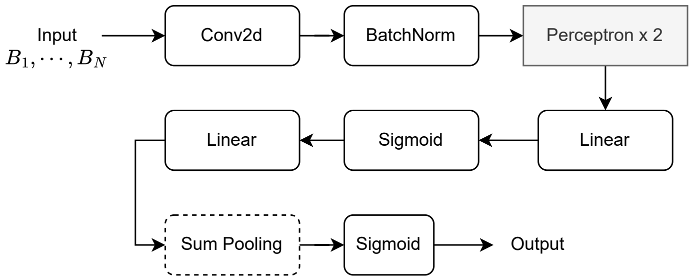

Figure S7. **Schematic SMLP architecture.** The dashed blocks indicate inter-set-element operations. Blocks with solid lines act on each set element independently.

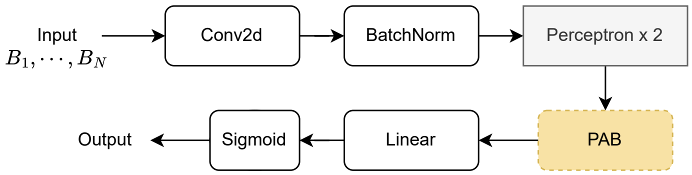

Figure S8. **Schematic PAB architecture.** The dashed blocks indicate inter-set-element operations. Blocks with solid lines act on each set element independently.

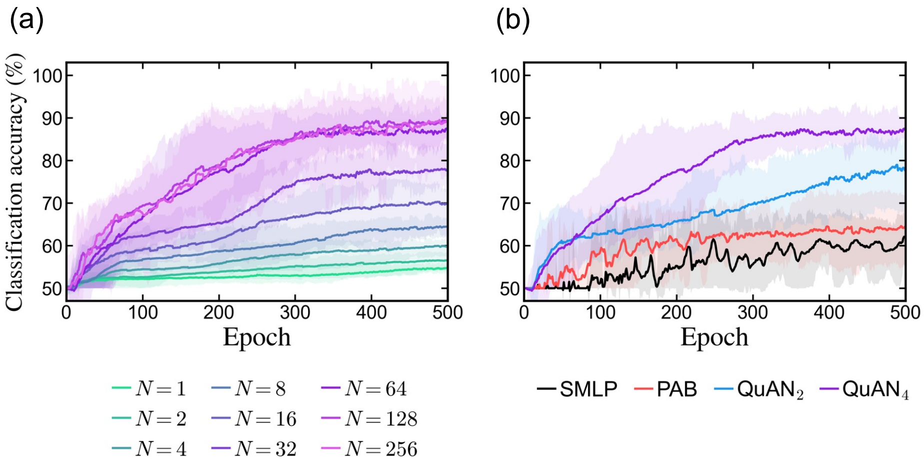

Figure S9. **Validation classification accuracy learning curves for driven hard-core Bose-Hubbard model. (a)** Validation classification accuracy curves at different set sizes N using QuAN$_4$. **(b)** Validation accuracy curves with different architectures (SMLP, PAB, QuAN$_2$ and QuAN$_4$) using $N = 64$. The solid line shows the mean of 10 independently trained models, and shaded regions show the minimum and maximum accuracy at each epoch.

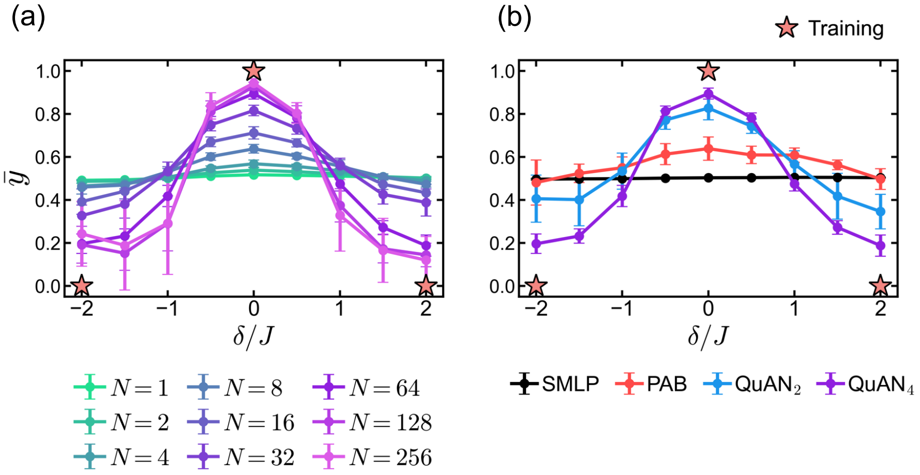

Figure S10. **Average confidence $\bar{y}$ as a function of detuning δ/J for driven hard-core Bose-Hubbard model.** The error bar represents the standard error of averaged confidence over 10 independent training. **(a)** Average confidence by QuAN$_4$ with varying set sizes N. **(b)** Average confidence with different architectures (SMLP, PAB, QuAN$_2$ and QuAN$_4$) using $N = 64$. The red stars indicate the training points $\delta/J = 0, \pm 2$.

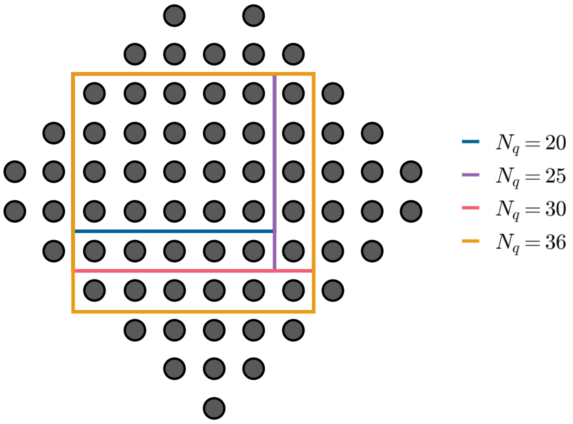

Figure S11. **Layouts of Google Sycamore processor**. Sycamore processor with 70 qubits (dark grey circles). The subarrays used for system sizes $N_q = 20, 25, 30$, and 36 are marked in colored boxes.

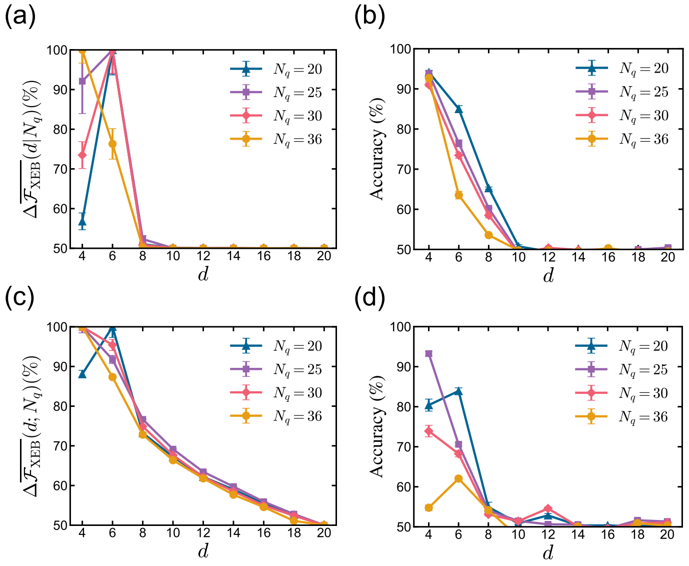

Figure S12. **Benchmarking QuAN's accuracy for RQC depth discrimination with relative XEB. (a)** Relative XEB (Eq. (S22)) for bit-strings from noiseless simulations as a function of circuit depth d with varying system sizes N_q. The markers show the relative averaged XEB over $N_c = 50$ different circuit instances, and the error bars show the standard errors. **(b)** Validation accuracy of QuAN_{50}, trained with noiseless simulation data, on noiseless simulation data. We train 8 independent models at each circuit depth d and show the averaged accuracy (marker) and the standard error (error bar). Same as main text Fig. 3D. **(c)** Relative XEB for experimentally collected bit-strings, as a function of circuit depth d with varying system sizes N_q. **(d)** Validation accuracy of QuAN_{50}, trained with noiseless simulation data, on noisy experimental data. Same as main text Fig. 3G.

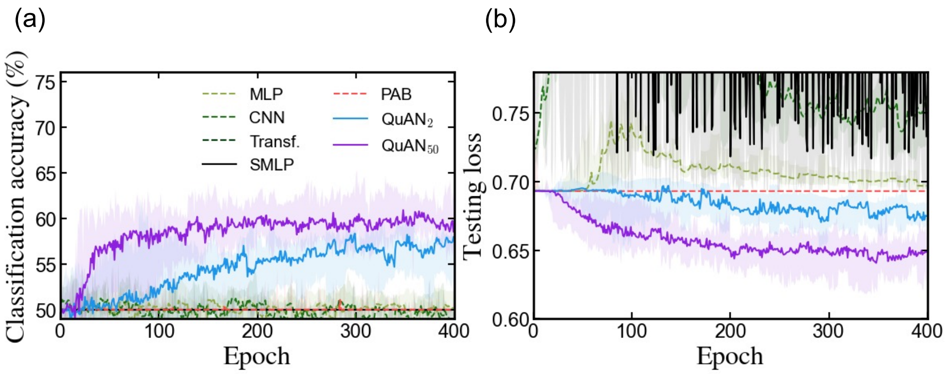

Figure S13. **Random quantum circuit learning curve of test classification accuracy.** **(a)** The learning curve of test classification accuracy compared at different architectures using $N = 10000$. **(b)** The learning curve of test loss compared at different architectures. We use testing data from the system size $N_q = 25$ and shallow depth $d = 8$. The solid line indicates the median of 8 independently trained models, and shaded regions show the minimum and maximum test accuracy among 8 models at each epoch.

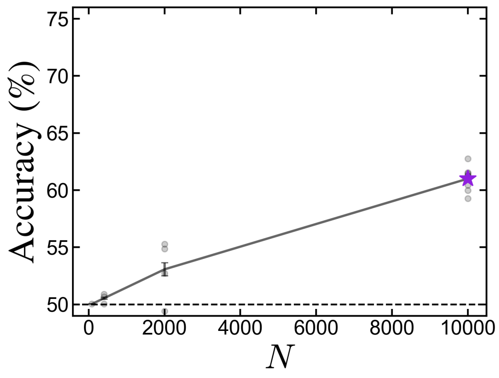

Figure S14. **Random quantum circuit test classification accuracy as a function of set size.** Test classification accuracy of QuAN$_{50}$ using $N_q = 25$ and $d = 8$ and 20, with varying set size $N = 80$, 400, 2000 and 10000. Each gray dot represents the 5 independently trained models, and the purple star represents the optimal accuracy we use in the main text. The black solid lines with error bars represent the averaged accuracy over 5 models.

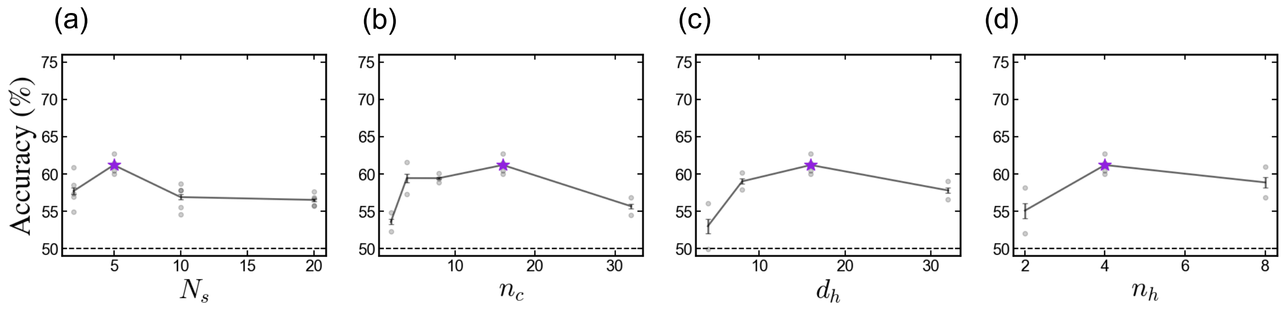

Figure S15. **Test classification accuracy of QuAN$_{50}$ with varying hyperparameters.** **(a)** number of mini-sets N_s, **(b)** number of convolutional filters n_c, **(c)** size of hidden dimension d_h, and **(d)** number of heads in multi-head attention blocks n_h. Each gray dot represents the 5 independently trained models, and the purple star represents the optimal accuracy we use in the main text. The black solid lines with error bars represent the average accuracy and standard error over 5 models.

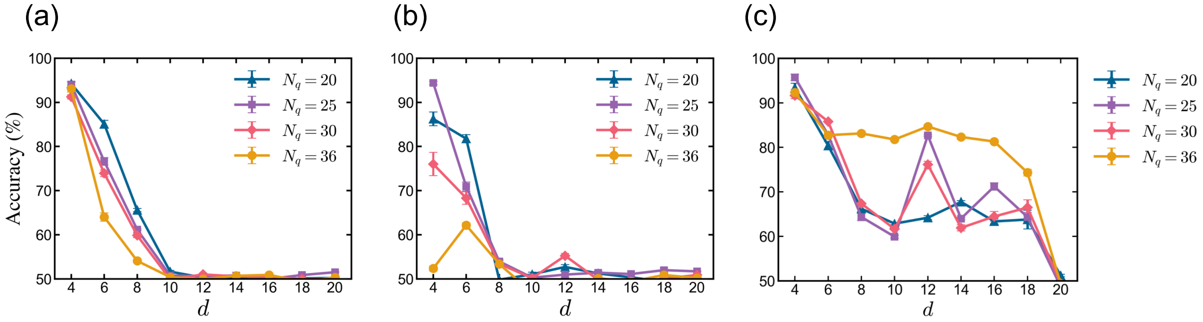

Figure S16. **Random quantum circuit test classification accuracy as a function of circuit depth d.** **(a)** Test classification accuracy (marker) and standard error (error bar) of QuAN$_{50}$ trained and tested with both simulated data of N_q qubits to distinguish depth d and 20, over 8 independently trained model. **(b)** The accuracy and standard error of QuAN$_{50}$ trained on simulated data and tested with experimental data of depth d and 20, over 8 independently trained models. **(c)** The accuracy of QuAN$_{50}$ trained and tested with both experimental data averaged over 5 independently trained models.

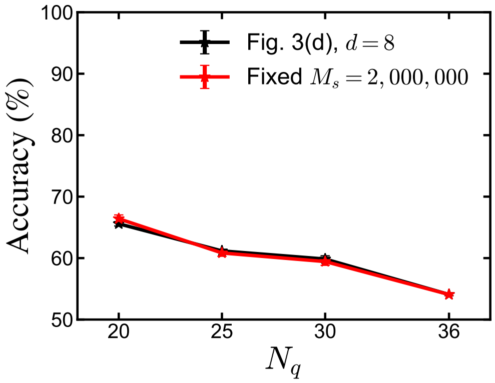

Figure S17. **Random quantum circuit test classification accuracy as a function of system size N_q.** Testing classification accuracy of QuAN$_{50}$ trained with simulated data to distinguish simulated data of depth $d = 8$ and 20, as a function of system size N_q. (Black) We use different sample sizes for different system sizes ($M_s = 5 \times 10^5$ for $N_q = 20, 25, 30$; $M_s = 2 \times 10^6$ for $N_q = 36$) to be consistent with the experimental data volumes. Note that these accuracies are the same as taking points with different N_q but with fixed $d = 8$ in the main text Fig. 3D. (Red) Testing accuracy of QuAN$_{50}$ trained with 70% of $M_s = 2 \times 10^6$ bit-strings for all system sizes N_q. We train 5 independent models and show the average (markers) and standard error (error bar) of the testing accuracy.

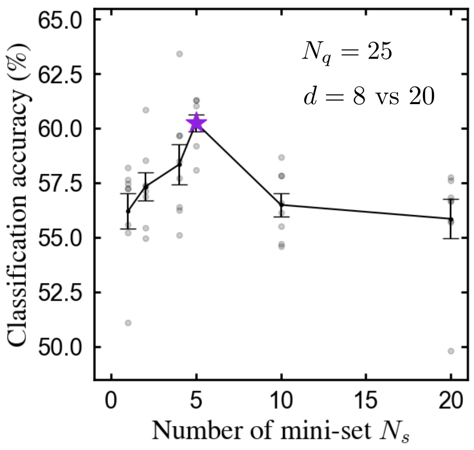

Figure S18. **Model ablation study for accessible effective moment.** Classification accuracy in learning the relative complexity of depth $d = 8$ and $d = 20$ on a $N_q = 25$ qubit system, as a function of the number of mini-sets N_s ($\text{QuAN}_{2N_s^2}$), where the maximum accessible moment order is $2N_s^2$. We report the averaged classification accuracy (black dot) over 8 independently trained models along with the corresponding standard error (error bar). The purple star denotes the optimal accuracy we use in the main text, and each gray dots denote the accuracy for 8 independently trained models.

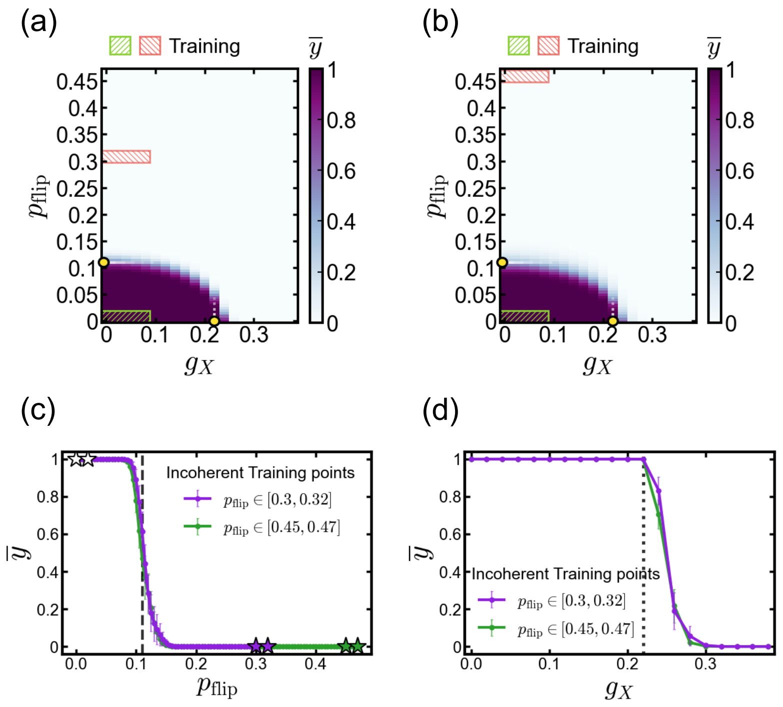

Figure S19. **Robustness of the phase boundary against shifting a training point.** **(a,b)** The decodability phase diagram of the toric code state by QuAN_2 under coherent ($p_{\text{flip}} \in \{0.0, 0.005, 0.01, 0.015, 0.02\}$, over range of $g_X \in \{0, 0.02, 0.04, 0.06, 0.08\}$) and incoherent noise for two different incoherent training points; **(a)** $p_{\text{flip}} \in \{0.3, 0.305, 0.31, 0.315, 0.32\}$ and **(b)** $p_{\text{flip}} \in \{0.45, 0.455, 0.46, 0.465, 0.47\}$, over the same range of g_X. The regions in the phase space that support the training data are marked with hatch marks. The known thresholds are marked along the $g_X = 0$ axis at $p_c = 0.11$ and along the $p_{\text{flip}} = 0$ at $g_c \approx 0.22$. Note that we train QuAN 10 independent times and report the averaged confidence. **(c,d)** Average confidence $\bar{y}$ by QuAN_2 for different incoherent training points, along the axis **(c)** $g_X = 0$ and **(d)** $p_{\text{flip}} = 0$. The error bar shows the standard error for $\bar{y}$ over 10 independent model training.

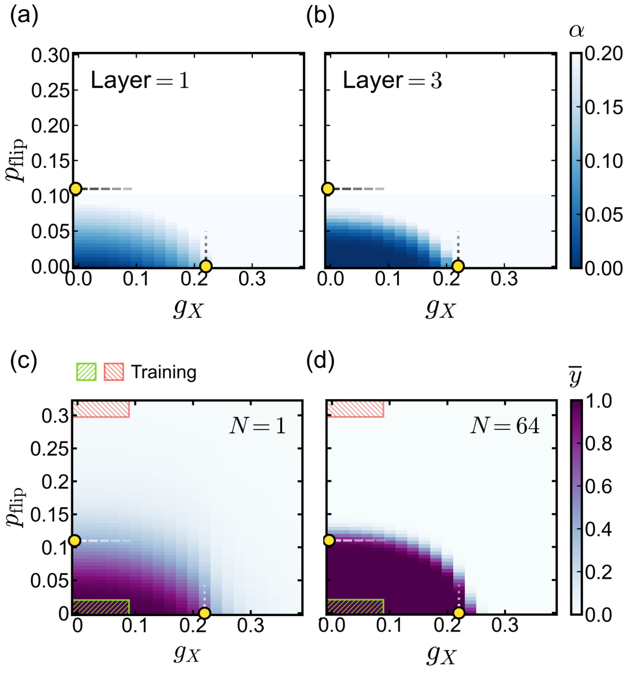

Figure S20. **Toric code phase diagram using locally error-corrected decoration (LED) and QuAN. (a,b)** Phase diagrams of the toric code state with both coherent noise (transverse field strength g_X with fixed $g_Z = 0.14$) and incoherent noise $p_{\rm flip}$ as a function of Z-loop tension α using **(a)** 1 layer and **(b)** 3 layers of locally error-corrected decoration (LED) [61]. Yellow circles with vanishing dashed lines represent the known thresholds at $p_c \approx 0.11$ and $g_c \approx 0.22$. **(c,d)** Phase diagram constructed using averaged confidence $\bar{y}$ by QuAN_2, presented in the main text Fig. 4C,D. The hatched regions mark the training data ranges.

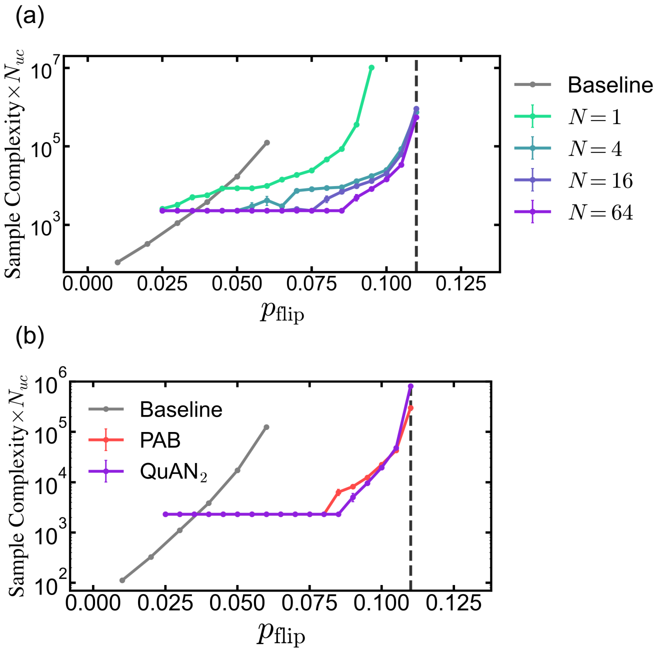

Figure S21. **Benchmarking to locally error-corrected decoration (LED) using sample complexity.** Sample complexity of **(a)** QuAN_2 with varying set size N and **(b)** PAB and QuAN_2 with set size $N = 64$, using data from different $p_{\rm flip}$ along the $g_X = 0$ axis. $N_{uc} = 36$ is the number of unit cells in snapshots. We use sample complexity of $\langle Z_{\rm closed} \rangle$ as the *baseline* (marked in gray) [61]. Sample complexity beyond the threshold (dashed line) is not defined.

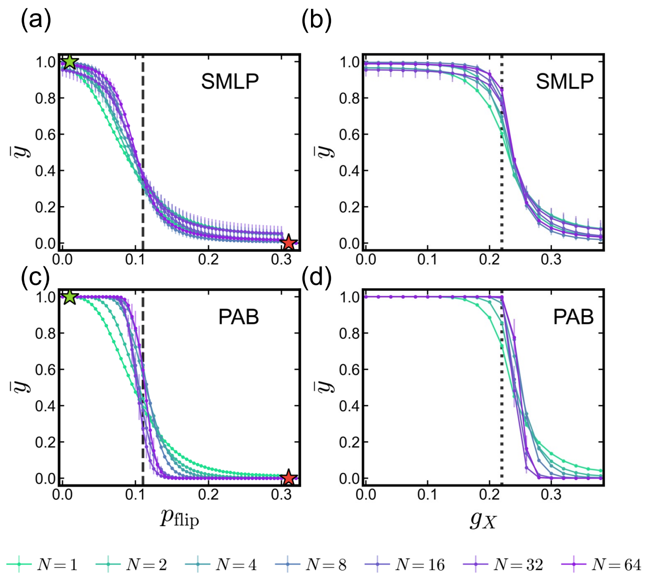

Figure S22. **Benchmarking PAB results to SMLP using average confidence.** **(a)** Average confidence $\bar{y}$ (marker) and its standard error over 10 independent training (error bar) by SMLP with varying set sizes N, along the axis $g_X = 0$ with different incoherent noise rates $p_{\rm flip}$. Green and pink stars represent the training points. **(b)** Average confidence $\bar{y}$ by SMLP with varying set sizes N, along the axis $p_{\rm flip} = 0$ with different coherent noise strength g_X. **(c)** Average confidence $\bar{y}$ by PAB with varying set sizes N, along the axis $g_X = 0$ with different incoherent noise rates $p_{\rm flip}$. **(d)** Average confidence $\bar{y}$ by PAB with varying set sizes N, along the axis $p_{\rm flip} = 0$ with different coherent noise strength g_X.

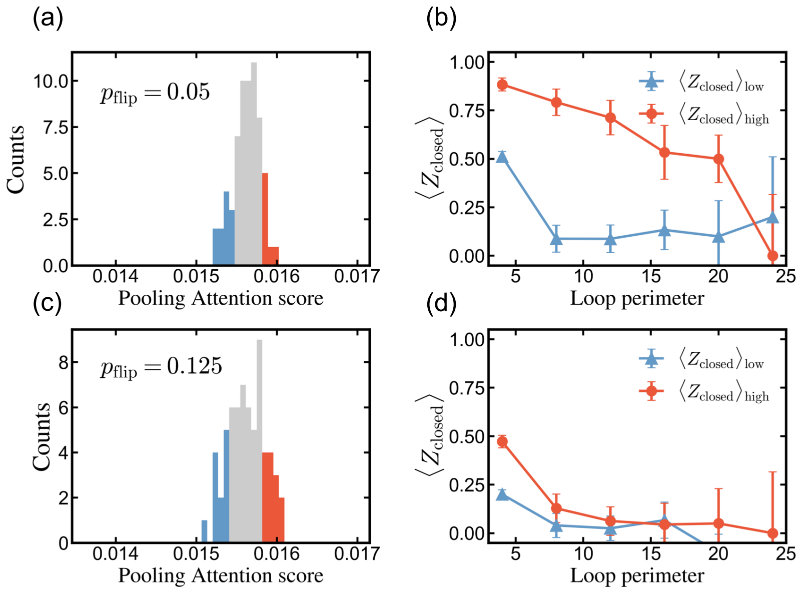

Figure S23. **Pooling attention score analysis for different phases in toric code.** **(a,c)** Pooling attention score histogram from **(a)** topological state with $(g_X, p_{\rm flip}) = (0, 0.05)$ (see main text Fig. 4I) and **(c)** trivial state with $(g_X, p_{\rm flip}) = (0, 0.125)$. **(b,d)** Loop expectation value $\langle Z_{\rm closed}\rangle$ (marker) and its standard error (error bar) as a function of the loop perimeter with high and low attention score in **(b)** topological $(g_X, p_{\rm flip}) = (0, 0.05)$ (see main text Fig. 4J) and **(d)** trivial state $(g_X, p_{\rm flip}) = (0, 0.125)$.

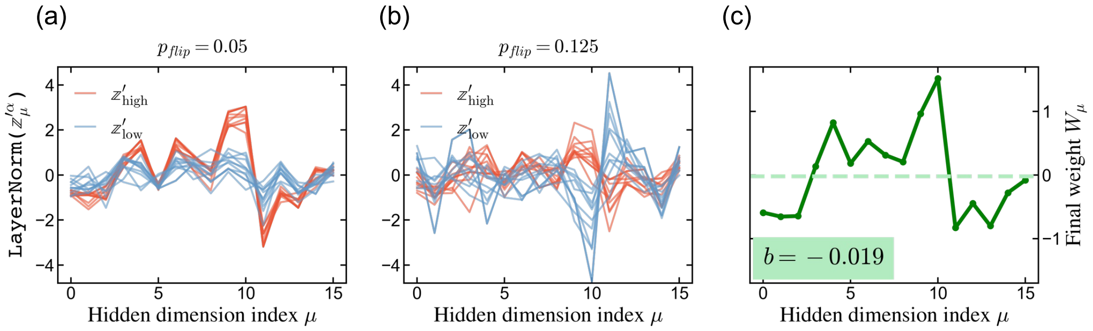

Figure S24. **Encoder output analysis for different phases in toric code (a,b)** Encoder output vector $\mathbb{z}$ for snapshots from high (red) and low (blue) attention score in **(a)** topological $(g_X, p_{\text{flip}}) = (0, 0.05)$ and **(b)** trivial state $(g_X, p_{\text{flip}}) = (0, 0.125)$. **(c)** The final weight W_μ in final perception before machine output (see Eq. (S26)) shows a similar shape compared to the encoder output vector with a high attention score. The dashed line represents the value of bias b.

Model hyperparameter	
Model	SMLP, PAB, QuAN_2, QuAN_4
Number of mini-sets (N_s)	1
Number of MSSAB layer (L)	0∼2
Number of 2×2 Conv. channel (n_c)	7∼8
Attention block: Hidden spatial dimension (d_h)	16
Attention block: Number of heads (n_h)	4
Attention block: activation function for residual connection	`Sigmoid`

Training hyperparameter	
Optimizer	`Adam`($\beta_1 = 0.9,\ \beta_2 = 0.999,\ \epsilon = 1 \times 10^{-8}$)
L2 coefficient	5×10^{-5}
Learning rate	1×10^{-4}
LR schedule	`StepLR`($\text{stepsize} = 200, \gamma = 0.65$)
Epoch	500
Dataset shuffling period	10
Batchsize	$80000/N$
Initialization	Default
GPU	A100 (80GB)

Table S1. Model setting and training hyperparameters for the driven hard-core Bose-Hubbard model. We use set size of range $N = 1 \sim 256$.

	SMLP	PAB	QuAN_2	QuAN_4
Encoder	Conv(1, 8, 2, 1, `BatchNorm`)<br>SLP(72, 48, `Sigmoid`)<br>SLP(48, 16, `Sigmoid`)	Conv(1, 8, 2, 1, `BatchNorm`)<br>SLP(72, 48, `Sigmoid`)<br>SLP(48, 16, `Sigmoid`)	Conv(1, 8, 2, 1, `BatchNorm`)<br>MSSAB*(72, 16, 4, 1)	Conv(1, 7, 2, 1, `BatchNorm`)<br>MSSAB*(63, 16, 4, 1)<br>MSSAB*(16, 16, 4, 1)
Decoder	SLP(16, 48, `Sigmoid`)<br>SLP(48, 1)<br>Sum*, `Sigmoid`	PAB*(16, 16, 4)<br>SLP(16, 1, `Sigmoid`)	PAB*(16, 16, 4)<br>SLP(16, 1, `Sigmoid`)	PAB*(16, 16, 4)<br>SLP(16, 1, `Sigmoid`)

Table S2. Architecture parameters used in models sketched in main text Fig. 2E-G. We use data from $N_q = 16$ with 4×4 geometry for training and testing. Each layer's arguments are as follows: Convolutional layer as Conv($n_{c,\text{in}}$, $n_{c,\text{out}}$, kernel, stride, normalization). Single-layer perception as SLP(d_{in}, d_{out}, activation). MSSAB($d_{h,\text{in}}$, $d_{h,\text{out}}$, n_h, N_s). PAB($d_{h,\text{in}}$, $d_{h,\text{out}}$, n_h). The asterisk (*) denotes the module operates on a set dimension.

Model hyperparameter	
Model	QuAN_{50}
Number of mini-sets (N_s)	5
Number of MSSAB layer (L)	1
Number of 1×1 Conv. channel (n_c)	16
Attention block: Hidden spatial dimension (d_h)	16
Attention block: Number of heads (n_h)	4
Attention block: activation function for residual connection	`ReLU`

Training hyperparameter	
Optimizer	`Adam`($\beta_1 = 0.9,\ \beta_2 = 0.999,\ \epsilon = 1 \times 10^{-8}$)
L2 coefficient	5×10^{-5}
Learning rate	3.5×10^{-5}
LR schedule	`StepLR`(stepsize $= 100 \sim 200, \gamma = 0.65$)
Epoch	400
Dataset shuffle period	10
Batchsize	20
Initialization	`xavier_normal`
GPU	A100 (80GB)

Table S3. Model setting and hyperparameters used to train the model with random quantum circuit data. We use set size of $N = 10000$.

	MLP	CNN	Transf.
Encoder	Conv(1, 16, 2, 1, `BatchNorm`)<br>SLP(256, 16, `Sigmoid`)<br>SLP(16, 16, `Sigmoid`)	Conv3d(1, 4, (500,2,2), (50,1,1), `BatchNorm`)<br>Conv3d(4, 4, (50,2,2), (5,1,1), `BatchNorm`)<br>Conv3d(4, 16, (5,2,2), (1,1,1), `BatchNorm`)	PE2d($d = 16$)<br>SAB(32, 16, 4)
Decoder	SLP(16, 16, `Sigmoid`)<br>SLP(16, 1, `Sigmoid`)<br>SLP*(N, 1, `Sigmoid`)	SLP(1600, 1, `Sigmoid`)	SLP(400, 1, `log softmax`)

	SMLP	PAB	QuAN
Encoder	Conv(1, 16, 2, 1, `BatchNorm`)<br>SLP(256, 48, `Sigmoid`)<br>SLP(48, 16, `Sigmoid`)	Conv(1, 16, 2, 1, `BatchNorm`)<br>SLP(256, 48, `Sigmoid`)<br>SLP(48, 16, `Sigmoid`)	Conv(1, 16, 2, 1, `BatchNorm`)<br>MSSAB*(256, 16, 4, N_s)
Decoder	SLP(16, 48, `Sigmoid`)<br>SLP(48, 1)<br>Sum*, `Sigmoid`	PAB*(16, 16, 4)<br>SLP(16, 1, `Sigmoid`)	PAB*(16, 16, 4)<br>SLP(16, 1, `Sigmoid`)

Table S4. Detailed model architectures used in main text Fig. 3E. We train using data from $N_q = 25$ with 5×5 geometry. Each layer's arguments as followed: Convolutional layer as Conv($n_{c,\text{in}}$, $n_{c,\text{out}}$, kernel, stride, normalization). Single perception as SLP(d_in, d_out, activation). 2D Positional encoding as PE2d(d_h). SAB($d_{h,\text{in}}$, $d_{h,\text{out}}$, n_h) and MSSAB($d_{h,\text{in}}$, $d_{h,\text{out}}$, n_h, N_s). PAB($d_{h,\text{in}}$, $d_{h,\text{out}}$, n_h). * denotes the module operates over a set dimension.

Model hyperparameter	
Model	SMLP, PAB, QuAN_2
Number of mini-sets (N_s)	1
Number of MSSAB layer (L)	0,1
Conv. channel (n_c)	No convolution
Attention block: Hidden spatial dimension (d_h)	16
Attention block: Number of heads (n_h)	4
Attention block: activation function for residual connection	`ReLU`

Training hyperparameter	
Optimizer	`Adam`($\beta_1 = 0.9,\ \beta_2 = 0.999,\ \epsilon = 1 \times 10^{-8}$)
L2 coefficient	5×10^{-5}
Learning rate	1×10^{-4}
LR schedule	`StepLR`(stepsize $= 100, \gamma = 0.65$)
Epoch	200
Dataset shuffle period	10
Batchsize	$32768/N$
Initialization	`xavier_normal`
GPU	a100, 80GB

Table S5. Model setting and hyperparameters used to train the model for toric code problem. We use set size of range $N = 1 \sim 64$.

	SMLP	PAB	$QuAN_2$
Encoder	SLP(36, 48, `Sigmoid`)	SLP(36, 48, `Sigmoid`)	SLP(36, 48, `Sigmoid`)
	SLP(48, 16, `Sigmoid`)	SLP(48, 16, `Sigmoid`)	SLP(48, 16, `Sigmoid`)
			MSSAB*(36, 16, 4, 1)
Decoder	SLP(16, 48, `Sigmoid`)	PAB*(16, 16, 4)	PAB*(16, 16, 4)
	SLP(48, 1)	SLP(16, 1, `Sigmoid`)	SLP(16, 1, `Sigmoid`)
	Sum*, `Sigmoid`		

Table S6. Detailed model architectures used in main text Fig. 2E-G. We use data from $N_q = 16$ with 4×4 geometry. (Each layer's arguments are as follows: Convolution layer as Conv($n_{c,\text{in}}$, $n_{c,\text{out}}$, kernel, stride, normalization). Single perception as SLP(d_{in}, d_{out}, activation). MSSAB($d_{h,\text{in}}$, $d_{h,\text{out}}$, n_h, N_s). PAB($d_{h,\text{in}}$, $d_{h,\text{out}}$, n_h)). * denotes the module operates on a set dimension.

Model training/testing hyperparameter		
Model	PAB	
Training set size (N)	32	
Model index out of 10	3	
Attention block: Head index	3	
**Testing data details**		
Testing set size (N)	64	
Data points (g_X, p_{flip})	$(0, 0.05)$	$(0, 0.125)$
Set number index (i)	116	43

Table S7. Model hyperparameters and testing data details used in Figure S23.

Model	δ/J	Mean of $\bar{y}$	Standard error of $\bar{y}$	$\bar{y}$ for independent training									
SMLP	-2	0.496	0.001	0.498	0.488	0.502	0.497	0.498	0.494	0.498	0.495	0.497	0.491
	-1.5	0.498	0.001	0.498	0.494	0.503	0.497	0.497	0.497	0.499	0.496	0.499	0.495
	-1	0.498	0.001	0.499	0.495	0.504	0.498	0.499	0.498	0.499	0.497	0.499	0.496
	-0.5	0.501	0.001	0.502	0.499	0.506	0.5	0.503	0.502	0.5	0.498	0.501	0.5
	0	0.503	0.001	0.503	0.5	0.507	0.502	0.505	0.504	0.501	0.499	0.502	0.503
	0.5	0.503	0.001	0.504	0.502	0.508	0.502	0.507	0.506	0.501	0.499	0.501	0.503
	1	0.505	0.001	0.506	0.502	0.509	0.504	0.51	0.508	0.501	0.5	0.502	0.504
	1.5	0.505	0.001	0.506	0.503	0.509	0.503	0.509	0.508	0.501	0.5	0.502	0.504
	2	0.503	0.001	0.505	0.501	0.506	0.501	0.506	0.506	0.501	0.499	0.501	0.503
$QuAN_2$	-2	0.405	0.038	0.333	0.626	0.481	0.476	0.39	0.367	0.243	0.26	0.478	0.399
	-1.5	0.401	0.043	0.273	0.582	0.527	0.505	0.442	0.309	0.241	0.351	0.518	0.263
	-1	0.533	0.029	0.388	0.677	0.587	0.554	0.593	0.498	0.422	0.53	0.603	0.482
	-0.5	0.771	0.015	0.738	0.785	0.724	0.7	0.769	0.807	0.838	0.809	0.732	0.806
	0	0.827	0.019	0.801	0.832	0.74	0.742	0.842	0.875	0.909	0.875	0.793	0.858
	0.5	0.743	0.012	0.693	0.751	0.707	0.697	0.746	0.772	0.796	0.78	0.737	0.748
	1	0.567	0.022	0.517	0.607	0.577	0.667	0.611	0.522	0.512	0.531	0.65	0.472
	1.5	0.417	0.037	0.38	0.467	0.47	0.602	0.455	0.336	0.287	0.333	0.562	0.278
	2	0.346	0.028	0.367	0.421	0.337	0.513	0.351	0.3	0.248	0.324	0.381	0.214
$QuAN_4$	-2	0.196	0.016	0.243	0.2	0.225	0.284	0.129	0.189	0.145	0.147	0.186	0.208
	-1.5	0.232	0.012	0.225	0.232	0.244	0.3	0.182	0.254	0.182	0.242	0.243	0.214
	-1	0.418	0.017	0.385	0.43	0.418	0.528	0.366	0.428	0.347	0.424	0.463	0.39
	-0.5	0.812	0.009	0.794	0.826	0.838	0.853	0.794	0.814	0.772	0.827	0.82	0.781
	0	0.894	0.009	0.899	0.882	0.933	0.921	0.927	0.887	0.901	0.863	0.872	0.855
	0.5	0.782	0.008	0.777	0.796	0.793	0.82	0.794	0.764	0.801	0.747	0.755	0.775
	1	0.474	0.012	0.474	0.507	0.492	0.5	0.499	0.427	0.519	0.442	0.423	0.457
	1.5	0.272	0.011	0.261	0.292	0.283	0.306	0.277	0.215	0.316	0.223	0.253	0.29
	2	0.187	0.018	0.232	0.239	0.246	0.177	0.208	0.1	0.239	0.132	0.143	0.158

Table S8. Data values in Fig. 2C-E for set size $N = 64$, 10 independent training.

N_r	N_c	d	Average classification accuracy	Standard error	Classification accuracy for independent training							
4	5	4	94.130	0.815	91.187	94.227	96.84	96.347	93.373	96.267	94	90.8
4	5	6	84.998	1.651	84.987	81.747	87.173	77.587	86.253	92.107	81.173	88.96
4	5	8	65.273	0.563	64.507	67.947	66.32	64.68	66	63.067	63.653	66.013
4	5	10	50.753	0.929	51.84	48.8	51.48	53.907	45.92	53.653	50.693	49.733
4	5	12	49.480	0.434	51.12	48	48.093	50.333	49.8	50.893	48.76	48.84
4	5	14	49.812	0.265	49.12	48.88	50.547	50.987	49.84	49.947	50.107	49.067
4	5	16	48.533	0.426	50	47.32	48.293	47.347	49.16	47.813	50.467	47.867
4	5	18	49.087	0.566	48.147	49.693	51.133	45.787	48.907	50.24	49.587	49.2
4	5	20	48.021	0.616	50.083	48.361	45.222	49.306	49.556	48.639	46.444	46.556
5	5	4	93.907	1.326	98.213	97.067	94.427	91.573	88.573	98.48	92.653	90.267
5	5	6	76.430	1.720	87.08	77.813	78.267	74.747	74.24	71.68	72.587	75.027
5	5	8	60.247	0.389	61.307	60.44	61.28	60.253	60.347	58.107	59.213	61.027
5	5	10	49.105	0.415	50.347	49.027	50.627	49.547	46.893	48.76	49.267	48.373
5	5	12	50.108	0.494	52.56	49.587	49.587	50.533	51.04	49.733	50.147	47.68
5	5	14	49.265	0.613	48.92	46.667	50.88	47.507	48.933	50.28	48.973	51.96
5	5	16	49.348	0.226	48.12	49.187	50.133	49.787	49.573	48.827	49.373	49.787
5	5	18	49.998	0.572	49.453	48.773	50.04	50.973	48.373	53.187	48.493	50.693
5	5	20	50.444	0.619	47.361	50.194	50.75	51.889	51.556	52.139	48.278	51.389
5	6	4	91.010	0.974	87.32	93.813	90.293	90.133	96.027	88.787	90.547	91.16
5	6	6	73.448	1.498	77.933	73.28	81.32	68.333	70.307	70.907	72.533	72.973
5	6	8	58.487	1.203	57.453	57.947	60.56	56.707	64.947	59.493	53.12	57.667
5	6	10	48.967	0.465	49.333	50.707	51.053	48.04	47.693	49.013	47.84	48.053
5	6	12	50.398	0.414	49.92	50.587	49.92	49.88	49.347	49.56	50.987	52.987
5	6	14	49.883	0.345	49.693	49.373	48.453	49.973	50.587	49.48	51.787	49.72
5	6	16	50.013	0.414	50.253	51.413	51.067	49.92	49.507	47.573	49.867	50.507
5	6	18	49.542	0.456	49.293	48.88	50.56	48.267	49.2	49.387	52.24	48.507
5	6	20	47.896	0.871	46.806	45.972	44.333	51.972	49.25	49.389	49.167	46.278
6	6	4	92.700	1.737	85.633	90.867	95.133	98.733	95.267	96.5	93.9	85.567
6	6	6	63.525	1.915	73.033	66.467	63.667	68.233	60.7	60.133	56.733	59.233
6	6	8	53.567	0.238	53	53.5	54	53.367	52.9	52.9	54.767	54.1
6	6	10	49.750	0.292	49.6	49.633	50.4	50.267	48.367	49.967	50.9	48.867
6	6	12	49.708	0.256	50	48.933	50.3	48.5	49.833	49.3	50.267	50.533
6	6	14	49.487	0.324	48.633	50.367	50.2	50.133	48.567	50.567	48.367	49.067
6	6	16	50.300	0.170	49.567	49.933	50.9	50.767	50.333	50.033	50.8	50.067
6	6	18	49.308	0.328	48.8	49.267	47.333	49.8	49.667	49.433	49.7	50.467
6	6	20	49.507	0.638	49.605	52.368	49.211	47.895	48.816	51.579	49.737	46.842

Table S9. Data values in Fig. 3D, 8 independent training.

Model	Average classification accuracy (%)	Standard error	Classification accuracy for independent training							
MLP	49.425	0.387	49.000	50.693	50.360	49.600	47.840	50.413	49.547	47.947
CNN	49.887	0.546	49.707	46.920	50.920	49.760	51.973	50.711	50.421	48.684
Transf.	50.000	0.000	50.000	50.000	50.000	50.000	50.000	50.000	50.000	50.000
SMLP	50.368	0.213	50.253	50.000	50.000	50.000	51.253	50.026	51.413	50.000
PAB	50.592	0.170	51.080	50.507	50.973	50.360	51.000	49.868	50.000	50.947
QuAN2	56.217	0.816	51.093	57.240	55.227	57.240	55.600	57.467	57.653	58.213
QuAN50	60.247	0.389	61.307	60.440	61.280	60.253	60.347	58.107	59.213	61.027

Table S10. Data values in Fig. 3E, 8 independent training.

N_r	N_c	d	Average classification accuracy	Standard error	Classification accuracy for independent training							
4	5	4	80.400	3.437	89.867	78.533	94	83.333	73.133	82.6	76	65.733
4	5	6	83.917	1.814	84.4	76.067	85.467	79.533	83	90.667	82.8	89.4
4	5	8	54.950	2.732	50.867	50.2	52.4	48.267	49.933	66.8	55.733	65.4
4	5	10	50.858	0.612	54.067	50.267	49.933	52.667	49.8	50.333	50.333	49.467
4	5	12	52.859	1.089	50.267	51.933	56.067	50.933	58.467	51.867	50.867	52.467
4	5	14	50.350	0.195	50.8	50.467	50.133	50.733	50.667	49.333	49.933	50.733
4	5	16	50.342	0.493	50	50.8	50.467	49.333	51.867	48.133	50	52.133
4	5	18	50.142	0.672	47.8	49.6	48.267	50	52.467	50.067	52.867	50.067
4	5	20	50.573	0.298	50	50.556	50	50	52.222	50.278	50.278	51.25
5	5	4	93.258	1.416	95.867	95.933	93	94.6	87.8	95	96.733	87.133
5	5	6	70.608	1.006	74.733	68.867	73.667	66.733	69.933	69.733	72	69.2
5	5	8	54.050	0.770	53	58.667	53	53.2	52.6	52.667	54.867	54.4
5	5	10	51.475	0.836	51.533	50.2	50	49.6	51.733	56.6	51.4	50.733
5	5	12	50.617	0.667	48.467	51.067	50.267	53.2	47.8	51.6	51.733	50.8
5	5	14	50.559	0.894	48.267	53.467	53.067	49.267	47.467	51.667	52.467	48.8
5	5	16	49.542	0.386	50.2	49.8	50.733	49.667	50.533	47.867	49.2	48.333
5	5	18	51.633	0.851	51.133	55.133	54.067	49.933	50.2	48.333	51.533	52.733
5	6	4	73.917	3.312	68.333	89.2	81.667	60.4	76.4	71.2	74.267	69.867
5	6	6	68.250	1.808	69.4	60.067	75.867	68.733	69	68.2	71.333	63.4
5	6	8	53.100	0.894	52.133	55.933	56.4	49.333	52.867	53	51.067	54.067
5	6	10	51.408	0.541	51.133	50.467	54.067	50.133	51.533	49.667	51.6	52.667
5	6	12	54.575	0.810	57.667	52.733	55.333	53.733	56.133	51.467	53.067	56.467
5	6	14	50.117	0.656	49.6	51.467	51.4	50.133	52.267	49.533	49.933	46.6
5	6	16	49.642	0.687	51.8	50.4	49.4	50.6	46.933	51.8	48.4	47.8
5	6	18	50.867	0.457	50.8	48.933	50.4	50.067	51.067	51	53.133	51.533
5	6	20	51.042	0.633	49.167	51.111	54.167	51.528	48.75	51.806	50.556	51.25
6	6	4	54.763	1.628	55.367	49.567	55.467	53.433	53.6	51.367	64.1	55.2
6	6	6	62.042	1.219	62.6	62.967	63.867	58.3	63	56.067	63.567	65.967
6	6	8	54.154	1.720	57.5	52.367	53.833	52.9	58.033	46.533	51.1	60.967
6	6	10	47.462	0.572	44.467	46.233	48.833	48.8	48.567	47.233	48.233	47.333
6	6	12	45.446	0.786	46.133	44.3	46.667	47.767	48.1	42.6	42.933	45.067
6	6	14	50.033	0.995	51.8	47.333	51.167	51.133	44.7	51.267	50.5	52.367
6	6	16	47.450	1.148	49.633	49.733	48.2	48.267	45.267	50.1	41	47.4
6	6	18	50.904	0.515	50.067	50.233	52.467	50.733	50.3	51.9	48.767	52.767

Table S11. Data values in Fig. 3G, 8 independent training.

	p_{flip}	Mean of $\bar{y}$	Standard error	$\bar{y}$ for independent training									
$N=1$	0.050	0.892	0.010	0.914	0.909	0.844	0.829	0.901	0.901	0.906	0.897	0.906	0.911
	0.055	0.856	0.012	0.882	0.877	0.799	0.781	0.868	0.868	0.873	0.863	0.873	0.879
	0.060	0.817	0.014	0.847	0.839	0.749	0.729	0.831	0.83	0.837	0.824	0.836	0.843
	0.065	0.769	0.016	0.802	0.793	0.693	0.671	0.785	0.784	0.792	0.778	0.79	0.799
	0.070	0.723	0.017	0.759	0.749	0.64	0.617	0.742	0.741	0.749	0.733	0.747	0.756
	0.075	0.676	0.018	0.713	0.703	0.587	0.564	0.697	0.695	0.705	0.688	0.701	0.711
	0.080	0.625	0.019	0.664	0.652	0.532	0.508	0.648	0.645	0.655	0.638	0.651	0.661
	0.085	0.578	0.019	0.616	0.605	0.483	0.459	0.602	0.599	0.609	0.591	0.604	0.614
	0.090	0.531	0.019	0.568	0.556	0.435	0.412	0.555	0.552	0.563	0.544	0.557	0.567
	0.095	0.483	0.019	0.519	0.507	0.387	0.365	0.508	0.505	0.516	0.497	0.509	0.519
	0.100	0.440	0.019	0.474	0.462	0.345	0.323	0.465	0.462	0.473	0.454	0.465	0.475
	0.105	0.399	0.018	0.431	0.42	0.306	0.286	0.424	0.42	0.431	0.412	0.423	0.433
	0.110	0.358	0.017	0.389	0.378	0.269	0.25	0.382	0.379	0.389	0.371	0.382	0.391
	0.115	0.322	0.017	0.351	0.34	0.237	0.219	0.346	0.342	0.353	0.335	0.345	0.353
	0.120	0.288	0.016	0.314	0.303	0.207	0.19	0.311	0.307	0.317	0.301	0.309	0.316
	0.125	0.258	0.015	0.283	0.273	0.183	0.167	0.28	0.277	0.286	0.271	0.278	0.285
	0.130	0.228	0.014	0.249	0.24	0.157	0.143	0.249	0.245	0.254	0.239	0.246	0.253
$N=4$	0.05	0.999	0.000	1	0.999	1	0.999	1	1	0.999	0.999	0.998	0.999
	0.055	0.999	0.000	0.999	0.998	0.999	0.999	0.999	0.999	0.999	0.999	0.996	0.999
	0.06	0.996	0.001	0.998	0.995	0.997	0.996	0.997	0.996	0.996	0.997	0.99	0.996
	0.065	0.990	0.001	0.994	0.989	0.993	0.99	0.992	0.991	0.991	0.992	0.978	0.991
	0.07	0.980	0.003	0.988	0.977	0.985	0.98	0.984	0.982	0.982	0.983	0.957	0.982
	0.075	0.961	0.004	0.973	0.955	0.97	0.96	0.966	0.964	0.963	0.965	0.925	0.964
	0.08	0.930	0.007	0.951	0.922	0.944	0.929	0.94	0.935	0.932	0.936	0.876	0.934
	0.085	0.891	0.009	0.919	0.878	0.91	0.888	0.904	0.897	0.895	0.9	0.818	0.896
	0.09	0.838	0.012	0.878	0.821	0.863	0.835	0.855	0.847	0.841	0.849	0.746	0.845
	0.095	0.774	0.015	0.824	0.753	0.805	0.769	0.796	0.784	0.778	0.787	0.661	0.782
	0.1	0.704	0.016	0.763	0.679	0.739	0.699	0.73	0.715	0.707	0.718	0.581	0.712
	0.105	0.626	0.018	0.69	0.598	0.663	0.619	0.654	0.637	0.629	0.642	0.494	0.633
	0.11	0.544	0.018	0.613	0.515	0.583	0.537	0.573	0.555	0.546	0.559	0.411	0.551
	0.115	0.466	0.018	0.536	0.436	0.504	0.459	0.495	0.477	0.466	0.481	0.338	0.472
	0.12	0.391	0.017	0.46	0.36	0.427	0.382	0.419	0.401	0.39	0.403	0.268	0.395
	0.125	0.323	0.015	0.389	0.295	0.355	0.315	0.348	0.332	0.32	0.334	0.213	0.326
	0.13	0.257	0.014	0.318	0.232	0.286	0.249	0.28	0.265	0.254	0.267	0.16	0.259
$N=16$	0.05	1.000	0.000	1	1	1	1	1	1	1	1	1	1
	0.055	1.000	0.000	1	1	1	1	1	1	1	1	1	1
	0.06	1.000	0.000	1	1	1	1	1	1	1	1	1	1
	0.065	1.000	0.000	1	1	1	1	1	1	1	1	1	1
	0.07	0.999	0.001	0.999	1	1	1	0.996	1	0.998	1	1	0.996
	0.075	0.996	0.002	0.997	1	0.998	1	0.987	0.998	0.993	1	1	0.985
	0.08	0.986	0.006	0.988	1	0.994	1	0.959	0.993	0.978	1	0.998	0.949
	0.085	0.963	0.015	0.965	0.999	0.98	1	0.901	0.979	0.941	1	0.993	0.874
	0.09	0.921	0.028	0.91	0.996	0.944	1	0.8	0.945	0.877	1	0.979	0.76
	0.095	0.848	0.049	0.797	0.99	0.876	0.999	0.652	0.87	0.763	0.999	0.947	0.583
	0.1	0.760	0.068	0.665	0.965	0.77	0.997	0.508	0.757	0.629	0.999	0.884	0.424
	0.105	0.650	0.087	0.495	0.919	0.623	0.987	0.35	0.604	0.481	0.995	0.78	0.269
	0.11	0.540	0.102	0.341	0.841	0.472	0.967	0.219	0.448	0.333	0.986	0.649	0.146
	0.115	0.440	0.109	0.215	0.729	0.321	0.929	0.133	0.301	0.216	0.971	0.503	0.083
	0.12	0.351	0.112	0.119	0.606	0.2	0.869	0.065	0.188	0.126	0.939	0.363	0.034
	0.125	0.277	0.108	0.06	0.463	0.116	0.78	0.032	0.104	0.071	0.888	0.239	0.015
	0.13	0.211	0.097	0.025	0.319	0.06	0.658	0.013	0.052	0.035	0.802	0.145	0.004

Table S12. Data values in Fig. 4E ($N = 1, 4, 16$), 10 independent training.

	p_{flip}	Mean of $\bar{y}$	Standard error	$\bar{y}$ for independent training									
$N = 64$	0.05	1.000	0.000	1	1	1	1	1	1	1	1	1	1
	0.055	1.000	0.000	1	1	1	1	1	1	1	1	1	1
	0.06	1.000	0.000	1	1	1	1	1	1	1	1	1	1
	0.065	1.000	0.000	1	1	1	1	1	1	1	1	1	1
	0.07	1.000	0.000	1	1	1	1	1	1	1	1	1	1
	0.075	1.000	0.000	1	1	1	1	1	1	1	1	1	1
	0.08	1.000	0.000	1	1	1	1	1	1	1	0.998	1	1
	0.085	0.998	0.002	1	1	1	0.999	1	1	1	0.984	1	1
	0.09	0.992	0.007	1	0.999	1	0.989	1	1	1	0.933	1	1
	0.095	0.959	0.030	0.999	0.98	0.998	0.909	1	0.996	1	0.712	0.996	1
	0.1	0.901	0.060	0.987	0.904	0.993	0.724	0.999	0.984	1	0.446	0.97	1
	0.105	0.794	0.092	0.919	0.677	0.951	0.438	0.991	0.909	1	0.194	0.864	1
	0.11	0.653	0.111	0.746	0.42	0.821	0.177	0.937	0.73	1	0.055	0.645	1
	0.115	0.499	0.118	0.495	0.196	0.59	0.06	0.79	0.471	1	0.018	0.373	0.999
	0.12	0.356	0.125	0.24	0.059	0.324	0.012	0.544	0.228	1	0.001	0.155	0.995
	0.125	0.262	0.130	0.087	0.012	0.131	0.003	0.277	0.091	1	0	0.047	0.975
	0.13	0.210	0.132	0.018	0.002	0.037	0.001	0.093	0.022	1	0	0.011	0.913
SMLP	0.05	0.937	0.025	0.965	0.931	0.751	0.917	0.985	0.983	0.976	0.875	0.995	0.991
	0.055	0.923	0.027	0.951	0.911	0.729	0.896	0.977	0.974	0.964	0.849	0.991	0.985
	0.06	0.904	0.029	0.932	0.886	0.706	0.87	0.965	0.961	0.944	0.819	0.985	0.975
	0.065	0.880	0.031	0.906	0.855	0.681	0.839	0.947	0.941	0.917	0.784	0.973	0.957
	0.07	0.851	0.031	0.875	0.82	0.657	0.805	0.921	0.913	0.88	0.748	0.955	0.932
	0.075	0.813	0.030	0.835	0.779	0.633	0.765	0.885	0.875	0.83	0.709	0.925	0.892
	0.08	0.765	0.028	0.785	0.731	0.607	0.721	0.835	0.823	0.768	0.667	0.879	0.835
	0.085	0.710	0.024	0.729	0.681	0.583	0.674	0.773	0.759	0.696	0.624	0.817	0.762
	0.09	0.646	0.018	0.666	0.627	0.558	0.624	0.699	0.684	0.616	0.581	0.734	0.675
	0.095	0.573	0.012	0.594	0.569	0.532	0.571	0.61	0.596	0.528	0.534	0.629	0.57
	0.1	0.504	0.009	0.526	0.514	0.509	0.521	0.523	0.51	0.447	0.493	0.523	0.471
	0.105	0.431	0.013	0.455	0.457	0.484	0.469	0.432	0.422	0.365	0.448	0.411	0.371
	0.11	0.365	0.020	0.386	0.403	0.46	0.418	0.346	0.339	0.295	0.407	0.311	0.284
	0.115	0.309	0.025	0.327	0.355	0.439	0.374	0.276	0.271	0.236	0.369	0.232	0.214
	0.12	0.260	0.029	0.272	0.308	0.417	0.33	0.215	0.212	0.185	0.334	0.167	0.158
	0.125	0.219	0.031	0.226	0.268	0.396	0.292	0.165	0.164	0.145	0.301	0.118	0.115
	0.13	0.183	0.032	0.182	0.229	0.375	0.254	0.124	0.123	0.111	0.269	0.081	0.081

Table S13. Data values in Fig. 4E ($N = 64$ and SMLP), 10 independent training.

	g_X	Mean of $\bar{y}$	Standard error	$\bar{y}$ for independent training									
$N = 1$	0.14	0.994	0.001	0.996	0.996	0.99	0.988	0.994	0.995	0.995	0.995	0.995	0.996
	0.16	0.981	0.002	0.987	0.986	0.97	0.965	0.982	0.983	0.984	0.983	0.984	0.985
	0.18	0.946	0.005	0.958	0.956	0.92	0.911	0.95	0.951	0.953	0.949	0.953	0.955
	0.2	0.867	0.010	0.888	0.882	0.82	0.805	0.878	0.877	0.883	0.876	0.88	0.884
	0.22	0.703	0.015	0.733	0.722	0.628	0.606	0.722	0.721	0.728	0.716	0.722	0.728
	0.24	0.417	0.017	0.447	0.434	0.33	0.31	0.443	0.438	0.449	0.433	0.44	0.447
	0.26	0.265	0.014	0.286	0.275	0.192	0.177	0.288	0.284	0.294	0.278	0.284	0.288
	0.28	0.172	0.011	0.186	0.178	0.114	0.102	0.192	0.188	0.196	0.183	0.187	0.191
	0.3	0.118	0.009	0.128	0.122	0.073	0.065	0.134	0.13	0.136	0.126	0.13	0.132
	0.32	0.081	0.007	0.088	0.084	0.047	0.041	0.094	0.091	0.096	0.088	0.09	0.092
	0.34	0.058	0.005	0.063	0.058	0.032	0.028	0.068	0.065	0.07	0.063	0.065	0.067
$N = 4$	0.14	1.000	0.000	1	1	1	1	1	1	1	1	1	1
	0.16	1.000	0.000	1	1	1	1	1	1	1	1	1	1
	0.18	1.000	0.000	1	1	1	1	1	1	1	1	1	1
	0.2	0.999	0.000	0.999	0.998	0.999	0.999	0.999	0.999	0.999	0.999	0.996	0.999
	0.22	0.962	0.004	0.974	0.959	0.97	0.962	0.968	0.965	0.963	0.965	0.931	0.965
	0.24	0.656	0.016	0.714	0.632	0.691	0.65	0.683	0.666	0.655	0.672	0.532	0.664
	0.26	0.336	0.015	0.401	0.31	0.369	0.327	0.362	0.345	0.334	0.346	0.228	0.34
	0.28	0.150	0.009	0.193	0.134	0.169	0.143	0.167	0.155	0.148	0.156	0.088	0.151
	0.3	0.061	0.005	0.086	0.052	0.07	0.057	0.069	0.063	0.058	0.062	0.031	0.06
	0.32	0.022	0.002	0.036	0.017	0.026	0.02	0.027	0.023	0.02	0.023	0.008	0.021
	0.34	0.009	0.001	0.016	0.007	0.011	0.008	0.011	0.01	0.009	0.01	0.003	0.009
$N = 16$	0.14	1.000	0.000	1	1	1	1	1	1	1	1	1	1
	0.16	1.000	0.000	1	1	1	1	1	1	1	1	1	1
	0.18	1.000	0.000	1	1	1	1	1	1	1	1	1	1
	0.2	1.000	0.000	1	1	1	1	1	1	1	1	1	1
	0.22	0.997	0.002	0.994	1	0.999	1	0.991	1	0.996	1	1	0.987
	0.24	0.692	0.078	0.539	0.92	0.666	0.991	0.433	0.653	0.546	0.997	0.83	0.343
	0.26	0.287	0.106	0.07	0.479	0.133	0.786	0.04	0.119	0.079	0.884	0.256	0.022
	0.28	0.103	0.063	0.002	0.101	0.005	0.333	0.002	0.003	0.004	0.552	0.026	0.002
	0.3	0.034	0.024	0	0.013	0	0.102	0	0	0	0.22	0.004	0
	0.32	0.009	0.008	0	0.002	0	0.02	0	0	0	0.072	0	0
	0.34	0.002	0.002	0	0	0	0.004	0	0	0	0.016	0	0
$N = 64$	0.14	1.000	0.000	1	1	1	1	1	1	1	1	1	1
	0.16	1.000	0.000	1	1	1	1	1	1	1	1	1	1
	0.18	1.000	0.000	1	1	1	1	1	1	1	1	1	1
	0.2	1.000	0.000	1	1	1	1	1	1	1	1	1	1
	0.22	1.000	0.000	1	1	1	1	1	1	1	1	1	1
	0.24	0.850	0.076	0.942	0.778	0.99	0.609	0.999	0.958	1	0.307	0.914	1
	0.26	0.271	0.129	0.101	0.016	0.139	0.001	0.315	0.101	1	0	0.061	0.974
	0.28	0.150	0.113	0	0	0	0	0	0	0.999	0	0	0.503
	0.3	0.104	0.103	0	0	0	0	0	0	0.985	0	0	0.056
	0.32	0.085	0.089	0	0	0	0	0	0	0.846	0	0	0.001
	0.34	0.040	0.042	0	0	0	0	0	0	0.395	0	0	0
SMLP	0.14	0.977	0.015	0.995	0.986	0.854	0.98	0.999	0.998	0.998	0.964	1	1
	0.16	0.971	0.017	0.992	0.978	0.83	0.971	0.998	0.997	0.997	0.948	1	0.999
	0.18	0.959	0.021	0.984	0.962	0.795	0.951	0.994	0.993	0.991	0.92	0.999	0.997
	0.2	0.932	0.026	0.96	0.923	0.742	0.909	0.982	0.979	0.972	0.865	0.994	0.989
	0.22	0.843	0.031	0.866	0.812	0.652	0.797	0.913	0.904	0.872	0.74	0.949	0.923
	0.24	0.463	0.010	0.486	0.484	0.494	0.491	0.47	0.459	0.402	0.467	0.46	0.416
	0.26	0.223	0.031	0.229	0.271	0.398	0.295	0.17	0.168	0.151	0.303	0.123	0.118
	0.28	0.125	0.032	0.11	0.158	0.332	0.183	0.063	0.064	0.061	0.206	0.033	0.036
	0.3	0.082	0.028	0.06	0.098	0.282	0.121	0.028	0.029	0.029	0.148	0.012	0.014
	0.32	0.060	0.025	0.036	0.067	0.247	0.085	0.014	0.015	0.016	0.111	0.005	0.007
	0.34	0.046	0.022	0.022	0.046	0.218	0.061	0.008	0.009	0.009	0.086	0.002	0.003

Table S14. Data values in Fig. 4F, 10 independent training.

	p_{flip}	Mean of $\bar{y}$	Standard error	$\bar{y}$ for independent training									
PAB	0.05	1.000	0.000	1	1	1	1	1	1	1	1	1	1
	0.055	1.000	0.000	1	1	1	1	1	1	1	1	1	1
	0.06	1.000	0.000	1	1	1	1	1	1	1	1	1	1
	0.065	1.000	0.000	1	1	1	1	1	1	1	1	1	1
	0.07	1.000	0.000	1	1	1	1	1	1	1	1	1	1
	0.075	1.000	0.000	1	1	1	1	1	1	1	0.998	1	1
	0.08	0.998	0.002	1	1	1	1	1	1	0.997	0.978	1	1
	0.085	0.985	0.013	1	1	1	1	1	1	0.971	0.876	1	1
	0.09	0.952	0.039	1	0.999	1	0.999	1	1	0.89	0.635	1	1
	0.095	0.891	0.078	0.999	0.988	0.995	0.991	0.994	0.999	0.639	0.307	1	0.998
	0.1	0.826	0.107	0.999	0.943	0.97	0.945	0.966	0.997	0.352	0.103	1	0.986
	0.105	0.735	0.117	0.982	0.79	0.862	0.804	0.847	0.981	0.146	0.026	1	0.907
	0.11	0.596	0.113	0.909	0.551	0.641	0.556	0.626	0.913	0.038	0.005	0.997	0.722
	0.115	0.428	0.105	0.749	0.316	0.374	0.311	0.357	0.746	0.009	0.001	0.979	0.433
	0.12	0.265	0.095	0.497	0.13	0.168	0.132	0.141	0.491	0.002	0	0.915	0.177
	0.125	0.151	0.078	0.255	0.041	0.049	0.046	0.05	0.258	0.001	0	0.765	0.049
	0.13	0.074	0.051	0.089	0.011	0.014	0.012	0.01	0.091	0	0	0.497	0.011
	0.135	0.035	0.029	0.029	0.002	0.002	0.003	0.002	0.03	0	0	0.283	0.002
	0.14	0.013	0.012	0.007	0	0	0	0	0.006	0	0	0.116	0
	0.145	0.004	0.004	0.001	0	0	0	0	0.001	0	0	0.042	0
	0.15	0.001	0.001	0	0	0	0	0	0	0	0	0.01	0
QuAN2	0.05	1.000	0.000	1	1	1	1	1	1	1	1	1	1
	0.055	1.000	0.000	1	1	1	1	1	1	1	1	1	1
	0.06	1.000	0.000	1	1	1	1	1	1	1	1	1	1
	0.065	1.000	0.000	1	1	1	1	1	1	1	1	1	1
	0.07	1.000	0.000	1	1	1	1	1	1	1	1	1	1
	0.075	1.000	0.000	1	1	1	1	1	1	1	1	1	1
	0.08	1.000	0.000	1	1	1	1	1	1	1	0.998	1	1
	0.085	0.998	0.002	1	1	1	0.999	1	1	1	0.984	1	1
	0.09	0.992	0.007	1	0.999	1	0.989	1	1	1	0.933	1	1
	0.095	0.959	0.030	0.999	0.98	0.998	0.909	1	0.996	1	0.712	0.996	1
	0.1	0.901	0.060	0.987	0.904	0.993	0.724	0.999	0.984	1	0.446	0.97	1
	0.105	0.794	0.092	0.919	0.677	0.951	0.438	0.991	0.909	1	0.194	0.864	1
	0.11	0.653	0.111	0.746	0.42	0.821	0.177	0.937	0.73	1	0.055	0.645	1
	0.115	0.499	0.118	0.495	0.196	0.59	0.06	0.79	0.471	1	0.018	0.373	0.999
	0.12	0.356	0.125	0.24	0.059	0.324	0.012	0.544	0.228	1	0.001	0.155	0.995
	0.125	0.262	0.130	0.087	0.012	0.131	0.003	0.277	0.091	1	0	0.047	0.975
	0.13	0.210	0.132	0.018	0.002	0.037	0.001	0.093	0.022	1	0	0.011	0.913
	0.135	0.183	0.126	0.004	0	0.008	0	0.029	0.004	1	0	0.003	0.782
	0.14	0.158	0.115	0.001	0	0.002	0	0.008	0.002	0.999	0	0.001	0.565
	0.145	0.136	0.108	0	0	0	0	0.001	0	0.999	0	0	0.364
	0.15	0.118	0.105	0	0	0	0	0	0	0.998	0	0	0.181

Table S15. Data values in Fig. 4G, 10 independent training.

	g_X	Mean of $\bar{y}$	Standard error	$\bar{y}$ for independent training									
PAB	0.14	1.000	0.000	1	1	1	1	1	1	1	1	1	1
	0.16	1.000	0.000	1	1	1	1	1	1	1	1	1	1
	0.18	1.000	0.000	1	1	1	1	1	1	1	1	1	1
	0.2	1.000	0.000	1	1	1	1	1	1	1	1	1	1
	0.22	1.000	0.000	1	1	1	1	1	1	1	0.999	1	1
	0.24	0.775	0.113	0.991	0.845	0.922	0.875	0.891	0.991	0.236	0.053	1	0.948
	0.26	0.163	0.080	0.28	0.051	0.059	0.053	0.059	0.278	0.001	0	0.784	0.067
	0.28	0.004	0.004	0.002	0	0	0	0	0	0	0	0.038	0
	0.3	0.000	0.000	0	0	0	0	0	0	0	0	0.001	0
	0.32	0.000	0.000	0	0	0	0	0	0	0	0	0	0
	0.34	0.000	0.000	0	0	0	0	0	0	0	0	0	0
QuAN2	0.14	1.000	0.000	1	1	1	1	1	1	1	1	1	1
	0.16	1.000	0.000	1	1	1	1	1	1	1	1	1	1
	0.18	1.000	0.000	1	1	1	1	1	1	1	1	1	1
	0.2	1.000	0.000	1	1	1	1	1	1	1	1	1	1
	0.22	1.000	0.000	1	1	1	1	1	1	1	1	1	1
	0.24	0.850	0.076	0.942	0.778	0.99	0.609	0.999	0.958	0.999	0.307	0.914	1
	0.26	0.269	0.128	0.101	0.016	0.139	0.001	0.315	0.101	0.985	0	0.061	0.974
	0.28	0.135	0.099	0	0	0	0	0	0	0.846	0	0	0.503
	0.3	0.045	0.041	0	0	0	0	0	0	0.395	0	0	0.056
	0.32	0.006	0.006	0	0	0	0	0	0	0.056	0	0	0.001
	0.34	0.000	0.000	0	0	0	0	0	0	0.001	0	0	0

Table S16. Data values in Fig. 4H, 10 independent training.

Loop perimeter	Mean of $\bar{y}$	Standard error	$\langle Z_{closed} \rangle$ for 10 high attention score snapshos									
4	0.882	0.038	0.83	1	1	0.94	0.72	0.89	0.72	0.94	1	0.78
8	0.792	0.076	0.76	1	1	0.92	0.36	0.92	0.6	0.84	1	0.52
12	0.713	0.099	0.5	1	1	0.88	0.5	0.88	0.5	0.75	1	0.12
16	0.534	0.154	0.11	1	1	0.78	-0.33	0.78	0.11	0.56	1	0.33
20	0.500	0.136	0.5	1	1	0.5	0	0.5	0.5	0	1	0
24	0.000	0.351	-1	1	1	-1	-1	1	-1	-1	1	1
**Loop perimeter**	**Mean of $\bar{y}$**	**Standard error**	**$\langle Z_{closed} \rangle$ for 10 low attention score snapshos**									
4	0.511	0.029	0.44	0.5	0.39	0.67	0.56	0.5	0.44	0.56	0.44	0.61
8	0.088	0.077	0.36	-0.28	-0.12	0.36	-0.12	-0.04	0.12	0.36	0.2	0.04
12	0.086	0.079	-0.38	0.25	0	0.12	0.25	-0.12	0	0.12	0.12	0.5
16	0.134	0.113	-0.11	-0.33	0.11	0.78	0.11	-0.11	0.11	0.33	-0.11	0.56
20	0.100	0.205	0	0	0	1	0	-0.5	0.5	0	-1	1
24	0.200	0.344	1	-1	-1	1	1	-1	1	1	1	-1

Table S17. Data values in Fig. 4J.

REFERENCES AND NOTES

1. D. Bluvstein, S. J. Evered, A. A. Geim, S. H. Li, H. Zhou, T. Manovitz, S. Ebadi, M. Cain, M. Kalinowski, D. Hangleiter, J. P. Bonilla Ataides, N. Maskara, I. Cong, X. Gao, P. Sales Rodriguez, T. Karolyshyn, G. Semeghini, M. J. Gullans, M. Greiner, V. Vuletić, M. D. Lukin, Logical quantum processor based on reconfigurable atom arrays. *Nature* **626**, 58–65 (2024).

2. Google Quantum AI, Suppressing quantum errors by scaling a surface code logical qubit. *Nature* **614**, 676–681 (2023).

3. Z. Ni, S. Li, X. Deng, Y. Cai, L. Zhang, W. Wang, Z.-B. Yang, H. Yu, F. Yan, S. Liu, C.-L. Zou, L. Sun, S.-B. Zheng, Y. Xu, D. Yu, Beating the break-even point with a discrete-variable-encoded logical qubit. *Nature* **616**, 56–60 (2023).

4. A. Vaswani, N. Shazeer, N. Parmar, J. Uszkoreit, L. Jones, A. N. Gomez, L. Kaiser, I. Polosukhin, “Attention is all you need,” in *Proceedings of the 31st International Conference on Neural Information Processing Systems* (Curran Associates Inc., 2017), p. 6000–6010; https://proceedings.neurips.cc/paper_files/paper/2017/file/3f5ee243547dee91fbd053c1c4a845aa-Paper.pdf.

5. A. Parikh, O. Täckström, D. Das, J. Uszkoreit, A decomposable attention model for natural language inference. arXiv:1606.01933 (2016).

6. L. Susskind, Computational complexity and black hole horizons. *Fortschr. Phys.* **64**, 24–43 (2016).

7. A. R. Brown, L. Susskind, Second law of quantum complexity. *Phys. Rev. D* **97**, 086015 (2018).

8. A. Bouland, B. Fefferman, U. Vazirani, Computational pseudorandomness, the wormhole growth paradox, and constraints on the AdS/CFT duality. arXiv:1910.14646 (2019).

9. S. Aaronson, Y. Atia, L. Susskind, On the hardness of detecting macroscopic superpositions. arXiv:2009.07450 (2020).

10. D. Gross, S. T. Flammia, J. Eisert, Most quantum states are too entangled to be useful as computational resources. *Phys. Rev. Lett.* **102**, 190501 (2009).

11. D. Girolami, F. Anzà, Quantifying the difference between many-body quantum states. *Phys. Rev. Lett.* **126**, 170502 (2021).

12. M. B. Hastings, Topological order at nonzero temperature. *Phys. Rev. Lett.* **107**, 210501 (2011).

13. M. Schwarz, K. Temme, F. Verstraete, D. Perez-Garcia, T. S. Cubitt, Preparing topological projected entangled pair states on a quantum computer. *Phys. Rev. A* **88**, 032321 (2013).

14. J. Miller, A. Miyake, Latent computational complexity of symmetry-protected topological order with fractional symmetry. *Phys. Rev. Lett.* **120**, 170503 (2018).

15. F. G. Brandão, W. Chemissany, N. Hunter-Jones, R. Kueng, J. Preskill, Models of quantum complexity growth. *PRX Quantum* **2**, 030316 (2021).

16. J. Haferkamp, P. Faist, N. B. T. Kothakonda, J. Eisert, N. Yunger Halpern, Linear growth of quantum circuit complexity. *Nat. Phys.* **18**, 528–532 (2022).

17. K. Kaneko, E. Iyoda, T. Sagawa, Characterizing complexity of many-body quantum dynamics by higher-order eigenstate thermalization. *Phys. Rev. A* **101**, 042126 (2020).

18. E. Bianchi, L. Hackl, M. Kieburg, M. Rigol, L. Vidmar, Volume-law entanglement entropy of typical pure quantum states *PRX Quantum* **3**, 030201 (2022).

19. J. Wang, M. H. Lamann, J. Richter, R. Steinigeweg, A. Dymarsky, J. Gemmer, Eigenstate thermalization hypothesis and its deviations from random-matrix theory beyond the thermalization time. *Phys. Rev. Lett.* **128**, 180601 (2022).

20. B. D. S. L. Torres, E. Martín-Martínez, Toward a physically motivated notion of Gaussian complexity geometry. *Phys. Rev. D* **110**, 025006 (2024).

21. H. A. Camargo, L. Hackl, M. P. Heller, A. Jahn, T. Takayanagi, B. Windt, Entanglement and complexity of purification in (1+1)-dimensional free conformal field theories. *Phys. Rev. Res.* **3**, 013248 (2021).

22. N. Yunger Halpern, N. B. T. Kothakonda, J. Haferkamp, A. Munson, J. Eisert, P. Faist, Resource theory of quantum uncomplexity. *Phys. Rev. A* **106**, 062417 (2022).

23. *C. Miles*, R. Samajdar, S. Ebadi, T. T. Wang, H. Pichler, S. Sachdev, M. D. Lukin, M. Greiner, K. Q. Weinberger, E.-A. Kim, Machine learning discovery of new phases in programmable quantum simulator snapshots. *Phys. Rev. Res.* **5**, 013026 (2023).

24. H.-Y. Huang, R. Kueng, G. Torlai, V. V. Albert, J. Preskill, Provably efficient machine learning for quantum many-body problems. *Science* **377**, eabk3333 (2022).

25. *C. Miles*, A. Bohrdt, R. Wu, C. Chiu, M. Xu, G. Ji, M. Greiner, K. Q. Weinberger, E. Demler, E.-A. Kim, Correlator convolutional neural networks as an interpretable architecture for image-like quantum matter data. *Nat. Commun.* **12**, 3905 (2021).

26. J. Carrasquilla, Machine learning for quantum matter. *Adv. Phys. X.* **5**, 1797528 (2020).

27. Y. Zhang, P. Ginsparg, E.-A. Kim, Interpreting machine learning of topological quantum phase transitions. *Phys. Rev. Res.* **2**, 023283 (2020).

28. Y. Zhang, R. G. Melko, E.-A. Kim, Machine learning $\mathbb{Z}_2$ quantum spin liquids with quasiparticle statistics. *Phys. Rev. B* **96**, 245119 (2017).

29. Y. Zhang, E.-A. Kim, Quantum loop topography for machine learning. *Phys. Rev. Lett.* **118**, 216401 (2017).

30. S. Ahmed, C. Sánchez Muñoz, F. Nori, A. F. Kockum, Quantum state tomography with conditional generative adversarial networks. *Phys. Rev. Lett.* **127**, 140502 (2021).

31. M. Y. Niu, A. M. Dai, L. Li, A. Odena, Z. Zhao, V. Smelyanskyi, H. Neven, S. Boixo, Learnability and complexity of quantum samples. arXiv:2010.11983 (2020).

32. P. Cha, P. Ginsparg, F. Wu, J. Carrasquilla, P. L. McMahon, E.-A. Kim, Attention-based quantum tomography. *Mach. Learn. Sci. Technol.* **3**, 01LT01 (2021).

33. J. Carrasquilla, G. Torlai, R. G. Melko, L. Aolita, Reconstructing quantum states with generative models. *Nat. Mach. Intell.* **1**, 155–161 (2019).

34. J. Carrasquilla, D. Luo, F. Pérez, A. Milsted, B. K. Clark, M. Volkovs, L. Aolita, Probabilistic simulation of quantum circuits using a deep-learning architecture. *Phys. Rev. A* **104**, 032610 (2021).

35. Y.-H. Zhang, M. Di Ventra, Transformer quantum state: A multipurpose model for quantum many-body problems. *Phys. Rev. B* **107**, 075147 (2023).

36. F. G. S. L. Brandão, A. W. Harrow, M. Horodecki, Local random quantum circuits are approximate polynomial-designs. *Commun. Math. Phys.* **346**, 397–434 (2016).

37. J. Haferkamp, Random quantum circuits are approximate unitary t-designs in depth $O\left(nt^{5+o(1)}\right)$. arXiv:2203.16571 (2022).

38. J. Lee, Y. Lee, J. Kim, A. Kosiorek, S. Choi, Y. W. Teh, "Set transformer: A framework for attention-based permutation-invariant neural networks," in *Proceedings of the 36th International Conference on Machine Learning* (PMLR, 2019), pp. 3744–3753; https://proceedings.mlr.press/v97/lee19d.html.

39. D. N. Page, Average entropy of a subsystem. *Phys. Rev. Lett.* **71**, 1291–1294 (1993).

40. Y. Li, X. Chen, M. P. A. Fisher, Measurement-driven entanglement transition in hybrid quantum circuits. *Phys. Rev. B* **100**, 134306 (2019).

41. B. Skinner, J. Ruhman, A. Nahum, Measurement-induced phase transitions in the dynamics of entanglement. *Phys. Rev. X* **9**, 031009 (2019).

42. C.-M. Jian, Y.-Z. You, R. Vasseur, A. W. W. Ludwig, Measurement-induced criticality in random quantum circuits. *Phys. Rev. B* **101**, 104302 (2020).

43. M. J. Gullans, D. A. Huse, Dynamical purification phase transition induced by quantum measurements. *Phys. Rev. X* **10**, 041020 (2020).

44. S. Choi, Y. Bao, X.-L. Qi, E. Altman, Quantum error correction in scrambling dynamics and measurement-induced phase transition. *Phys. Rev. Lett.* **125**, 030505 (2020).

45. A. H. Karamlou, I. T. Rosen, S. E. Muschinske, C. N. Barrett, A. Di Paolo, L. Ding, P. M. Harrington, M. Hays, R. Das, D. K. Kim, B. M. Niedzielski, M. Schuldt, K. Serniak, M. E. Schwartz, J. L. Yoder, S. Gustavsson, Y. Yanay, J. A. Grover, W. D. Oliver, Probing entanglement in a 2D hard-core Bose-Hubbard lattice. *Nature* **629**, 561–566 (2024).

46. M. M. Wolf, F. Verstraete, M. B. Hastings, J. I. Cirac, Area laws in quantum systems: Mutual information and correlations. *Phys. Rev. Lett.* **100**, 070502 (2008).

47. I. Goodfellow, Y. Bengio, A. Courville, *Deep Learning* (MIT Press, 2016); http://www.deeplearningbook.org.

48. P. Hayden, J. Preskill, Black holes as mirrors: Quantum information in random subsystems. *J. High Energy Phys.* **2007**, 120–120 (2007).

49. A. Nahum, J. Ruhman, S. Vijay, J. Haah, Quantum entanglement growth under random unitary dynamics. *Phys. Rev. X* **7**, 031016 (2017).

50. C. W. Von Keyserlingk, T. Rakovszky, F. Pollmann, S. L. Sondhi, Operator hydrodynamics, OTOCs, and entanglement growth in systems without conservation laws. *Phys. Rev. X* **8**, 021013 (2018).

51. A. Nahum, S. Vijay, J. Haah, Operator spreading in random unitary circuits. *Phys. Rev. X* **8**, 021014 (2018).

52. E. Dennis, A. Kitaev, A. Landahl, J. Preskill, Topological quantum memory. *J. Math. Phys.* **43**, 4452–4505 (2002).

53. Y. Bao, R. Fan, A. Vishwanath, E. Altman, Mixed-state topological order and the errorfield double formulation of decoherence-induced transitions. arXiv:2301.05687 (2023).

54. R. Fan, Y. Bao, E. Altman, A. Vishwanath, Diagnostics of mixed-state topological order and breakdown of quantum memory. *PRX Quantum* **5**, 020343 (2024).

55. Y.-H. Chen, T. Grover, Unconventional topological mixed-state transition and critical phase induced by self-dual coherent errors. *Phys. Rev. B* **110**, 125152 (2024).

56. R. Sohal, A. Prem, Noisy approach to intrinsically mixed-state topological order. *PRX Quantum* **6**, 010313 (2025).

57. M. B. Hastings, X.-G. Wen, Quasiadiabatic continuation of quantum states: The stability of topological ground-state degeneracy and emergent gauge invariance. *Phys. Rev. B* **72**, 045141 (2005).

58. C. Castelnovo, C. Chamon, Topological order and topological entropy in classical systems. *Phys. Rev. B* **76**, 174416 (2007).

59. A. Honecker, M. Picco, P. Pujol, Universality class of the Nishimori point in the 2D $\pm J$ random-bond Ising model. *Phys. Rev. Lett.* **87**, 047201 (2001).

60. N. Maskara, "Enhancing detection of topological order by local error correction" (2023); https://osf.io/k8up2/.

61. I. Cong, N. Maskara, M. C. Tran, H. Pichler, G. Semeghini, S. F. Yelin, S. Choi, M. D. Lukin, Enhancing detection of topological order by local error correction. *Nat. Commun.* **15**, 1527 (2024).

62. C. Castelnovo, C. Chamon, Quantum topological phase transition at the microscopic level. *Phys. Rev. B* **77**, 054433 (2008).

63. C. N. Barrett, A. H. Karamlou, S. E. Muschinske, I. T. Rosen, J. Braumüller, R. Das, D. K. Kim, B. M. Niedzielski, M. Schuldt, K. Serniak, M. E. Schwartz, J. L. Yoder, T. P. Orlando, S.

Gustavsson, J. A. Grover, W. D. Oliver, Learning-based calibration of flux crosstalk in transmon qubit arrays. *Phys. Rev. Appl.* **20**, 024070 (2023).

64. F. Arute, K. Arya, R. Babbush, D. Bacon, J. C. Bardin, R. Barends, R. Biswas, S. Boixo, F. G. S. L. Brandao, D. A. Buell, B. Burkett, Y. Chen, Z. Chen, B. Chiaro, R. Collins, W. Courtney, A. Dunsworth, E. Farhi, B. Foxen, A. Fowler, C. Gidney, M. Giustina, R. Graff, K. Guerin, S. Habegger, M. P. Harrigan, M. J. Hartmann, A. Ho, M. Hoffmann, T. Huang, T. S. Humble, S. V. Isakov, E. Jeffrey, Z. Jiang, D. Kafri, K. Kechedzhi, J. Kelly, P. V. Klimov, S. Knysh, A. Korotkov, F. Kostritsa, D. Landhuis, M. Lindmark, E. Lucero, D. Lyakh, S. Mandrà, J. R. McClean, M. McEwen, A. Megrant, X. Mi, K. Michielsen, M. Mohseni, J. Mutus, O. Naaman, M. Neeley, C. Neill, M. Y. Niu, E. Ostby, A. Petukhov, J. C. Platt, C. Quintana, E. G. Rieffel, P. Roushan, N. C. Rubin, D. Sank, K. J. Satzinger, V. Smelyanskiy, K. J. Sung, M. D. Trevithick, A. Vainsencher, B. Villalonga, T. White, Z. J. Yao, P. Yeh, A. Zalcman, H. Neven, J. M. Martinis, Quantum supremacy using a programmable superconducting processor. *Nature* **574**, 505–510 (2019).

65. Google Quantum AI and Collaborators, Measurement-induced entanglement and teleportation on a noisy quantum processor. *Nature* **622**, 481–486 (2023).

66. A. Morvan, B. Villalonga, X. Mi, S. Mandrà, A. Bengtsson, P. V. Klimov, Z. Chen, S. Hong, C. Erickson, I. K. Drozdov, J. Chau, G. Laun, R. Movassagh, A. Asfaw, L. T. A. N. Brandão, *R. Peralta*, D. Abanin, R. Acharya, R. Allen, T. I. Andersen, K. Anderson, M. Ansmann, F. Arute, K. Arya, J. Atalaya, J. C. Bardin, A. Bilmes, G. Bortoli, A. Bourassa, J. Bovaird, L. Brill, M. Broughton, B. B. Buckley, D. A. Buell, T. Burger, B. Burkett, N. Bushnell, J. Campero, H.-S. Chang, B. Chiaro, D. Chik, C. Chou, J. Cogan, R. Collins, P. Conner, W. Courtney, A. L. Crook, B. Curtin, D. M. Debroy, A. D. T. Barba, S. Demura, A. D. Paolo, A. Dunsworth, L. Faoro, E. Farhi, R. Fatemi, V. S. Ferreira, L. F. Burgos, E. Forati, A. G. Fowler, B. Foxen, G. Garcia, É. Genois, W. Giang, C. Gidney, D. Gilboa, M. Giustina, R. Gosula, A. G. Dau, J. A. Gross, S. Habegger, M. C. Hamilton, M. Hansen, M. P. Harrigan, S. D. Harrington, P. Heu, M. R. Hoffmann, T. Huang, A. Huff, W. J. Huggins, L. B. Ioffe, S. V. Isakov, J. Iveland, E. Jeffrey, Z. Jiang, C. Jones, P. Juhas, D. Kafri, T. Khattar, M. Khezri, M. Kieferová, S. Kim, A. Kitaev, A. R. Klots, A. N. Korotkov, F. Kostritsa, J. M. Kreikebaum, D. Landhuis, P. Laptev, K.-M. Lau, L.

Laws, J. Lee, K. W. Lee, Y. D. Lensky, B. J. Lester, A. T. Lill, W. Liu, W. P. Livingston, A. Locharla, F. D. Malone, O. Martin, S. Martin, J. R. McClean, M. McEwen, K. C. Miao, A. Mieszala, S. Montazeri, W. Mruczkiewicz, O. Naaman, M. Neeley, C. Neill, A. Nersisyan, M. Newman, J. H. Ng, A. Nguyen, M. Nguyen, M. Y. Niu, T. E. O'Brien, S. Omonije, A. Opremcak, A. Petukhov, R. Potter, L. P. Pryadko, C. Quintana, D. M. Rhodes, C. Rocque, E. Rosenberg, N. C. Rubin, N. Saei, D. Sank, K. Sankaragomathi, K. J. Satzinger, H. F. Schurkus, C. Schuster, M. J. Shearn, A. Shorter, N. Shutty, V. Shvarts, V. Sivak, J. Skruzny, W. C. Smith, R. D. Somma, G. Sterling, D. Strain, M. Szalay, D. Thor, A. Torres, G. Vidal, C. V. Heidweiller, T. White, B. W. K. Woo, C. Xing, Z. J. Yao, P. Yeh, J. Yoo, G. Young, A. Zalcman, Y. Zhang, N. Zhu, N. Zobrist, E. G. Rieffel, R. Biswas, R. Babbush, D. Bacon, J. Hilton, E. Lucero, H. Neven, A. Megrant, J. Kelly, P. Roushan, I. Aleiner, V. Smelyanskiy, K. Kechedzhi, Y. Chen, S. Boixo, Phase transitions in random circuit sampling. *Nature* **634**, 328–333 (2024).

67. Cirq Developers, Cirq (2023); https://zenodo.org/records/10247207.

68. M. Zaheer, S. Kottur, S. Ravanbakhsh, B. Poczos, R. R. Salakhutdinov, A. J. Smola, "Deep sets," in *Advances in Neural Information Processing Systems* (Curran Associates Inc., 2017), vol. 30; https://proceedings.neurips.cc/paper_files/paper/2017/file/f22e4747da1aa27e363d86d40ff442fe-Paper.pdf.
